# Involvement of the miR156/SPL module in flooding response in *Medicago sativa*

**DOI:** 10.1038/s41598-021-82450-7

**Published:** 2021-02-05

**Authors:** Biruk A. Feyissa, Lisa Amyot, Vida Nasrollahi, Yousef Papadopoulos, Susanne E. Kohalmi, Abdelali Hannoufa

**Affiliations:** 1 Agriculture and Agri-Food Canada, 1391 Sandford Street, London, ON N5V 4T3 Canada; 2 Department of Biology, University of Western Ontario, 1151 Richmond Street, London, ON N6A5B7 Canada; 3 Agriculture and Agri-Food Canada, 58 River Road, Truro, NS B2N 5E3 Canada

**Keywords:** Plant sciences, Abiotic

## Abstract

The highly conserved plant microRNA, miR156, affects plant development, metabolite composition, and stress response. Our previous research revealed the role of miR156 in abiotic stress response in *Medicago sativa* exerted by downregulating *SQUAMOSA-PROMOTER BINDING PROTEIN-LIKE* transcription factors. Here we investigated the involvement and possible mechanism of action of the miR156/SPL module in flooding tolerance in alfalfa. For that, we used *miR156* overexpressing, *SPL13*RNAi, flood-tolerant (AAC-Trueman) and -sensitive (AC-Caribou) alfalfa cultivars exposed to flooding. We also used *Arabidopsis* ABA insensitive (*abi1-2, abi5-8*) mutants and transgenic lines with either overexpressed (KIN10-OX1, KIN10-OX2) or silenced (KIN10RNAi-1, KIN10RNAi-2) catalytic subunit of SnRK1 to investigate a possible role of ABA and SnRK1 in regulating *miR156* expression under flooding. Physiological analysis, hormone profiling and global transcriptome changes revealed a role for *miR156*/SPL module in flooding tolerance. We also identified nine novel alfalfa SPLs (SPL1, SPL1a, SPL2a, SPL7, SPL7a, SPL8, SPL13a, SPL14, SPL16) responsive to flooding. Our results also showed a possible ABA-dependent SnRK1 upregulation to enhance *miR156* expression, resulting in downregulation of *SPL4, SPL7a, SPL8, SPL9, SPL13,* and *SPL13a*. We conclude that these effects induce flooding adaptive responses in alfalfa and modulate stress physiology by affecting the transcriptome, ABA metabolites and secondary metabolism.

## Introduction

Climate change is expected to increase the mean annual temperature and precipitation, which are correlated with flooding events^1–3^, affecting crop quality and yield^4,5^. Plants use escape or adaptive mechanisms to cope with flooding stress (FS). For example, flood-escaping rice genotypes elongate plant height by increasing stem cell size^6^ maintaining leaf gas exchange. *SUBMERGENCE1A* (*SUB1A*), *SNORKEL1* (*SK1*) and *SK2* were identified in *Arabidopsis thaliana* through their sequence similarity to rice homologs and were found to play a major role in flooding response^7,8^. Unlike plants employing the energy demanding flood-escape strategy, flood-adapting plants, such as wheat, reduce their energy metabolism, remaining submerged, and revive after the stress has ceased to exist^9^.

Regulating energy metabolism is important, especially under stress during which a reduced photosynthesis rate decreases glucose levels, despite the increased demand for carbon skeletons to biosynthesize specialized metabolites to scavenge Reactive Oxygen Species. Reduced energy metabolism (REM) associated with decreased oxygen availability and reduced photosynthesis assimilation during FS has been reported in different studies^10–12^. REM is perceived by a sucrose non-fermenting-related protein kinase, SnRK1^13,14^, which is associated with a hexokinase1 (HXK1)^15^. The heterotrimeric protein kinase SnRK1 is catalyzed by the α subunits KIN10 and KIN11^16^ and regulated by the β and γ subunits in *Arabidopsis*^17^. *SnRK1* expression is also triggered in *Arabidopsis* by the stress-related hormone abscisic acid^11,18^. The activation of SnRK1 regulates metabolic stress response and development in *Arabidopsis*^14^.

Previous studies showed that overexpression of *microRNA156* in *Medicago sativa* (alfalfa) increased shoot branching, delayed flowering, reduced stem length^19–21^, and played a positive role in abiotic stress tolerance^22–26^. Considering these miR156 functions, we hypothesised that miR156 might play a role in flooding tolerance and its expression might be regulated by SnRK1. Non-protein-coding RNA sequencing revealed multiple differentially expressed microRNAs in response to flooding in poplar and maize^27,28^. In maize, Zhang et al*.*^28^ identified over 100 differentially expressed microRNAs upon FS of which miR159, miR395, and miR474 were increased while others (miR166, miR167, miR171, miR396, miR399) were decreased. Besides screening for differentially expressed microRNAs, it is important to validate microRNAs and their target genes by modulating their expression and investigating the plant’s response to FS. In plants, miR156 down regulates *SQUAMOSA-PROMOTER BINDING PROTEIN-LIKE (SPL)* genes^29^. So far, at least seven *SPL*s (*SPL2, SPL3, SPL4, SPL6, SPL9, SPL12, SPL13*) have been identified as direct targets of miR156 in alfalfa^30^. In the current study, we investigated the role of miR156/SPL module in flooding tolerance using hormone and transcriptomic profiling combined with physiological responses of *miR156*OE and SPL RNAi alfalfa plants. We also investigated a possible crosstalk between miR156/SPL and ABA-dependent SnRK1 using ABA insensitive (*abi1-2* and *abi5-8*) *Arabidopsis* mutants along with *KIN10* overexpressing and RNAi silenced *Arabidopsis* plants under ABA and low sugar treatments.

## Material and methods

### Genetic material and flooding stress

We performed one field and two greenhouse flooding experiments at the Agriculture and Agri-Food Canada (AAFC) Research Centre in Kentville, Nova Scotia (45°03′46.38″ N, 64°29′10.26″ W) and AAFC Research Center in London, Ontario, respectively. For the field experiment we used wild type (WT), empty-vector alfalfa genotypes, a local cultivar (AC-Caribou), FS-tolerant (AAC-Trueman), *miR156*OE (A8a, *miR156*-A8, A16, A11, A11a, A17), miR156-regulated *SPL6*RNAi (*SPL6*-405, *SPL6*-425, *SPL6*-428), and miR156-regulated *SPL13*RNAi (*SPL13*R-2, *SPL13*R-5, *SPL13*R-6) genotypes as described in supplementary Figs. S1 and 2. The transgenic plants were generated previously in our lab^20,23^. The nucleic acid sequences were obtained from either *M. truncatula* or *M. sativa*, depending on the availability of gene sequences in the public domain. The sequences of miR156 and SPLs are those of *M. sativa*. Where gene sequences were not known for *M. sativa*, we used primers designed based on *M. truncatula* sequences to amplify their homologues *M. sativa* (primers used in qRT-PCR are indicated in supplementary Table S1). Based on field phenotypic responses, forage yield, and transcript analysis, genotypes used in the greenhouse experiment were reduced to WT, AC-Caribou, AAC-Trueman, *SPL13*R-5, *SPL13*R-6 and *miR156*-A8. One-month-old alfalfa plants were subjected to FS in the greenhouse by submerging eight pots of same genotype per tray with water level up to soil surface for two weeks while control plants were well-drained (WD). Greenhouse growth conditions were kept as described in Feyissa et al*.*^24^.

### Measurement of photosynthesis-related parameters

Midday photosynthesis assimilation rates and dark-adapted chlorophyll fluorescence (Fv/Fm) were measured in newly growing unshaded leaves using LI-6400XT portable photosynthesis meter coupled with a Fluorescence System (LI-COR Biosciences, USA). To determine photosynthesis efficiency, photosynthesis assimilation responses across a gradient of CO_2_ levels (0–2000 ppm) (A/Ci) were measured and the maximum rate of rubisco carboxylase activity (*V*_cmax_) and maximum photosynthesis electron transport rate (*J*_max_) were calculated using the R statistical software^31^.

### Hormone profiling

Shoot samples were lyophilized with a Labconco freeze drier system (Kansas, USA) at − 50 °C for three days, and ~ 50 mg of dried samples were used for hormone profiling. Phytohormone analysis was performed at the National Research Council of Canada, Saskatoon, SK on a fee-for-service basis. UPLC/ESI–MS/MS of Waters ACQUITY UPLC system, coupled to a Waters Micromass Quattro Premier XE quadrupole tandem mass spectrometer via a Z-spray interface was used. Quantification of phytohormones was performed using deuterium labeled internal standards^32^.

### Total monomeric anthocyanin and polyphenol determination

Flash-frozen shoots were used to determine monomeric anthocyanin (TMA) employing a pH deferential extraction method as described in Lee et al*.*^33^ and Cheok et al*.*^34^. TMA were expressed as mg cyanidin-3-0-glucoside (CG) equivalent.

### RNA extraction for qRT-PCR and RNAseq

Top shoot tip leaves (~ 50 mg) were collected in Precellys lysing tubes, flash frozen with liquid nitrogen, homogenized by a PowerLyzer 24 and total RNA was extracted using QIAGEN RNeasy Plant mini kit. Total RNA was treated with Ambion TURBO DNA-*free* DNase followed by iScript cDNA synthesis for qRT-PCR using the CFX96 Real-Time PCR detection system (Bio-Rad). Specifically, we mixed 2 µL(200 ng) cDNA, 1 µL forward and reverse gene-specific primers (10 µM each) (Table S1), 5 µL SsoFast EvaGreen Supermixes, and 2 µL of nuclease-free water. PCR amplification was performed using: cDNA denaturation at 95 °C for 30 s followed by 40 cycles of 95 °C for 10 s, 58 °C for 30 s and 72 °C for 30 s followed by a melting curve that ran from 65 to 95 °C with a gradual increment of 0.5 °C per 5 s. All reactions were performed with three technical and four biological replicates. Transcript levels were analysed relative to acetyl-CoA carboxylase (*ACC1*) and *ACTIN* housekeeping genes.

Total RNA quality was checked using a BioRad Bioanalyzer before RNAseq analysis. Stranded mRNA library was prepared with NEBNext followed by sequencing with Illumina NovaSeq6000 with 100 bp fragment pair end reads at Genome Quebec, Montreal as a fee-for-service. Raw RNA sequencing reads can be accessed from the National Center for Biotechnology Information, NCBI, BioProject PRJNA596791.

### RNAseq and pathway analysis

RNAseq data was analyzed according to Trapnell et al*.*^35^ on Biocluster with a Linux interface. *M. truncatula* Mt4.0 V2 (http://www.medicagogenome.org/http://www.medicagogenome.org/downloads) was used as a reference genome due to lack of publicly available alfalfa genome sequence. With the recent tetraploid alfalfa genome report^36^, we searched for the gene IDs of the novel MsSPLs (Table S2). The alfalfa nucleotide sequences of the novel MsSPLs were locally aligned to the newly reported tetraploid alfalfa genome sequence. Gene IDs with 300–680 nucleotide sequences, depending on the MsSPL, and more than 95% identity were considered as a potential candidate from the annotated alfalfa General Feature Format, .gff, file^36^. To identify gene expression patterns and module identification, R-software V3.5.2-based weighted gene co-expression network analysis (WGCNA) was conducted using ‘BiocManager’ package^37^. Pathway analysis was done using MapMan V3.6 (https://mapman.gabipd.org/) with a *M. truncatula* reference genome, and manual incorporation of the differentially expressed genes (DEG) into phenylpropanoid and ABA biosynthesis pathways.

### 5′RACE-based miR156 cleavage site identification

To understand whether miR156 cleaves the newly identified SPLs (*SPL7a, 8* and *13a*) that were silenced under FS, 5′RACE (FirstChoice RLM-RACE) was performed using manufacturer’s—provided two forward primers with designed gene-specific inner (GSI) and outer (GSO) reverse primers (Table S1). *miR156* overexpressing (A17) alfalfa genotype was used in this study. The PCR products were cloned into a pJET1.2 (Clone JET PCR cloning kit), transformed into *E. coli* TOP10 cells by heat shock, and the purified plasmids (GeneJET Plasmid Miniprep kit) were sequenced.

### Investigating protein–protein interaction between SnRK1 and miR156 biogenesis genes

To investigate the role of SnRK1 in miR156 biogenesis, we tested for interaction between SnRK1 (Medtr1g034030.1) and miR156-biogenesis proteins DICER-LIKE (DCL) (Medtr3g102270.2) and finger protein SERRATE (SE) (Medtr8g043980) using the yeast-two-hybrid system (Y2H). We used ProQuest Two-Hybrid System (Invitrogen) system. A detailed description of making constructs and conducting Y2H assay is provided in Table S3.

### Investigating SnRK1 regulation by sugar and ABA in Arabidopsis

To understand whether the expression of SnRK1 is regulated through ABA signalling pathway, low-sugar and ABA, ABA-insensitive Arabidopsis mutants (*abi1-2* and *abi5-8*) were planted in ½ MS media supplemented with T1-control (44 mM sucrose, no ABA), T2 (22 mM sucrose, no ABA), or T3 (22 mM sucrose, 1 µM ABA). Arabidopsis seeds were surface sterilized with 10% bleach for 15 min, rinsed 5 times with sterile water, and plated on the designated treatment plates. Tissue culture plates were stratified at 4 °C for three days and placed at low-light intensity (10 µmol/m^2^s) to reduce photosynthesis-mediated sugar supplement. Treatments were replicated three times placing all genotypes together in each plate per replicate for one week (each genotype contained 50 seedlings). Subsequently, tissues were collected consistently at 2 PM, to rule out variation due to circadian rhythm and an optimized 4-h ABA response, and transcript levels of SnRK1-related genes (Fig. 5) were determined.

### Investigating *SnRK1* regulation by miR156 in alfalfa

To investigate whether the expression of *SnRK1* was upstream of miR156 upon low sugar and ABA treatment in alfalfa, rooted cuttings of *miR156*OE (A17) and WT alfalfa were exposed to either T1-control (15 g/L sucrose, no ABA) or T2 (no sucrose, 100 µM ABA) under FS and kept plants at low-light intensity (25 µmol/m^2^s). One-week-old seedlings were treated with ABA and tissues were collected after 4 h treatment (at 2 PM), and transcript levels of SnRK1-related genes (Fig. 5) were determined.

### Investigating *miR156* expression dependence on SnRK1 in Arabidopsis

Arabidopsis seedlings with increased (*KIN10*-OX1, *KIN10*-OX2) and silenced (*KIN10*RNAi-1, *KIN10*RNAi-2) expression of the catalytic subunit KIN10 were used to understand whether *miR156* expression is dependent on activation of SnRK1 during ABA treatment and sugar starvation. These plants were kindly provided by Dr Filip Rolland, KU, Leuven^13^. The treatment arrangement is similar to the one used for the ‘investigation of SnRK1 expression by sugar and ABA’ except for genotype differences. Subsequently, *miR156* expression levels were determined (Fig. 5).

### Data analysis

Physiological and hormonal data were first checked for normal distribution using a Shapiro–Wilk test in the R-software environment 3.5.2, followed by ANOVA and post hoc Tukey multiple comparison tests. Pair-wise comparison was performed using student t-test when necessary.

## Results

To investigate the role of miR156 in flooding tolerance in alfalfa, we compared *miR156*OE, *SPL6*RNAi, *SPL13*RNAi, WT, and empty-vector plants combined with flooding-sensitive (AC-Caribou) and flooding-tolerant (AAC-Trueman) cultivars. The plants were initially characterized for flooding response using physiological parameters and expression of select flooding-responsive genes (Figs. S1, 3; Table S4). Based on these results, we narrowed the focus to *miR156*-A8, *SPL13*RNAi-5, *SPL13*RNAi-6, AC-Caribou, AAC-Trueman, and WT and repeated the experiment in the greenhouse.

### miR156/SPL module mediates physiological responses of alfalfa during flooding

**Figure 1:**
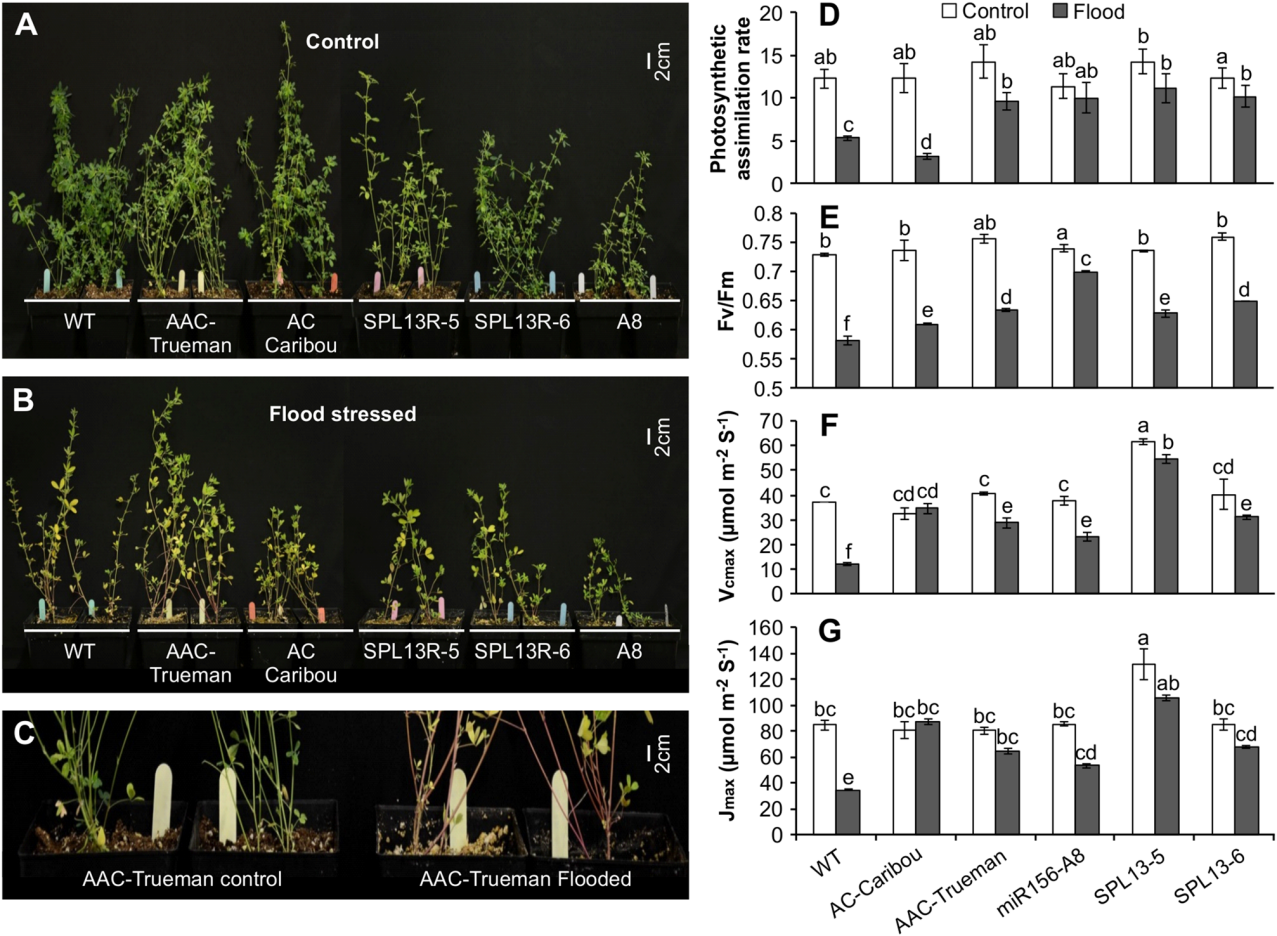
Physiological responses of flood-stressed and control plants. One-month alfalfa plants growing under (**A**) control, and (**B**) flood-stressed conditions, (**C**) stem colour development in AAC-Trueman plants upon flood stress, (**D**) photosynthesis assimilation rate in µ mol CO_2_ m^−2^ leaf area S^−1^, (**E**) chlorophyll fluorescence response, Fv/Fm, (**F**) *V*_cma*x*_, the maximum rate of rubisco carboxylase activity, (**G**) *J*_max_, maximum photosynthesis electron transport rate. Values are sample means ± SE, n = 8 individual plants. A significant difference from ANOVA was followed by Post hoc Tukey multiple comparisons test and indicated with different letters.

Flooding negatively affects photosynthesis and respiration^38^, so we set out to determine whether miR156 regulates this response. Alfalfa plants were subjected to flooding stress (FS) and well-drained conditions (WD) for two weeks (Figs. 1A, S1). Leaf yellowing was observed in all genotypes under FS, but was most severe in AC-Caribou and WT (Fig. 1B) which also had reduced photosynthesis rates than other genotypes (Fig. 1D). The flooding tolerant cultivar, AAC-Trueman, showed increased red colouration in the stems under FS (Fig. 1C). Under WD, there were no significant differences between genotypes in photosynthesis rate (Figs. 1D, S1). Under FS dark-adapted, chlorophyll fluorescence (Fv/Fm), often used as a stress tolerance indicator^39,40^, was maintained at a higher level in AAC-Trueman, *miR156*-A8, and *SPL13*RNAi plants relative to WT (Fig. 1E). To further understand the photosynthesis efficiency, the maximum rubisco carboxylase activity, *V*_*cmax*_, and maximum photosynthesis electron transport, *J*_max_ were determined (Fig. 1F,G). The data showed a similar pattern to the measurements of photosynthesis rate and Fv/Fm ratio observed in Fig. 1D,E. *SPL13*RNAi, *miR156*-A8, and AAC-Trueman maintained a higher level of *V*_cmax_ and *J*_max_ except for AC-Caribou which showed comparable levels despite FS (Fig. 1F,G).

### ABA-metabolites are increased in *miR156OE* alfalfa plants under flooding

**Figure 2:**
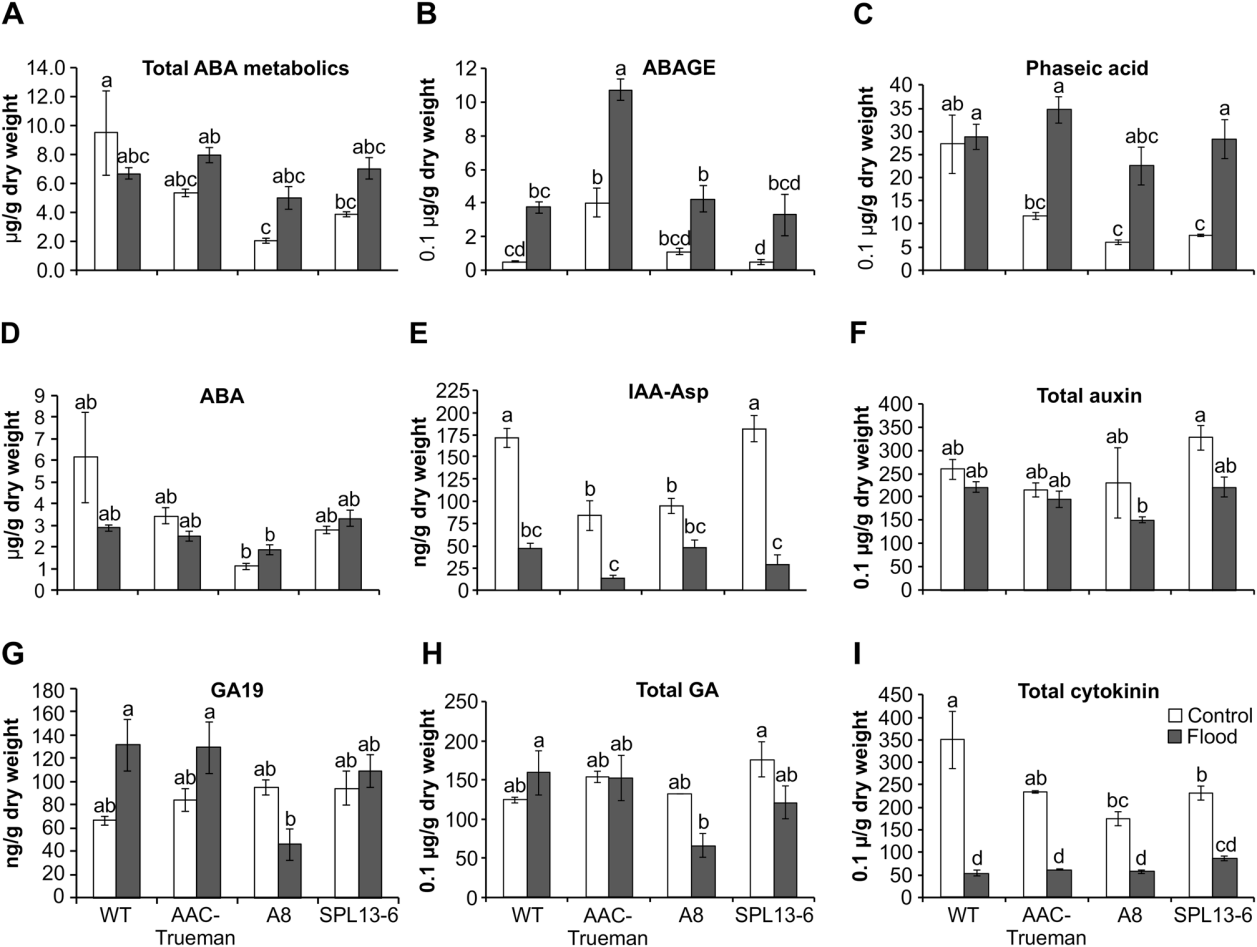
UPLC/ESI–MS/MS-based hormone profiling in flood stressed and control alfalfa genotypes. (**A**) The abundance of total abscisic acid, ABA, metabolites (**B**) abscisic acid glucose ester, ABAGE, (**C**) phaseic acid, PA, (**D**) ABA, (**E**) indole acetic acid-aspartic acid, IAA-Asp, (**F**) total auxin, (**G**) gibberellic acid GA19, (**H**) total gibberellic acid GA, and (**I**) total cytokinin in ng per gram leaf dry weight. Total ABA metabolites in ‘**A**’ comprises ABA, Dihydrophaseic acid (DPA), ABAGE (Abscisic acid glucose ester), Phaseic acid (PA), 7′-Hydroxy-abscisic acid (7′OH-ABA), neo-Phaseic acid (neo-PA), and trans-Abscisic acid (t-ABA). Total auxin in ‘**F**’ comprises Indole-3-acetic acid (IAA) and N-(Indole-3-yl-acetyl)-aspartic acid (IAA-Asp). Total gibberellic acid in ‘H’ comprises gibberellin 8, 9, 19, 20, 24, 29, 34, 44, 51, and 53. Total cytokinin in ‘**I**’ comprises (trans) Zeatin-O-glucoside (t-ZOG), (cis) Zeatin-O-glucoside (c-ZOG), (cis) Zeatin riboside (c-ZR), Dihydrozeatin riboside (dhZR), Isopentenyladenosine (iPR), Values are sample means ± SE, n = 3 individual plants as biological replicates. A significant difference from ANOVA was followed by Post hoc Tukey multiple comparisons test and indicated with different letters.

Plant hormones, such as ethylene, mediate rice and Arabidopsis responses to FS by inducing *SUB1A* and ABA^5,7,41,42^. The role of ABA-catabolite, phaseic acid (PA), was also demonstrated in Arabidopsis response to drought, where it enhances ABA-like PYRABACTIN RESISTANCE/PYR-LIKE/REGULATORY COMPONENTS OF ABA RECEPTORS-based (PYR/PYL/RCAR) signal perception and PA-specific responses^41,43^. We investigated the hormone profiles of FS- and WD-treated alfalfa genotypes to understand hormonal changes. Total ABA metabolites, which comprises of ABA, PA, ABA-glucose ester (ABAGE), and another four ABA derivatives, were increased in FS plants relative to their WD by 1.5- (AAC-Trueman) to 2.5 –fold (*miR156*-A8), while a 30% reduction was observed in WT (Fig. 2A). Specifically, ABA-metabolites from glucosyl esterification and oxidation reactions, ABAGE and PA, respectively, contributed a significant portion of the total ABA metabolite abundance under FS, second only to ABA (Fig. 2B–D). For instance, while PA concentration was not changed between FS and WD WT, an average fold-increase of 2.95- (AAC-Trueman) to 3.75 (SPL13RNAi-6) was observed in the tolerant genotypes (Fig. 2C). The results suggest an ABA-dependent response mechanism in flood tolerant alfalfa plants (Fig. 2).

Developing adventitious roots and shoot elongation are regulated by auxin, ethylene, and gibberellin, and are used as a flood escape mechanisms by some plants^44^. There was a significant decrease in IAA-Asp and total gibberellin levels in response to FS (Fig. 2E–H, Table S5). Variable reductions in vegetative growth (Fig. 1A) were suggested to correlate with reduced cytokinin levels^45^, a correlation observed in all alfalfa genotypes in this study (Fig. 2I). The detailed hormone profile is provided in Table S6.

### miR156 regulates secondary metabolism pathways to improve flooding tolerance

To investigate whether the miR156 gene regulatory network is involved in flooding tolerance, determined RNA expression levels of the flooding responsive *SNORKEL1*^8^, as well as *miR156* and miR156-regulated *SPL4* and *SPL13* genes^20,30^ in *miR156*OE, *SPL13*RNAi, WT, AC-Caribou and AAC-Trueman plants (Fig. S3). Transcript levels of *SNORKEL1* and *miR156* were increased in *miR156*OE plants, whereas those of *SPL4* and *SPL13* were decreased upon FS (Fig. S3). Based on these results, we decided to investigate changes in global transcriptome using *miR156*-A8, *SPL13*RNAi-6, AC-Caribou, AAC-Trueman, and WT alfalfa.

**Figure 3:**
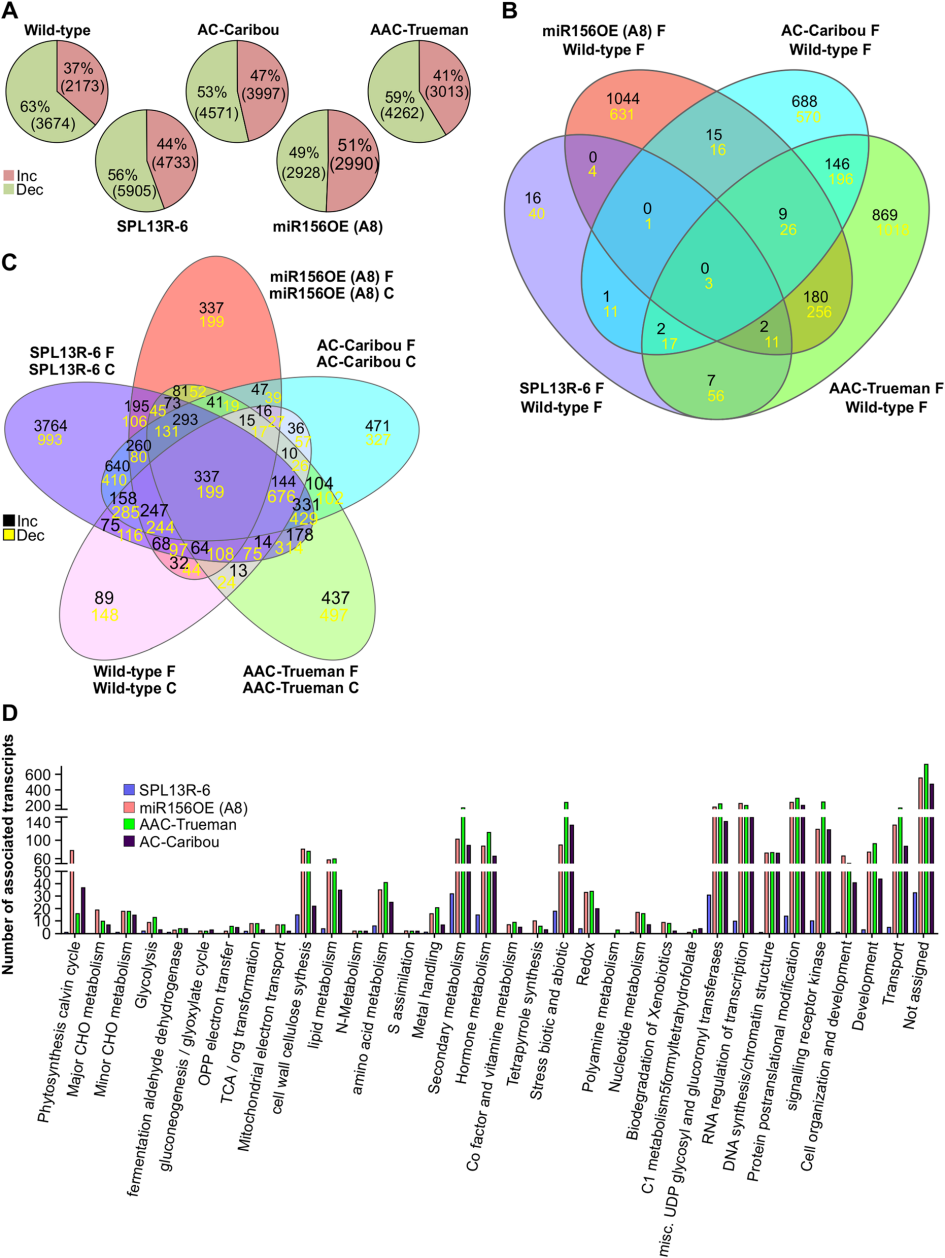
Differentially expressed genes and their associated function upon flood stress in alfalfa. (**A**) Differentially expressed genes, DEG, in each genotype comparing flooding and well-drained control alfalfa plants, (**B**) Venn diagram illustrating commonality and uniqueness in DEG obtained by comparing flood-stressed WT to AC-Caribou, AAC-Trueman, *SPL13*RNAi-6 and *miR156*-A8 genotypes independently upon flooding, (**C**) Venn diagram illustrating commonly shared and unique DEG obtained by comparing flood-stressed and their respective well-drained control counterparts plants of each genotypes, (**D**) functional distribution of DEG generated by comparing flood-stressed WT to AC-Caribou, AAC-Trueman, *SPL13*RNAi-6 and *miR156*-A8 genotypes. Upper and lower panel numbers in ‘**B**’ and ‘**C**’ indicate increased and decreased DEG, respectively, compared to WT flood stress or the same genotype under control conditions respectively. Novaseq 6000-based RNAseq analysis was performed for three biological replicates for each treatment condition and genotype. Venn diagram was constructed using an online tool http://www.interactivenn.net/ from DEG.

Transcript profiles comparing FS and WD revealed that over 60% of DEG were decreased in FS WT, whereas the differences between upregulated and downregulated genes were minimal (51% increased and 49% decreased) in FS *miR156*-A8 (Fig. 3A). But AC-Caribou, SPL13R-6, and AAC-Trueman reduced 53, 56 and 59% of DEG, respectively. To further identify genes contributing to alfalfa flooding tolerance, we compared DEG from FS WT to those from AC-Caribou, AAC-Trueman, *SPL13*RNAi, and *miR156*-A8 FS (Fig. 3B). Genotype-specific and commonly shared DEG were observed. Interestingly, two up-regulated and 11 down-regulated genes were commonly shared by the flood-tolerant AAC-Trueman, *miR156*-A8 and *SPL13*RNAi-6 genotypes. The two up-regulated transcripts code for Gly-Asp-Ser-Leu (GDSL)-like lipase/acyl hydrolase (Medtr8g087870) and a reticuline oxidase-like protein (Medtr2g031560). On the other hand, five of the 11 commonly down-regulated transcripts under FS code for carbonic anhydrase (Medtr0219s0070), galactinol-raffinose galactosyltransferase (Medtr7g091880), AP2 domain class transcription factor (Medtr3g098580), PAR1 protein (Medtr1g101120), and sieve element occlusion protein (Medtr1g074990).

GDSL-like lipase/acyl hydrolase contributes to plant response to abiotic and biotic stresses and to seed development by modulating lipid metabolism^46–49^. A comparison of WT with the other genotypes under FS identified two other major GDSL-related genes, including GDSL-like lipase/acyl hydrolase and Pmr5/Cas1p GDSL/SGNH-like acyl-esterase (Fig. S4A). Of these genes, one of three in *SPL13*RNAi-6, 29 of 33 in *miR156*-A8, two of six in AC-Caribou and nine of 14 in AAC-Trueman were increased under flooding compared to FS WT (Fig. S4A). The second commonly increased transcript in all three genotypes (*miR156*-A8, *SPL13*RNAi-6 and AAC-Trueman) codes for the reticuline oxidase-like protein which binds flavin adenine dinucleotide (FAD) that serves as hydrogen acceptor in a bi-covalent manner, and possesses an oxidoreductase activity in (S)-scoulerine biosynthesis^50^. The (S)-scoulerine alkaloid biosynthesis pathway is downstream of tyrosine required for the biosynthesis of important alkaloids such as berberine^51^. Transcripts of this gene were increased 1.7-fold in FS AAC-Trueman relative to its WD while other genotypes (WT, AC-Caribou, *miR156*-A8, and *SPL13R*-6) showed a reduced 0.22 to 0.53-fold change compared to their respective WD (Fig. S4B). Despite this reduction, reticuline oxidase-like protein transcripts were increased 1.6 (*miR156*-A8) to sixfold higher (AAC-Trueman) relative to FS WT (Fig. S4B).

**Figure 4:**
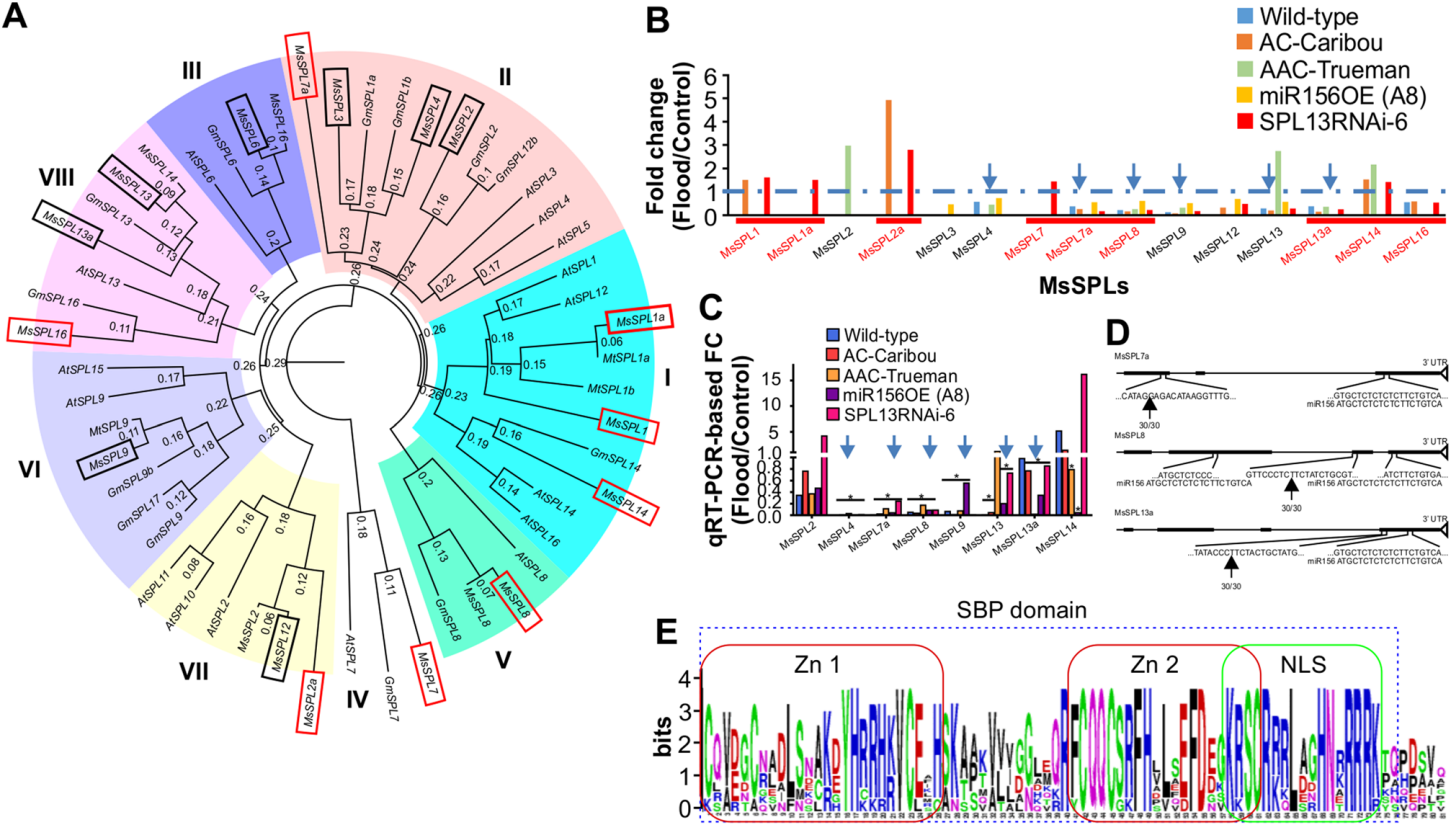
Phylogenetic analysis and identification of new SPLs in alfalfa. (**A**) Amino acid coding sequence-based phylogenetic analysis of SPLs, (**B**) Differentially expressed SPLs between control and flood-stressed alfalfa plants, (**C**) Validation of the consistently regulated SPLs from RNAseq using qRT-PCR, (**D**) The conserved SQUAMOSA PROMOTER-BINDING PROTEIN, SBP, domain in the newly identified SPLs containing two zinc-finger binding domains (Zn 1 and 2) and nuclear localization signal (NLS), (**E**) Complementarity of SPL7a, 8 and 13a to that of matured miR156 sequence along with 5′ RLM RACE determined cleavage sites. SPLs boxed with red line in ‘**A**’ and underlined in ‘**B**’ are newly identified SPLs while boxed with black lines were previously identified (Aung et al., 2015b; Gao et al., 2016). Arrows in ‘**B**’ and ‘**C**’ indicates commonly reduced SPLs during FS. Phylogenetic analysis is done using clustal omega online tool (https://www.ebi.ac.uk/Tools/msa/clustalo/) followed by FigTree v.1.4.2 free software. The ‘*’ in ‘**C**’ indicates significance level at *p* < 0.05 when compared between control and flooding transcript levels. In silico amino acid sequence in ‘**D**’ is analysed using http://weblogo.berkeley.edu/logo.cgi. In ‘**D**’ zinc-finger binding domains (Zn 1 and 2) and nuclear localization signals (NLS) are indicated with red and green boxes, respectively. Arrows in ‘**D**’ indicates cleavage site by miR156.

To understand transcript plasticity of genotypes in response to flooding, we compared the transcript profiles of WD and FS plants of each genotype and compared their similarity. We identified 1071 upregulated and 1624 downregulated DEG shared by all genotypes (Fig. 3C). In addition, 89, 3764, 337, 471, and 437 upregulated and 148, 993, 199, 327, and 497 downregulated genotype-specific DEG were detected in WT, *SPL13*RNAi-6, *miR156*-A8, AC-Caribou, and AAC-Trueman genotypes, respectively (Fig. 3C). Among the transcripts differentially expressed in all genotypes upon FS, ABA biosynthesis, *SnRK1*, and phenylpropanoid pathway genes were upregulated (Fig. S4C,D, Table S7, 8) while SPLs had lower transcript levels (Fig. 4B). The number and distribution of different molecular function-associated DEG between FS WT and all other genotypes code primarily for protein post-translational modification, miscellaneous UDP glucosyl and glucoronyl transferases, RNA regulation of transcription, signalling receptor kinase, biotic and abiotic stress, transport, secondary metabolism, plant hormone metabolism, development and cell wall cellulose synthase (Fig. 3D).

### Novel SPLs regulated by miR156 in alfalfa

Previous reports revealed seven *SPL*s (*SPL2, SPL3, SPL4, SPL6, SPL9, SPL12*, and *SPL13*) were regulated by miR156 in alfalfa^20,30^. Our observation that *miR156* expression was enhanced under FS (Fig. S3D) prompted us to investigate if *miR156*-regulated *SPL*s contribute to alfalfa’s response to FS. RNAseq analysis followed by transcript annotation of five alfalfa genotypes exposed to FS showed that 15 *SPL*s were affected by FS relative to their WD counterparts (Fig. 4 A,B). We retrieved amino acid coding nucleotide sequences from a database (http://www.medicagogenome.org/) for the new SPLs along with previously used sequences^30^ for phylogenetic analysis for *Medicago truncatula*, *Glycine max*, and *Arabidopsis thaliana* (Table S9). Besides, the previously known seven SPLs, nine new SPLs (SPL1, SPL1a, SPL2a, SPL7, SPL7a, SPL8, SPL13a, SPL14 and SPL16) were identified in this study from database search and amino acid sequence alignment (Fig. 4A). The naming of the new SPLs is based on the closely related known SPLs from the phylogenetic tree in the clade. Phylogenetic analysis grouped the SPLs into eight clades that have over 75% coding sequence similarity. Clade I (SPL1, SPL1a, SPL14), clade II (SPL2, SPL3, SPL4, SPL7a), clade III (SPL6), clade IV (SPL7), clade V (SPL8), clade VI (SPL9), clade VII (SPL2a, SPL12), and clade VIII (SPL13, SPL13a, SPL16) (Fig. 4A). Of these *SPLs, SPL4, SPL7a, SPL8, SPL9, SPL13, SPL13a* were downregulated under FS compared to WD in all genotypes, *SPL3* was downregulated only in *miR156*-A8, while *SPL12* and *SPL16* were not consistently downregulated in all the genotypes (Fig. 4B). The transcriptomic data were validated by qRT-PCR (Fig. 4C) by amplifying each SPL (Table S1).

The newly identified SPLs were further analyzed for their conserved SBP domain, nuclear localization signal, and presence of miR156 binding nucleotide sequences. In silico amino acid sequence analysis (http://weblogo.berkeley.edu/logo.cgi) of the newly identified SPLs revealed they all contained the conserved SBP domain (Fig. 4E). Using an amino acid sequence-based online tool (http://nls-mapper.iab.keio.ac.jp/cgi-bin/NLS_Mapper_form.cgi), nuclear localization signals were also detected in the newly identified SPLs (Fig. S4).

To understand whether the newly identified SPLs are regulated by miR156, we first checked for complementarity to mature miR156 ‘ATGCTCTCTCTCTTCTGTCA’ in a 5′ to 3′ orientation and found different proportions of miR156 matching sequence in SPLs ranging from 13/20 in *SPL1* and *SPL7* to 19/20 in *SPL2a*, *SPL7a* and *SPL13a* (Fig. S5). Of the different proportions of miR156 matching sequence to *SPLs, SPL7a, SPL8* and *SPL13a* possessed 19/20, 18/20 (in two fragments) and 19/20 nucleotide matches to miR156, respectively (Fig. 4D). miR156 cleavage sites in transcripts of *SPL*s silenced under flooding (*SPL7a, SPL8* and *SPL13a*) were determined using 5′-RACE. The sequencing results revealed *SPL7a, SPL8 and SPL13a* were cleaved by miR156 upstream of the complementary target sequence (Fig. 4D). These results revealed that miR156 downregulates *SPL4, SPL7a, SPL8, SPL9, SPL13* and *SPL13a* in alfalfa in response to FS.

### Flooding enhances *SnRK1* expression in an ABA-dependent manner

The protein kinase SnRK1 was reported to be elevated in *Arabidopsis* that undergo a REM response due to abiotic stress^13,14^. We observed an upregulation of *SnRK1* (Medtr1g034030) and its regulatory β subunit (Medtr5g098510 and Medtr2g095290) in alfalfa under FS (Fig. S4C). The catalytic α subunit *KIN11* (Medtr6g048250 and Medtr6g012990) was also increased consistent with *SnRK1* expression in two of the genotypes (AC-Caribou and *SPL13*RNAi-6) (Fig. S4C). Considering the elevated levels of ABA-metabolites in AAC-Trueman, *miR156*-A8 and *SPL13*RNAi-6 (Fig. 2) upon FS, we investigated the ABA signaling pathway and ABA-responsive elements in these plants. The ABA signaling PYL9/PYR1 receptors as well as ABI2 and ABA-responsive element ABRE were upregulated, whereas ABI1 was downregulated under FS (Figs. 2, S4D). Given an enhanced level of SnRK1 and ABA and findings from the literature showing SnRK1’s central role in sugar and ABA signalling ^18,52^, we investigated whether the expression of SnRK1 is ABA-dependent.

**Figure 5:**
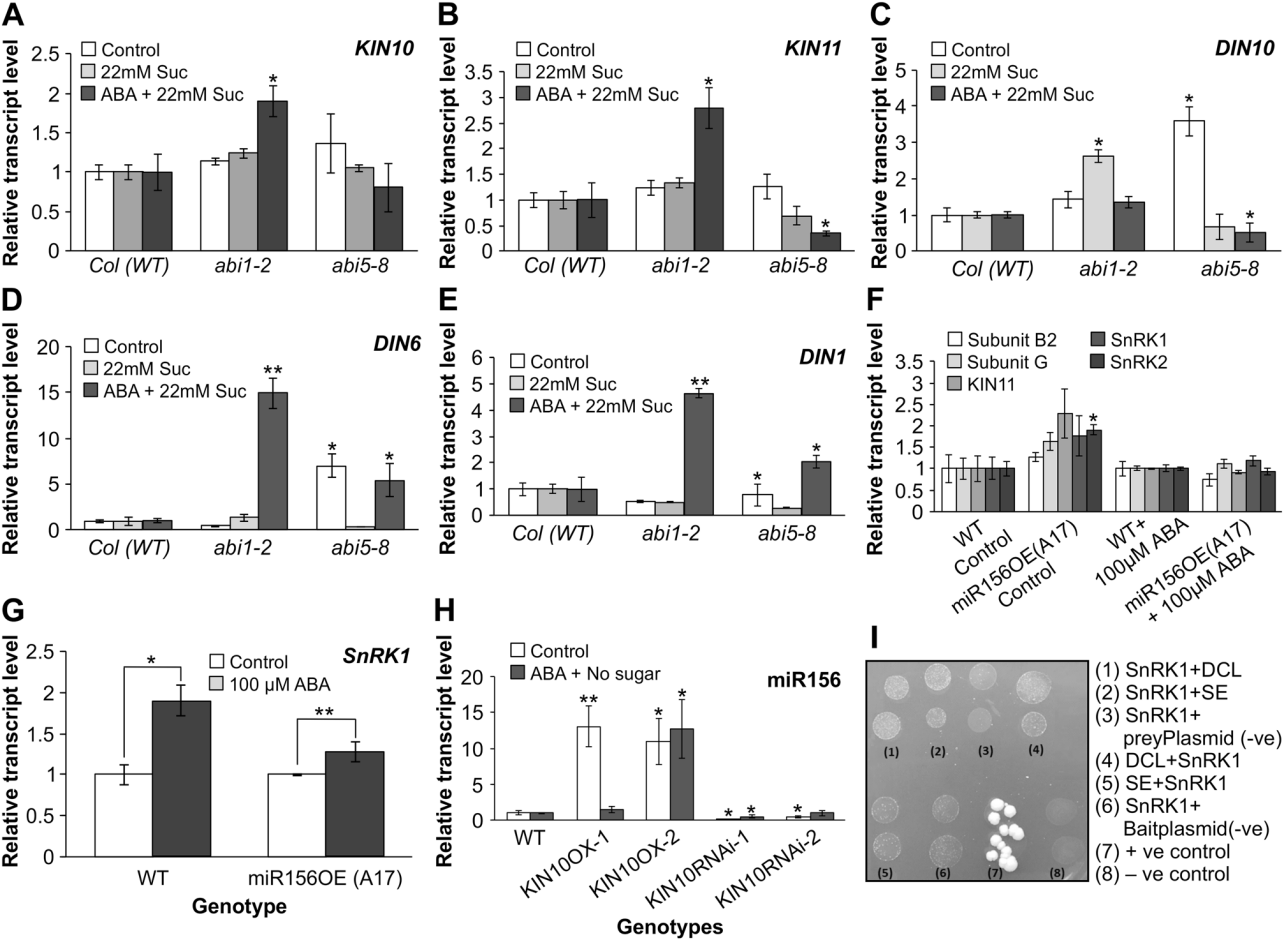
ABA-dependent expression of *SnRK1* may regulate *miR156*. SnRK1 catalytic subunits (**A**) *KIN10*, (**B**) *KIN11,* relative transcript levels of *DARK INDUCED, DIN,* (**C**) *DIN10*, (**D**) *DIN6*, (**E**) *DIN1* genes in *Arabidopsis*, (**F**) relative transcript levels of *SnRK1* and *SnRK2* along with *SnRK1* regulatory and catalytic subunits in response to 100 µM ABA treatment, (**G**) transcript levels of *SnRK1* upon 100 µM ABA treatment relative to their counterpart control alfalfa plants, (**H**) relative transcript levels of *miR156* in KIN10 overexpressing and RNAi silenced Arabidopsis plants, (**I**) Yeast-two-hybrid (Y2H) assay for the interaction between SnRK1 and miR156 biogenesis genes. n = 50 biological replicates of Arabidopsis in ‘**A**’ to ‘**E**’ and ‘**H**’ while three alfalfa plants in ‘**F**’ and ‘**G**’. Arabidopsis specific three housekeeping genes (elongation factor-alpha, tubulin, and actin) are used for relative transcript analysis relative to WT in ‘**A**’ to ‘**E**’ and ‘**H**’ while alfalfa specific elongation factor, actin and ubiquitin10 are used in ‘**F**’ and ‘**G**’. Values are means with ± SE. In ‘**I**’, protein-coding whole sequences of the genes were amplified using gene-specific primers with the addition of ‘CACC’ in the forward primer, for directional cloning (Table S1). The interaction between the pEXP32/Krev1 (rat Krev1) with pEXP22/RalGDS-wt (ras association domain of RalGDS) is used as positive control while for the negative control pEXP32/Krev1 with the mutated ras association domain of RalGDS, pEXP22/RalGDS-m2, was used according to the user’s manual.

Noticing the upregulation of *SnRK1*, ABA-signaling elements (Table S7, Fig. S4D) and ABA (Fig. 2) in alfalfa under FS, we determined the transcript levels of *KIN10*, *KIN11* and *DARK INDUCED* genes (*DIN*). This was done in ABA insensitive *Arabidopsis* mutants (*abi1-2,* and *abi5-8*)^53,54^, due to the lack of similar mutants in alfalfa. The ABA-insensitive mutant and WT *Arabidopsis* seedlings were treated with either 22 mM sucrose and 1 µM ABA or 22 mM sucrose alone under low light growth conditions. Expression level of *KIN11* was reduced in *abi5-8* while both *KIN10* and *KIN11* were increased in *abi1-2* plants during ABA treatment, where only one of the calcium and protein binding elements (ABI1) is silenced (Fig. 5A,B). We also determined whether the expression of dark-induced and multiple stress responsive *DIN* genes (*DIN1*, *DIN6* and *DIN10*)^13^ were affected by ABA treatment. We found that the expression of *DIN10* was increased with reduced sugar level in *abi1-2* mutants while reduced in *abi5-8* mutants with ABA application (Fig. 5C). This result shows *DIN10* expression is ABA-dependent and regulated by ABI5 (Fig. 5C). On the other hand, RNA transcript levels of *DIN1* and *DIN6* were increased in the presence of ABA despite a reduced level of *ABI1* and *ABI5* (Fig. 5D,E).

To determine whether the transcription of *SnRK1* in alfalfa is affected by miR156 expression level, we exposed one-month old rooted cuttings of alfalfa plants (*miR156*OE and WT) to FS for one week followed by treatment with of 100 µM ABA for 4 h. Then the transcript levels of *SnRK1* and its catalytic α (*KIN10* and *KIN11*) and regulatory (β and γ) subunits, and SnRK2 were analysed. Under control conditions, only *SnRK2* was significantly increased in *miR156*OE plants whereas the other regulatory and catalytic subunits were not affected relative to WT (Fig. 5F). Interestingly, under 100 µM ABA treatment, the expression levels of all the catalytic, regulatory subunits, *SnRK1* and *SnRK2* were not significantly different from those of WT (Fig. 5F). Both WT and *miR56OE* genotypes had higher *SnRK1* expression under 100 µM ABA treatment compared to their counter part controls indicating the ABA-dependent SnRK1 activation is not dependent on miR156 (Fig. 5G).

### Does SnRK1 regulate *miR156*?

To understand whether SnRK1 regulates *miR156* during flooding response, we used *Arabidopsis* plants with altered *KIN10* expression (*KIN10*-OX-1, *KIN10*-OX-2, *KIN10*RNAi-1*, KIN10*RNAi-2)^13^ treated with 3 µM ABA, and determined *miR156* transcript level. The transcript level of *miR156* was significantly higher in *KIN10-OX* while *KIN10*RNAi plants showed a comparable (*KIN10*RNAi-2) or lower (*KIN10*RNAi-1) levels relative to WT (Fig. 5H). Interestingly, under ABA treatment, *miR156* expression level stayed significantly higher at least in one *KIN10*-OX (*KIN10*-OX-2) but lower in *KIN10*RNAi plants when compared to WT (Fig. 5H).

*DICER-LIKE 1* (*DCL1*), *SERRATE* (*SE*) and *HYPONASTIC LEAVES* (*HYL1*) are critical for the biogenesis of microRNAs^55^. Our observation that increased *KIN10* expression was correlated with upregulation of *miR156* (Fig. 5H) while overexpression of *miR156* did not affect *KIN10* (Fig. 5F) prompted us to investigate whether SnRK1 regulates miR156 biogenesis in alfalfa. A recent report showed that inactivation of SnRK2 kinases causing reduced phosphorylation of SE and HYL1 led to a decrease in microRNA biogenesis under stress conditions^56^. We used a yeast-two-hybrid assay to investigate the in vivo pairwise protein–protein interactions between SnRK1 with DCL1 and SE. Apparently, no protein–protein interaction was detected between SnRK1 and either DCL1 or SE (Fig. 5I).

### miR156/SPL module enhances flooding adaptive mechanisms

**Figure 6:**
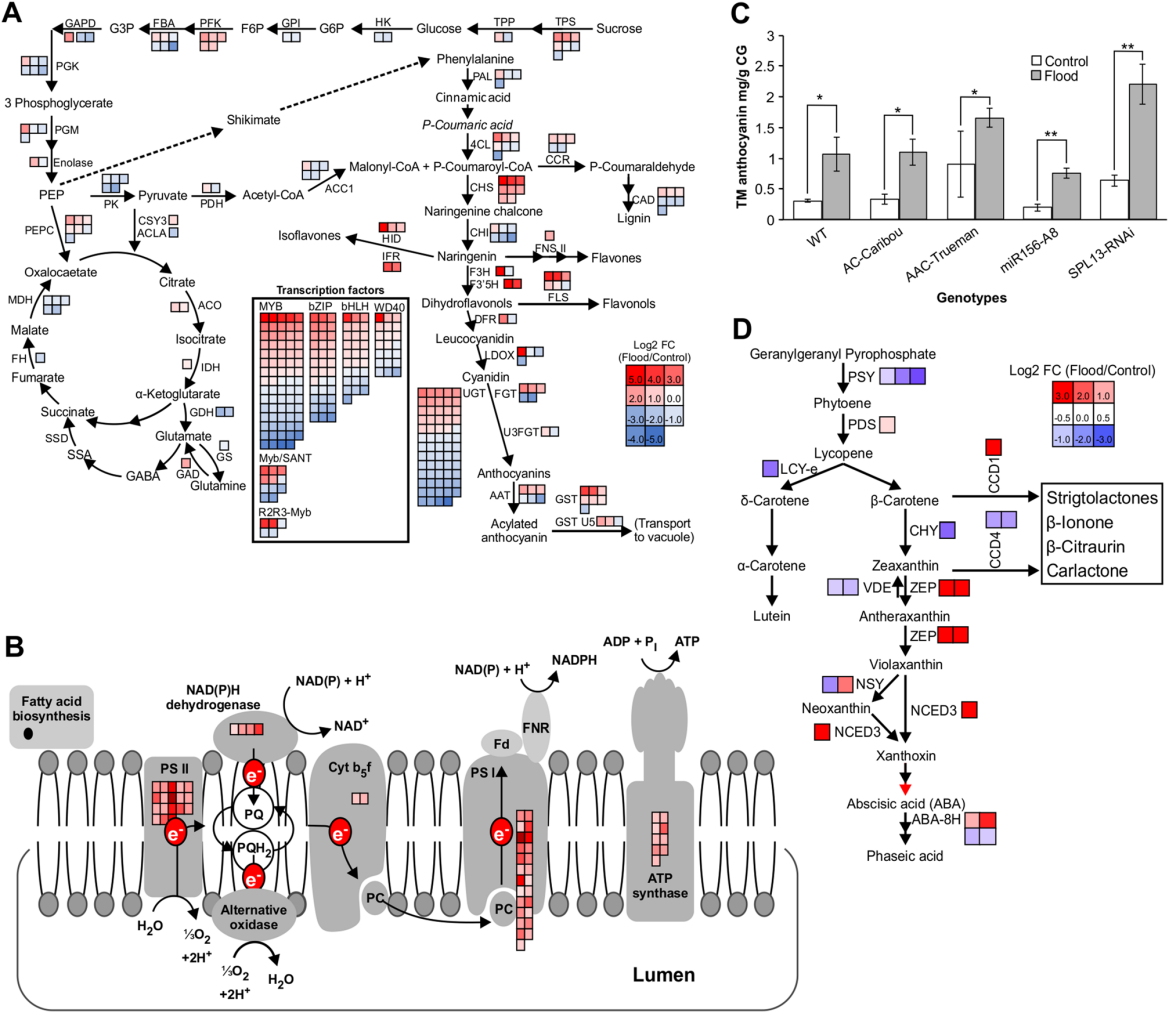
miR156-based regulation of SPL13 enhances photosynthesis, phenylpropanoid, and ABA biosynthesis in response to flooding. (**A**) Illustration of reduced Glycolysis and TCA cycle with an enhanced phenylpropanoid pathway in flood-stressed *SPL13*RNAi plants based on differentially expressed genes, (**B**) MapMan-based pathway analysis illustrating Photosystem I and II associated increased transcript abundance along with electron transport chain in *miR156*-A8 plants compared to WT plants during FS, (**C**) Total monomeric anthocyanin in Gallic acid equivalents, (**D**) enrichment of ABA biosynthesis in *SPL13*RNAi plants upon flooding. n = 3 biological replicates. Pathways in ‘**A**’ and ‘**C**’ were manually generated based on the KEGG pathway database (https://www.genome.jp/kegg/pathway.html)^70^ with permission and previous reports on glycolysis, phenylpropanoid, and carotenoid biosynthesis pathway. DEG corresponding to construct the phenylpropanoid and glycolysis pathway in ‘**A**’ are presented in Tables S6 and S7, respectively. Values in ‘**C**’ are mean with ± SE.

The transcriptomic profile of *SPL13*RNAi plants shows an increase in genes coding for trehalose-6-phosphate synthase (*TPS*) and trehalose-6-phosphate phosphatase (*TPP*) (Fig. 6A). The reduction in photosynthesis assimilation rate after two weeks of FS (Figs. 1C, 6B, Table S10) may cause lower level of fructose and glucose, which in flooding tolerant plants is compensated for by increased levels of TPS and TPP to enhance sucrose hydrolysis. Such REM is sensed by SnRK1 in *Arabidopsis* to coordinate metabolic responses^57^. Likewise, levels of *SnRK1* along with its catalytic and regulatory subunits are increased in alfalfa during FS (Figs. 5G, S3C). Moreover, induction of the phenylpropanoid pathway is observed in flood-tolerant *SPL13*RNAi plants to scavenge ROS (Fig. 6C, Table S8). We previously reported an interplay between miR156-SPL13 and WD40-1F-DFR in alfalfa to enhance biosynthesis of abiotic stress-alleviating anthocyanin through the phenylpropanoid pathway^24^. Consistent with these findings, we detected an increase in total anthocyanin monomers under FS (Fig. 6C).

In the current study, we detected an increase in the level of ABA metabolites (PA, ABAGE) during FS in flood-tolerant alfalfa genotypes (AAC-Trueman, *miR156*-A8 and *SPL13*RNAi-6) (Fig. 2). The increase in accumulation of ABA metabolites under FS is regulated at the transcript level based on results of global transcriptomic-derived pathway analysis in *SPL13*RNAi plants (Fig. 6D) and other flood-tolerant genotypes (Table S7).

## Discussion

### miR156 regulates physiological processes during flooding stress

Plants survive abiotic stress by employing adaptation or escape strategies (individually or in combination) to maintain essential physiological processes required for plant growth and development. Altering root architecture (among others) has been reported for plants adapted to growth under flooding^58^, and is a mechanism by which some plants can regulate response to flooding stress. For example, in *Alternanthera philoxeroides* flooding induces the formation of adventitious roots that help the plant absorb oxygen from ambient water, thus alleviating the deleterious effects of this stress^59^. In alfalfa, plants overexpressiong miR156E had enhanced drought stress, and showed thicker, denser and more adventitious roots compared to WT control^23^. Our results showed that *miR156*OE alfalfa plants maintained an active photosynthesis activity during FS as expressed in photosynthesis electron transport and maximum rate of carboxylase activity. This came with a higher dark-adapted chlorophyll florescence, Fv/Fm, and ultimately a stable photosynthesis assimilation rate. Interestingly, *SPL13*RNAi and the flooding-tolerant genotype, AAC-Trueman, phenocopied the response of *miR156*-A8. The reduced levels of Fv/Fm and photosynthesis assimilation rate, but with no effect on *V*_cmax_ and *J*_max_ in AC-Caribou under FS, suggests a viable Ribulose-1,5-bisphosphate carboxylase/oxygenase (Rubisco) enzyme but its activity may be hindered by the scarcity of CO_2_ under stress. Understanding the maintenance of *V*_cmax_ and *J*_max_ in AC-Caribou under FS would require studies on the Rubisco enzymatic activity.

### Phaseic acid-dependent regulation of flooding tolerance in alfalfa

We investigated the role of phytohormones in alfalfa’s response to FS and found an increased level of ABA-metabolites under FS in tolerant genotypes (AAC-Trueman, *miR156*-A8 and *SPL13*RNAi-6), whereas WT had reduced levels. Of the different ABA-metabolites involved in signalling, phaseic acid (PA), was recently investigated for its role in plant adaptive plasticity^41^. The report showed how PA could have an ABA-like effect, and PA-specific responses during FS. In our study, we also detected a significant increase in the amount of PA in alfalfa genotypes of *SPL13*RNAi, *miR156*OE and AAC-Trueman under FS relative to WD counterparts.

Gibberellic acid, GA, is involved in internode elongation resulting in taller plants benefiting underwater-submerged plants^60^. The increase in plant height under the influence of GA comes with the cost of energy dissipation that competes with other physiological processes. In this regard, WT enhanced the GA abundance and increased plant height while *miR156*OE and *SPL13*RNAi reduced their GA levels under FS. This follows the typical *miR156*OE alfalfa phenotype with increased number of branches but reduced shoot height^19^. It seems that *miR156*OE and *SPL13*RNAi plants channel the assimilated carbon into the biosynthesis of stress mitigating metabolites, such as anthocyanin. Increasing plant height might be an important strategy in fully underwater-submerged plants facilitating gas exchange, but our experiment involved FS only up to the soil surface. It remains to be investigated whether the hormone profile will change in these alfalfa genotypes if plants were completely submerged under water.

### Genotype-specific enhancement of secondary metabolism and photosynthesis

Under FS, plants have a reduced availability of O_2_ and CO_2_, which are important for protein biosynthesis and energy production^10,12^. On the other hand, gene expression followed by translation is an energy consuming process^61^. In our study, the flood-tolerant *miR156*-A8 plants maintained a comparative level of DEG between FS and WD while others showed mainly reduction under FS.

Besides the proportion of DEG affected by FS, identifying FS-responsive genes and their association/network via pathway enrichment analysis is important to understand FS-regulation mechanism. The increased expression levels of photosynthesis-related genes in *SPL13*RNAi and flood-tolerant AAC-Trueman genotypes during FS indicates the maintenance of physiological processes. This is in line with the observed maintenance of *V*_cmax_ and *J*_max_ that ultimately resulted in a relatively higher photosynthesis assimilation rate during FS. FS induces the production of ROS (as part of the photosynthesis process) resulting in a negative feed-back that damages cellular integrity and enzymatic reactions^62^. Flood-tolerant plants, on the other hand, retain ROS levels to maintain normal physiological processes. Plant use of secondary metabolites to scavenge ROS is well documented^63,64^. The observed enrichment of secondary metabolism, specifically those of the phenylpropanoid pathway, suggests that flood-tolerant genotypes of alfalfa used a similar strategy to mitigate FS. Pathway enrichment analysis showed enhancement of cell wall metabolism, ROS-scavenging ascorbates and glutathione associated transcripts. This agrees with the transcript profile of *SPL13*RNAi plants, where *Flavanone 3 hydroxylase* (*F3H*)*, Flavanone 3′5′hydroxylase* (*F3′5H*) and *Dihydroflavonol 4-reductase* (*DFR*) were upregulated in accordance with the enhanced level of total monomeric anthocyanin during FS. Analyzing the network of global transcriptome changes to metabolome under FS will provide a better understanding of alfalfa’s response to FS.

### Identification of novel SPLs in alfalfa

A previous study identified seven SPLs (SPL2, SPL3, SPL4, SPL6, SPL9, SPL12 and SPL13) that are regulated by miR156 in alfalfa^20,30^. Here, RNAseq followed by gene ontology analysis revealed 16 SPLs in alfalfa, of which nine are novel sequences. SPLs are transcription factors that regulate the expression of downstream genes having diverse functions in plants^65^. Identifying new SPLs should shed light on novel molecular factors that control various aspects of alfalfa growth and development. The nine newly identified SPLs (SPL1, SPL1a, SPL2a, SPL7, SPL7a, SPL8, SPL13a, SPL14 and SPL16) can now be subjected to functional characterization in alfalfa.

Based on their sequence similarities, the previously identified seven SPLs and the nine SPLs reported in this study were organized into eight clades with those of *Medicago truncatula, Glycine max* and *Arabidopsis thaliana* in the phylogenetic tree. The newly identified SPLs are distributed into six out of the eight clades. Understanding the clade distribution of the newly identified SPLs along with the other SPLs from different plant species could provide information regarding their putative function considering their similarity in coding sequences. For example, clade I assigned SPLs, such as AtSPL1, are important for thermo-tolerance at the reproductive stage, redundantly with AtSPL12^66^. The newly identified SPL1, SPL1a and SPL14 may also have a role in thermos-tolerance, but this should be validated using gene silencing and overexpressing alfalfa plants under heat stress. Similarly, SPL7a may have similar function to that of SPL2, SPL3, SPL4, in alfalfa and AtSPL3, AtSPL4 and AtSPL5 in Arabidopsis. In line with this, SPLs from clade II (SPL7a and SPL4) were silenced under FS in the current study.

### miR156-regulated SPLs are involved in alfalfa flooding response

Identification of SPLs affected by FS is important to understand the role of miR156/SPL gene network in alfalfa’s response to this stress. In this study, we identified three new SPLs (SPL7a, SPL8 and SPL13a) and five of the previously known (SPL3, SPL4, SPL9, SPL12, SPL13) to be downregulated in *miR156*-A8 plants exposed to flooding. SPL13 is one of the six SPLs (SPL4, SPL7a, SPL8, SPL9, SPL13, SPL13a) that were downregulated in all alfalfa genotypes upon FS, and is also silenced by *miR156*. Similar to *miR156*OE, *SPL13*RNAi plants withstood FS as manifested by their ability to maintain their physiological activities. It remains to be investigated whether other SPLs silenced by FS are directly involved in flooding response, and whether the effects of different SPLs are redundant or additive.

### ABA-dependent SnRK1 potentially regulates *miR156* for flooding tolerance

In our study, flooding caused an increase in ABA metabolites and upregulation of ABA biosynthesis genes. Due to the conserved ABA signalling pathway in plants^41^ we investigated the exogenous ABA application effect on SnRK1 using ABI insensitive *Arabidopsis* mutant seedlings (*abi1-2, abi5-8*) (due to the lack of similar mutants in alfalfa). The results showed a decrease in *KIN11* (one of the catalytic subunits of SnRK1) expression due to the combined effect of ABA and reduced sugar concentration. *KIN11* was more significantly reduced in *abi5-8* than in *abi1-2* mutant seedlings. This could be explained if the other calcium binding ABI (ABI2) is still active in the *abi1-2* mutant, complementing the defective ABI1. As KIN11 is affected by ABA and low sugar availability in *Arabidopsis*^13,14,18^, we investigated whether this response depends on the level of *miR156* expression in alfalfa. *SnRK1* was induced in both WT and *miR156*OE alfalfa genotypes in response to ABA treatment, with the highest level in WT. Under ABA treatment, the expression levels of the catalytic (*KIN11*), regulatory (β and γ), *SnRK1* and *SnRK2* were comparable between the genotypes under control condition, except for *SnRK2* which was significantly higher in *miR156*OE. Interestingly, under ABA treatment, the expression of SnRK1-associated genes (*SnRK2, KIN11, SnRK1*-γ subunit, SnRK1-β2 subunit) in *miR156*OE was similar to that in WT. This suggests the expression of *SnRK1* may not depend on the level of *miR156* expression, but SnRK1 may act upstream of miR156.

Due to the highly conserved SnRK function in plants^13^, and the lack of alfalfa mutants with altered KIN10 expression, we determined whether the expression of *miR156* depends on SnRK1 using *Arabidopsis* with silenced (via RNAi) or overexpressed *KIN10*. When the plants were subjected to ABA, expression levels of *miR156* was significantly higher in *KIN10* overexpressing plants compared to WT or *KIN10*RNAi plants under control or ABA treatment conditions. The observed ABA-dependent expression of *SnRK1* catalytic subunits (*KIN10* and *KIN11*) with *KIN10* overexpression resulting in higher *miR156* levels suggests that SnRK1 may upregulate *miR156* in *Arabidopsis*. Despite the evidence we showed in *Arabidopsis,* investigating the direct effect of ABA-dependent SnRK1 on miR156 using transgenic alfalfa genotypes with altered expression of SnRK1 is still indispensable. To understand how SnRK1 could increase the expression of *miR156* in vivo, it is necessary to investigate possible protein–protein interaction between SnRK1 and miR156 biogenesis proteins (such as DCL1, SE and HYL1). In our hands, we did not detect a pairwise protein–protein interaction between SnRK1 and DCL1 and SE. This could be associated with the possibility that more than two protein components may be required for interaction to occur, as reported among three cellulose synthase catalytic subunits (irregular xylem 1, 3, and 5) of CesA family for the proper assembly of the cellulose synthesizing complex^67^, or that SnRK1 may interact with other miR156 biogenesis proteins in alfalfa. Consistent with a possible role for SnRK1 in miR156 biogenesis, inactivation of the redundant SnRK2 kinases under stress inhibits the biosynthesis of miR156 and other microRNAs involved in stress tolerance^56^.

## Conclusion

Of the different phytohormones involved in abiotic stress response, ABA’s role has been well documented^68^. In our study, we found an enhanced level of ABA in flooding tolerant genotypes. ABA-dependent stress tolerance in plants involves regulating genes that control various plant functions, including REM that triggers protein kinase SnRK1. In our study we found an enhanced level of *SnRK1* in alfalfa under FS, and that expression of its α subunits *KIN10* and *KIN11* was ABA-dependent. Sensing REM associated with reduced photosynthesis dictates the metabolite dynamics, considering that the major carbon skeleton of ROS scavenging secondary metabolites, such as phenylpropanoids, is derived from sugar. In our study, a reallocation of resources from energy metabolism into secondary metabolism (anthocyanin) was observed at the transcriptomic and metabolic levels. Similar to the drought stress inducing anthocyanin through regulating miR156/SPL13-WD40-1/DFR^24^, there was an enhanced level of *miR156* upon flooding in alfalfa dictating an enhanced anthocyanin biosynthesis to scavenge ROS. Enhanced levels of *miR156* and *miR157* were reported in alfalfa upon drought stress^69^, suggesting a close functional overlap between miR156 and miR157. Whereas our investigation was focussed on the role of miR156 in flooding tolerance, we cannot rule out a redundant or additive role for miR157 in response to this stress.

**Figure 7:**
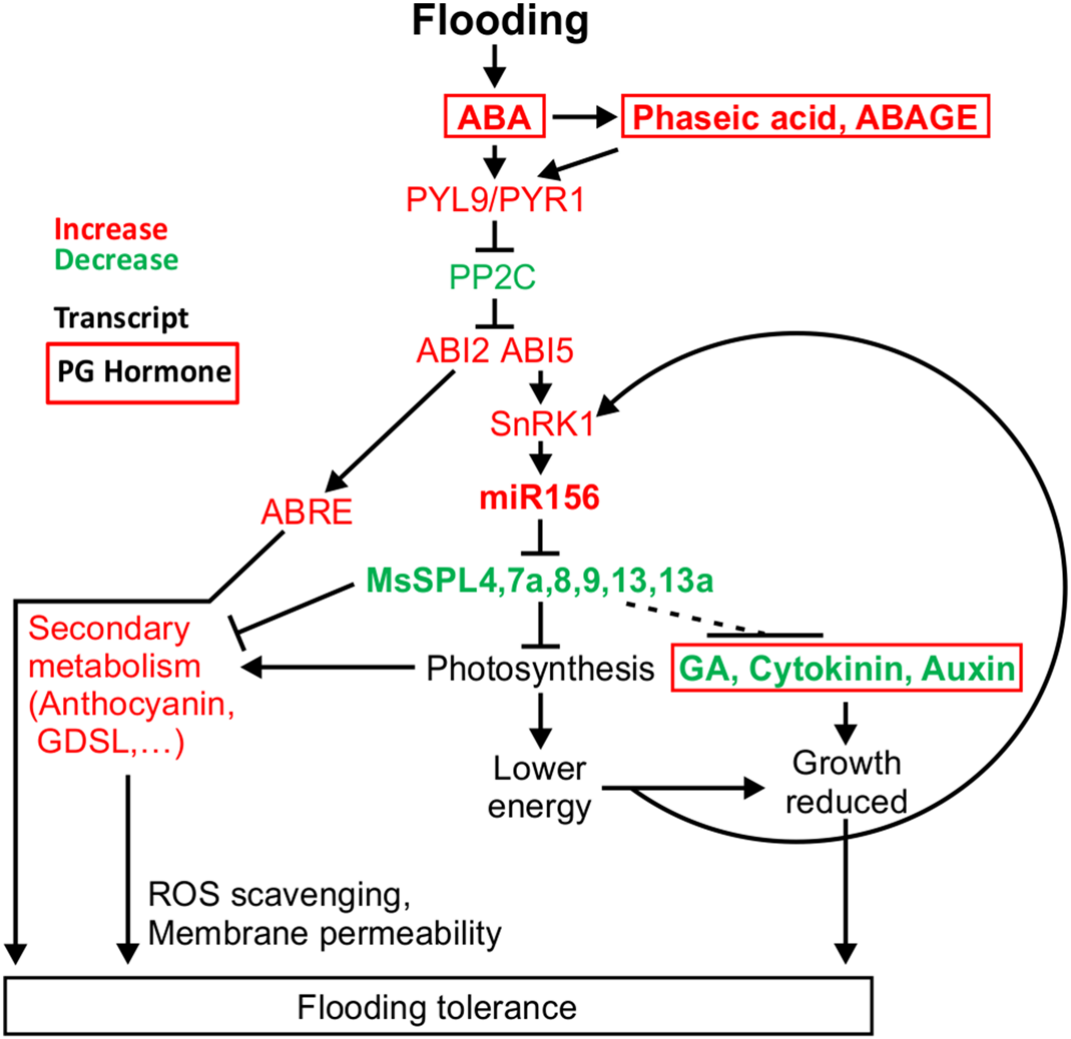
Proposed model for flooding tolerance in alfalfa. In flooding-tolerant cultivars, ABA is induced upon FS triggering PYL/PYR9 signaling molecules. This signaling pathway induces ABI2 and ABI5 while reducing the phosphorylated PP2C for increased expression of ABA-responsive elements, *ABRE*. ABA also induces a REM protein kinase SnRK1 along with its α and β subunits (*KIN10* and *KIN11*) through ABI5. The increased expression of *SnRK1* may trigger *miR156* and channels resources towards the phenylpropanoid pathway. Induced *miR156* expression silences SPL13, to mediate anthocyanin biosynthesis by its interaction with *DFR*^24^, and other SPLs (SPL4, 7a, 8, 9 and 13a). The increased abundance of ABA metabolites antagonistically affects gibberellic acid, cytokinin and auxin hormones. To exploit the newly identified SPLs (1, 1a, 2a, 7, 7a, 8, 13a, 14 and 16) in alfalfa breeding, it will be necessary to investigate their functional roles individually and in combination in response to FS and other physiological responses.

We propose that the reduced but continuous photosynthesis assimilation in flooding-tolerant genotypes maintains the carbon skeleton demand to produce secondary metabolism. An enhanced level of SnRK1 governed by ABA and REM may enhance the *miR156* expression to regulate the downstream genes in alfalfa. Subsequently, the enhanced level of *miR156* upon FS silences three newly identified SPLs (SPL7a, SPL8, SPL13a) and three previously identified SPLs (SPL4, SPL9, SPL13) to regulate downstream genes and physiological functions. Based on our present results and others in the literature, we propose a model through which the response to FS is regulated in alfalfa via miR156 (Fig. 7).

## Supplementary Information

Supplementary Figures.

Supplementary Tables.

## Abbreviations

ABAGE: ABA-glucose ester
Abi: ABA insensitive
ACC1: Acetyl-CoA carboxylase
DCL: DICER-LIKE
DEG: Differentially expressed genes
*DFR*: *Dihydroflavonol 4-reductase*
*DIN*: *DARK INDUCED*
*F3H*: *Flavanone 3 hydroxylase*
*F3′5H*: *Flavanone 3′5′hydroxylase*
FS: Flooding stress
GA: Gibberellic acid
GDSL: Gly-Asp-Ser-Leu (GDSL)-like lipase/acyl hydrolase
GSI: Gene-specific inner reverse primer
GSO: Gene-specific outer reverse primer
HXK1: Hexokinase1
*HYL1*: *HYPONASTIC LEAVES*
*J*_max_: Maximum photosynthesis electron transport rate
*KIN10 and KIN11*: Catalytic subunits of non-fermenting-related protein kinase1
miR: MicroRNA
NLS: Nuclear localization signals
PA: Phaseic acid
PYL: PYR-Like
PYR: ABA-like PYRABACTIN RESISTANCE
5′-RACE: Rapid amplification of cDNA ends
RCAR: REGULATORY COMPONENTS OF ABA RECEPTORS
REM: Reduced energy metabolism
ROS: Reactive oxygen species
Rubisco: Ribulose-1,5-bisphosphate carboxylase/oxygenase
SBP: *SQUAMOSA-PROMOTER BINDING PROTEIN*
SE: SERRATE
*SK1*: *SNORKEL1*
SnRK: Non-fermenting-related protein kinase
*SPL*: *SQUAMOSA-PROMOTER BINDING PROTEIN-LIKE*
*SUB1A*: *SUBMERGENCE1A*
TCA: Tricarboxylic acid
TMA: Total monomeric anthocyanin
*TPP*: Trehalose-6-phosphate phosphatase
*TPS*: Trehalose-6-phosphate synthase
UPLC-MS: Ultra-performance liquid chromatography-mass spectrometry
*V*_cmax_: Maximum rate of rubisco carboxylase activity
WD: Well-drained
WGCNA: Weighted gene co-expression network analysis
WT: Wild type
Y2H: Yeast-two-hybrid system
