# Supplementary material for: Involvement of the miR156/SPL module in flooding response in Medicago sativa
Source: Sci Rep. 2021 Feb 5;11:3243. doi: 10.1038/s41598-021-82450-7 (PMC7864954; doi:10.1038/s41598-021-82450-7)
Supplement: Supplementary file 1 — Supplementary Figures. [file 41598_2021_82450_MOESM1_ESM.docx]

**Involvement of the miR156/SPL module in flooding response in *Medicago sativa***

Biruk A. Feyissa^1,2^, Lisa Amyot^1^, Vida Nasrollahi^1,2^, Yousef Papadopoulos^3^, Susanne E. Kohalmi^2^, Abdelali Hannoufa^1,2,*^

**^1^** Agriculture and Agri-Food Canada, 1391 Sandford Street, London, Ontario, N5V 4T3, Canada

**^2^** Department of Biology, University of Western Ontario, 1151 Richmond Street, London, Ontario, N6A5B7, Canada

^3^ Agriculture and Agri-Food Canada, 58 River Road, Truro, Nova Scotia, B2N 5E3, Canada

^*^ Corresponding author email: Abdelali.Hannoufa@canada.ca


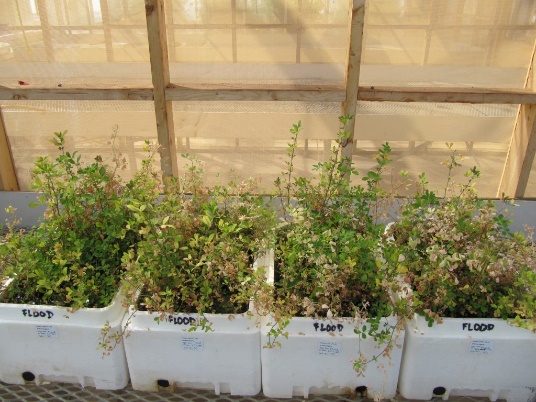

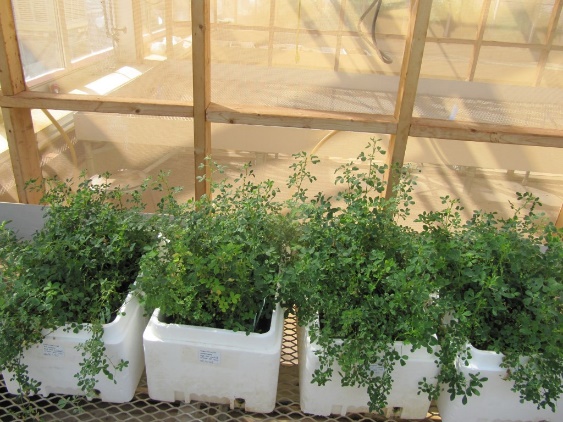

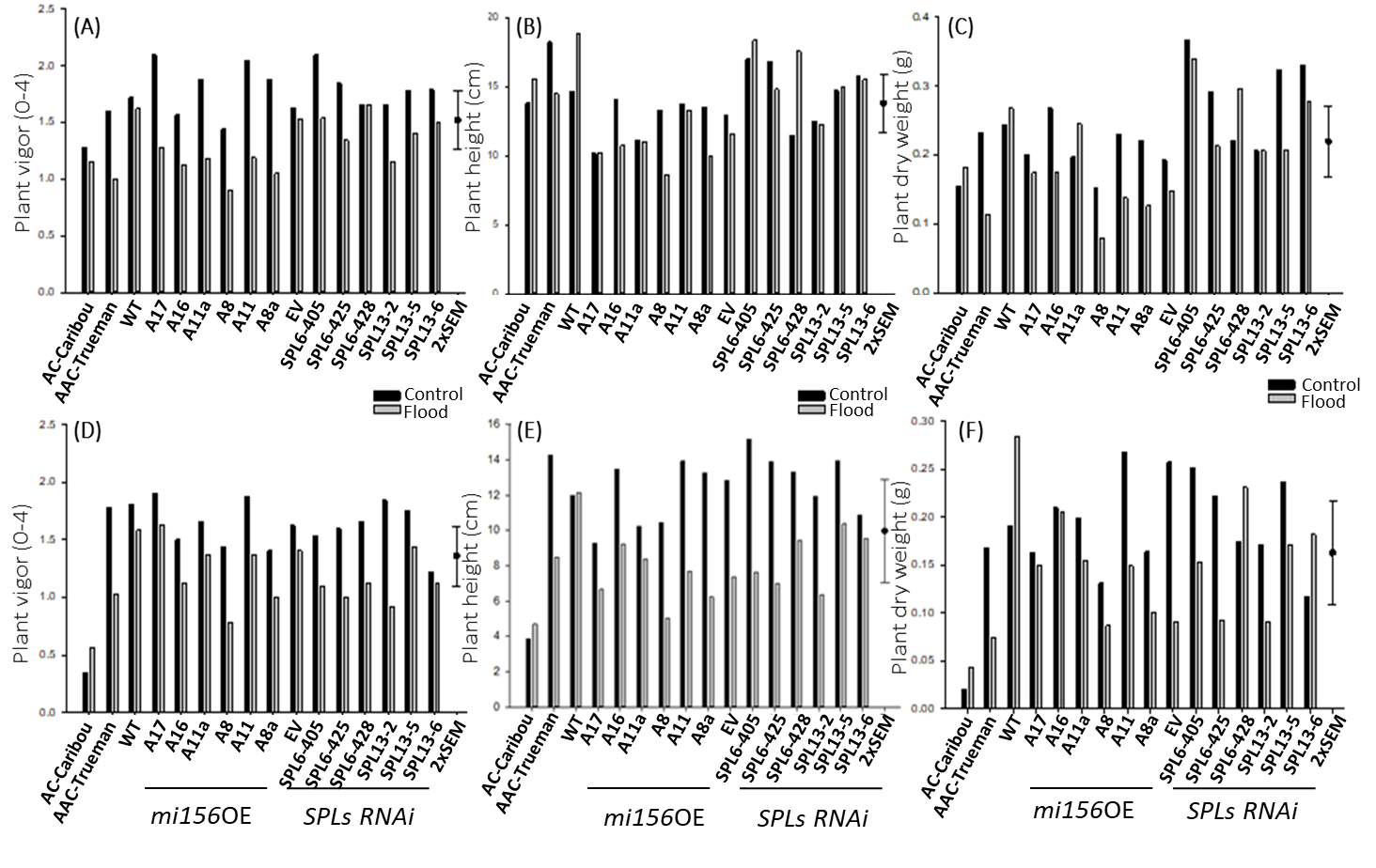


**(G)**

**(H)**

**Fig. S1.** Phenotypic responses of alfalfa genotypes to FS grown at AAFC, Nova scotia, Canada. (**A**) Plant vigor, (**B**) plant height, and (**C**) plant dry matter 6 week post flooding respectively, (**D**) Plant vigor, (**E**) plant, and (**F**) plant dry matter 21 weeks post flooding respectively, (**G**) Shoots of control, and (**H**) flood-stressed plants. In ‘**A**’ and ‘**D**’ plant vigor is scaled 1 to 4, four being exceptional. The above cultivars/genotypes were planted (July 1, 2016) in (6.4 cm^2^ wide and 14-cm height) root retainers containing Turface and Promix (50:50). Seedlings were clipped as needed and moved from the greenhouse to the field location (August 31, 2016). The experimental design consisted of 12 plants per experimental unit arranged in a split plot-randomized complete block design with 4 replications. The main plot was the flooding treatment (2); control (not flooded) and fall-spring flooding. The sub-plots were the genotypes/cultivars listed above. Plants were moved to the field location to allow the plants to acclimatize to field environmental conditions. The root retainers containing plants were placed in plywood boxes to accommodate designated flooding treatments which was initiated on October 15, 2016. At the end of the flooding treatment (6 weeks), the boxes were drained and the top growth trimmed back to 4 cm above the crown region. Genotypes used the field experiment are wild type (WT), empty-vector alfalfa, a local cultivar (AC-Caribou), FS-tolerant (AAC-Trueman), *miR156*OE (A8a, A8, A16, A11, A11a, A17), miR156-regulated *SPL6*RNAi (*SPL6*-405, *SPL6*-425, *SPL6*-428), and miR156-regulated *SPL13*RNAi (*SPL13*R-2, *SPL13*R-5, *SPL13*R-6).

**Fig. S2. Mean daily temperature in degrees Celsius for August 1, 2016 to November 31, 2016 at Kentville, Nova Scotia**. Source: Environmental Canada station KENTVILLE CDA CS. NOVA SCOTIA


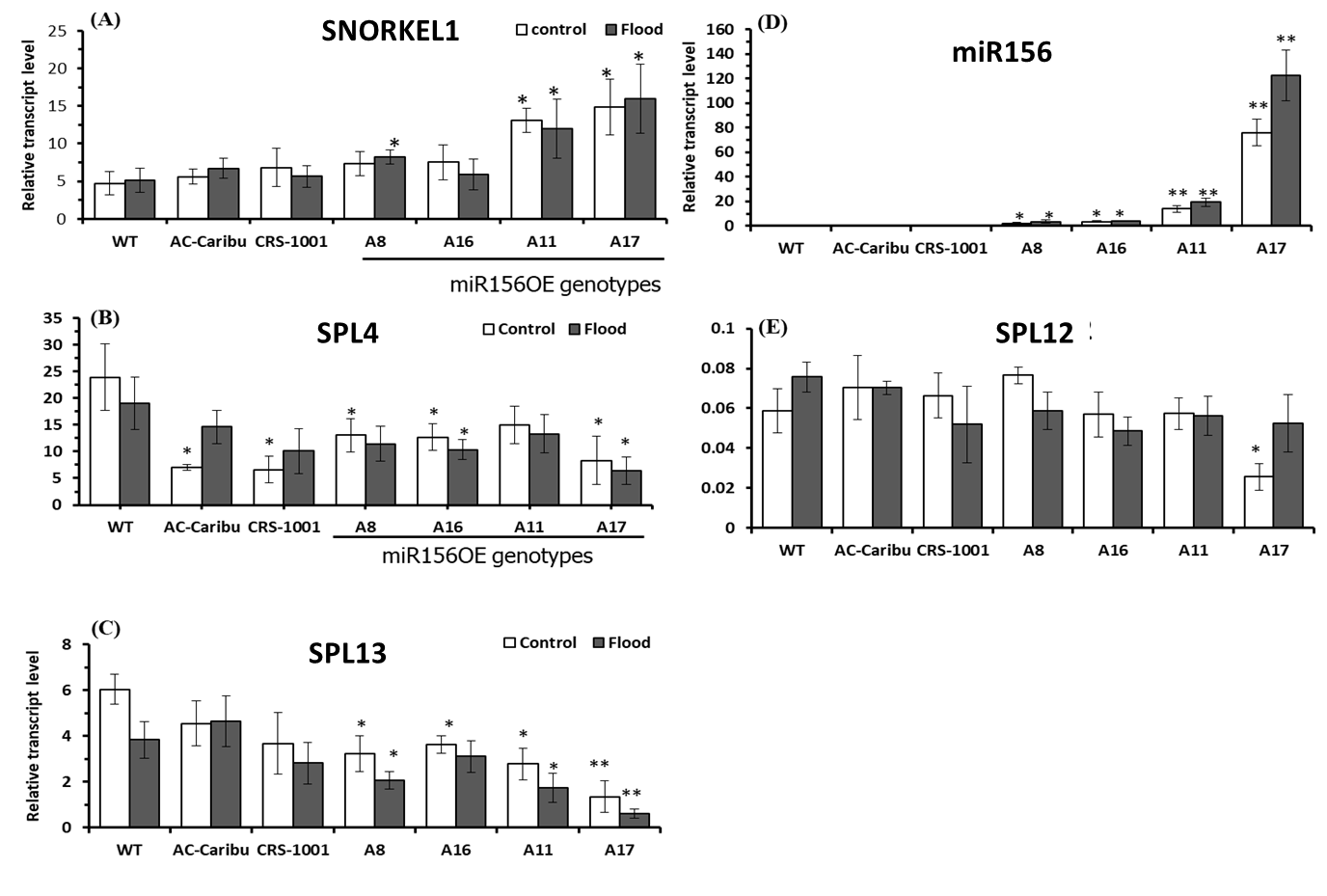


**Fig. S3. qRT-PCR-based transcript analysis of field grown alfalfa genotypes.** Relative transcript levels of **(A)** *SNORKEL1,* (**B**) *SPL4*, (**C**) *SPL13*, (**D**) *miR156*, and (**E**) *SPL12*. Pair-wise transcript level comparison was performed between WT and miR156OE genotypes to similar growth conditions. ‘*’ indicates significance level at p<0.05 level while ‘**’ indicates significant difference at p<0.01.


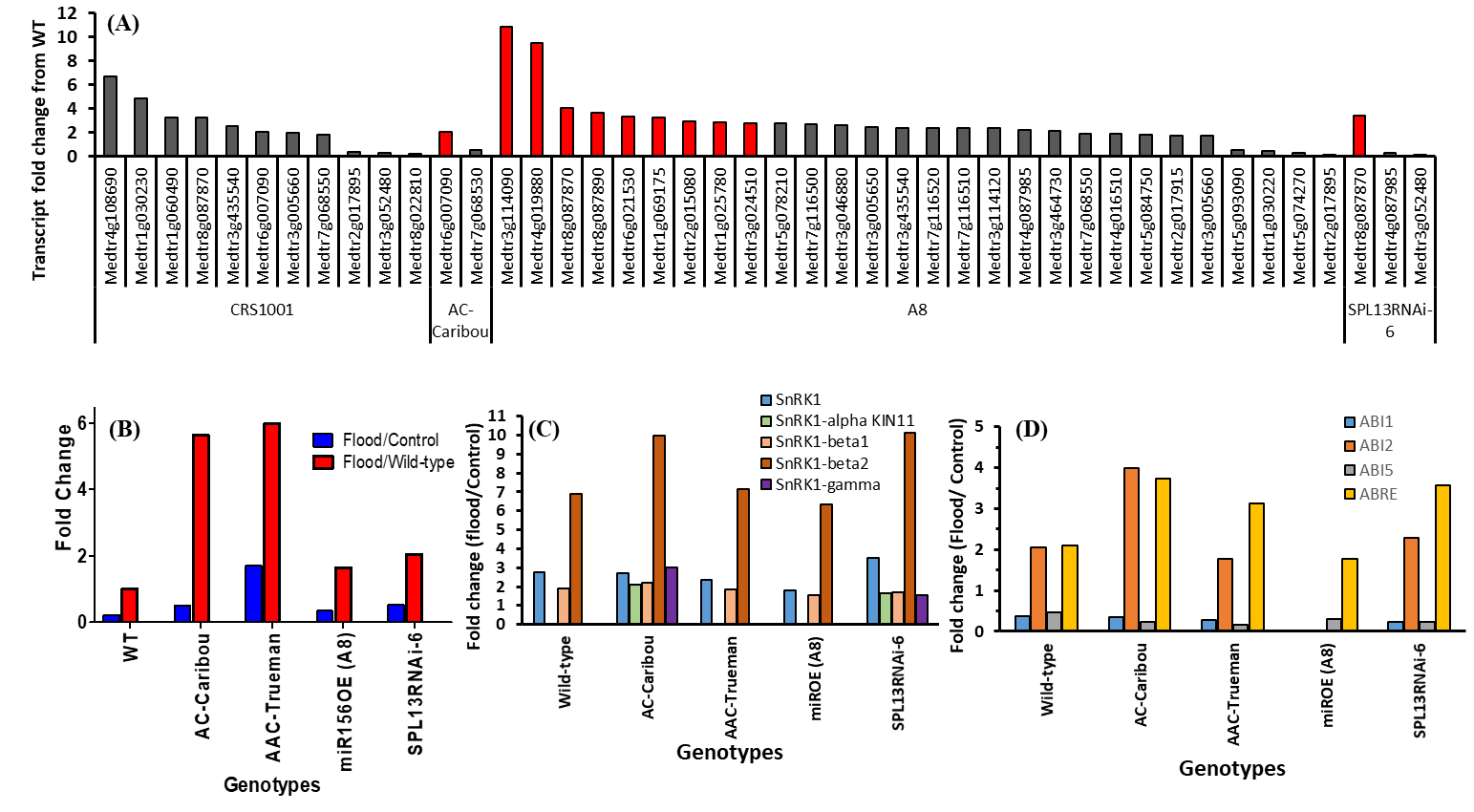


**Fig. S4 Fold-changes of commonly increased DEG among flood-tolerant genotypes.** Expression level fold-changes of (**A**) GDSL-like lipase/acylhydrolase and Pmr5/Cas1p GDSL/SGNH-like acyl-esterase, (**B**) Reticuline oxidase-like protein (**C**) sucrose non-fermenting-related protein kinase, SnRK1, related genes, and (**D**) abscisic acid insensitive (ABI1,2,and 5) and -responsive elements (ABRE). In ‘**C**’ SnRK1-alpha KIN11 are the catalytic subunits of SnRK1 while SnRK1-beta1 and -beta2 with -gamma subunits are regulatory subunits.


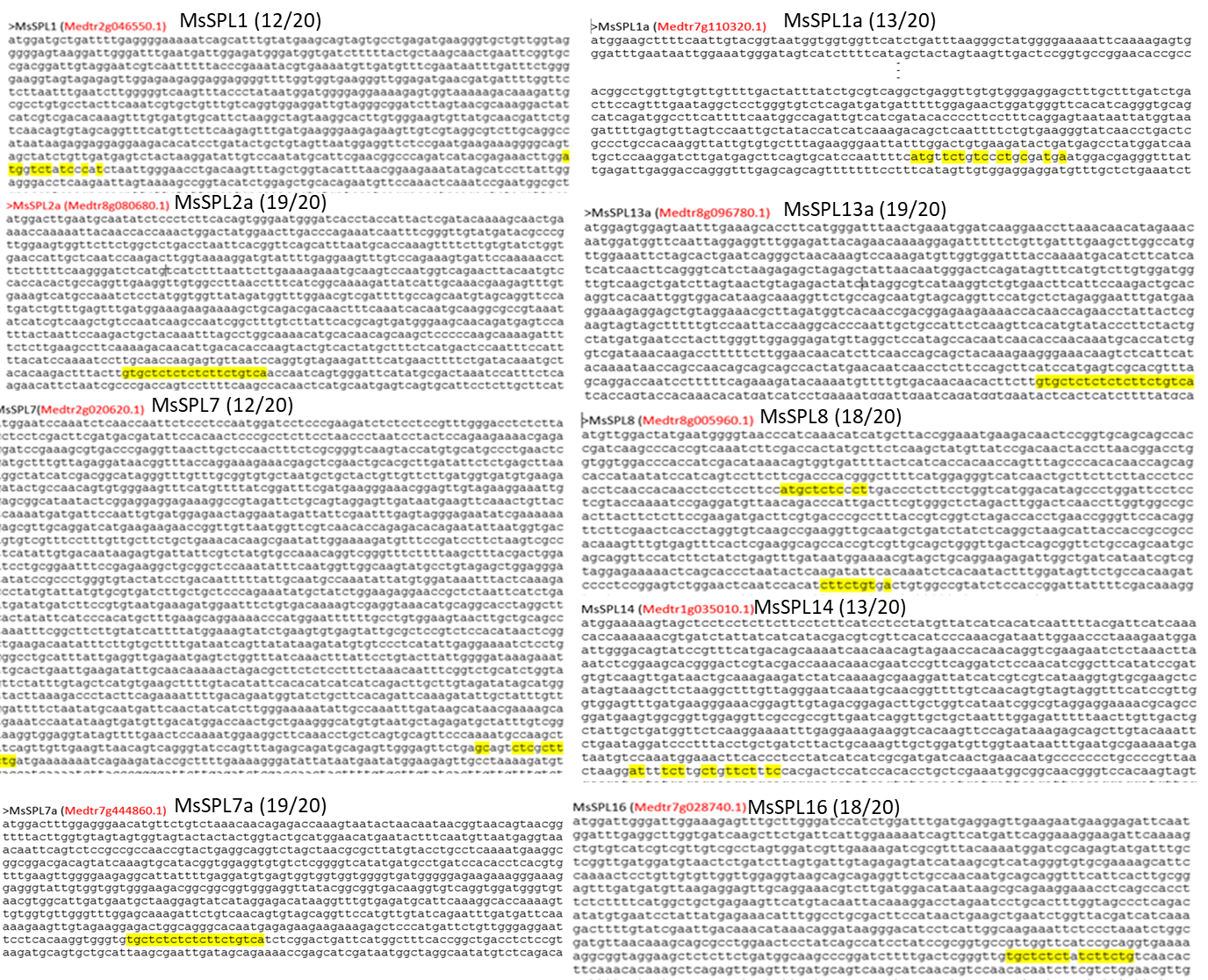


**Fig. S5 miR156 complementary nucleotide sequences of the newly identified MsSPLs.** miR156 complementary nucleotide sequences are highlighted yellow.


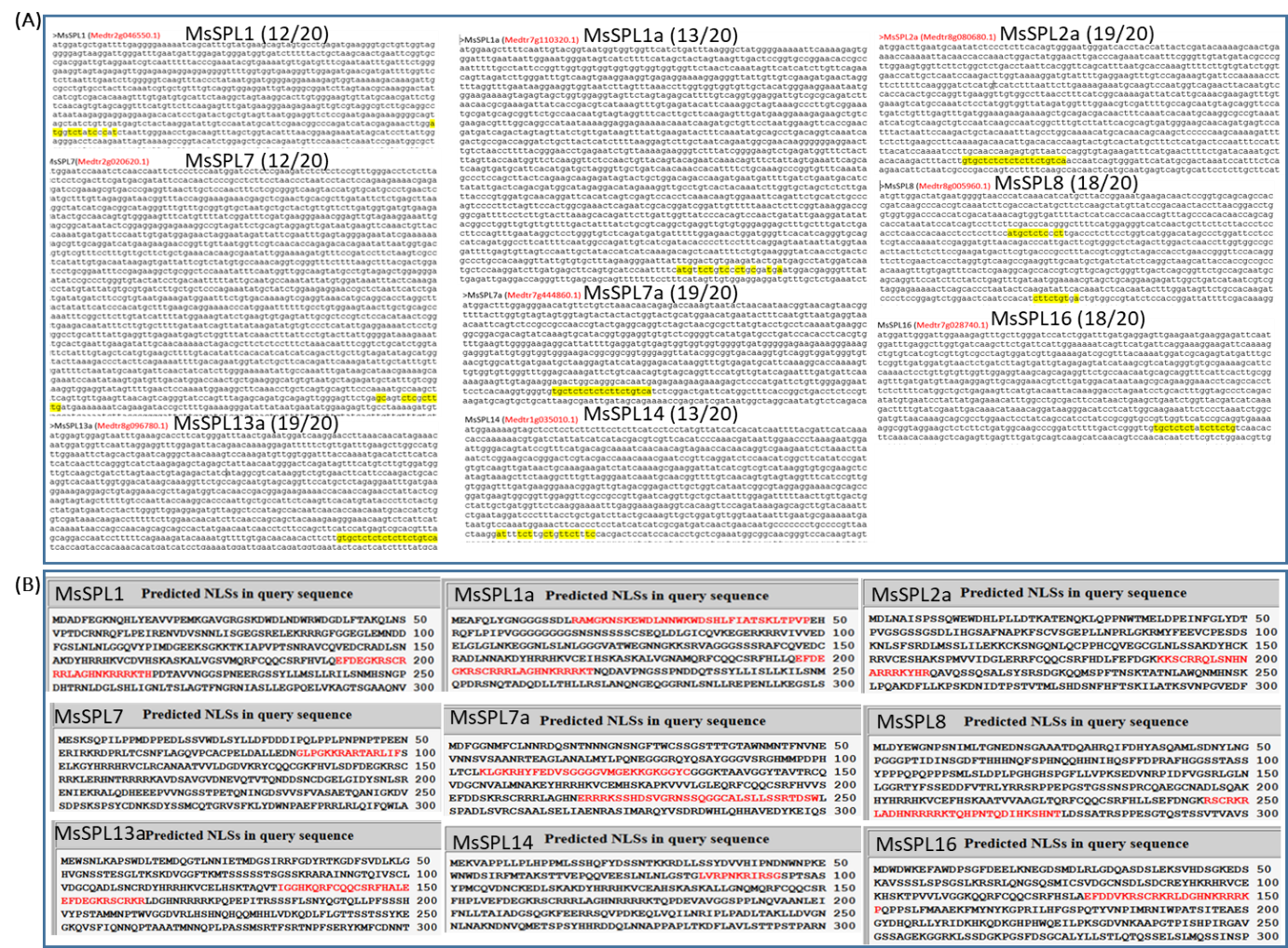


**Fig. S6 Nuclear localization signals (NLS) of newly identified SPLs.** Predicted nuclear localization signals from amino acid sequences are indicated in red fonts. Using an amino acid sequence-based online tool (*http://nls-mapper.iab.keio.ac.jp/cgi-bin/NLS_Mapper_form.cgi*), nuclear localization signals were also detected in the newly identified SPLs


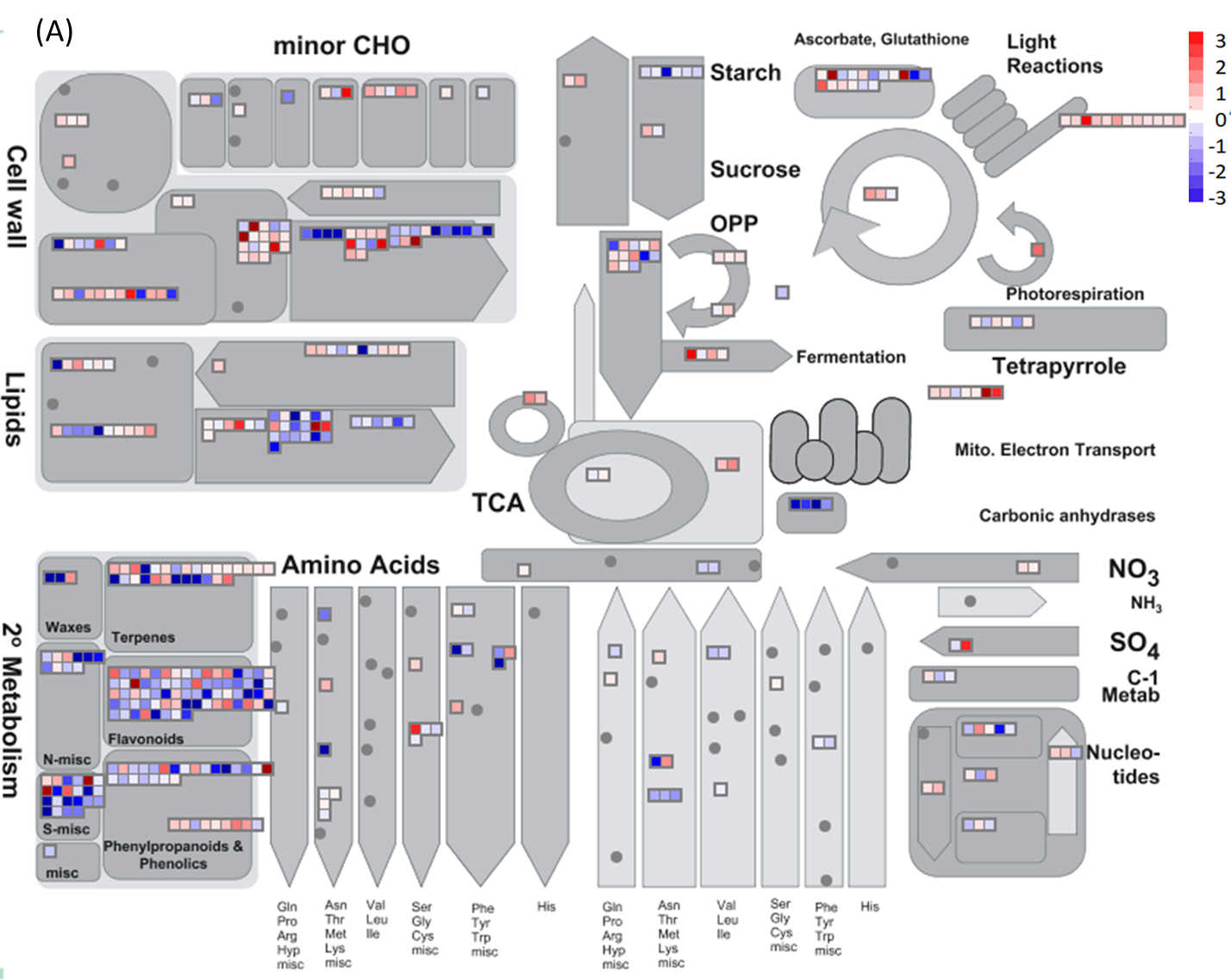


**Fig S7 DEG-based pathway analysis illustrate genotype specific flood stress regulation. (A)** Pathway analysis of DEG between flood-stressed AAC-Trueman and WT**,** (**B**) Pathway analysis of DEG between flood-stressed AC-Caribou and WT. Pathway overview is constructed using differentially expressed genes between AAC-Trueman and WT or AC-Caribou and WT under flooding conditions on MapMan online tool. Red colour indicates increased in transcript levels while blue are reduced levels. Values are transformed by log 2 values.


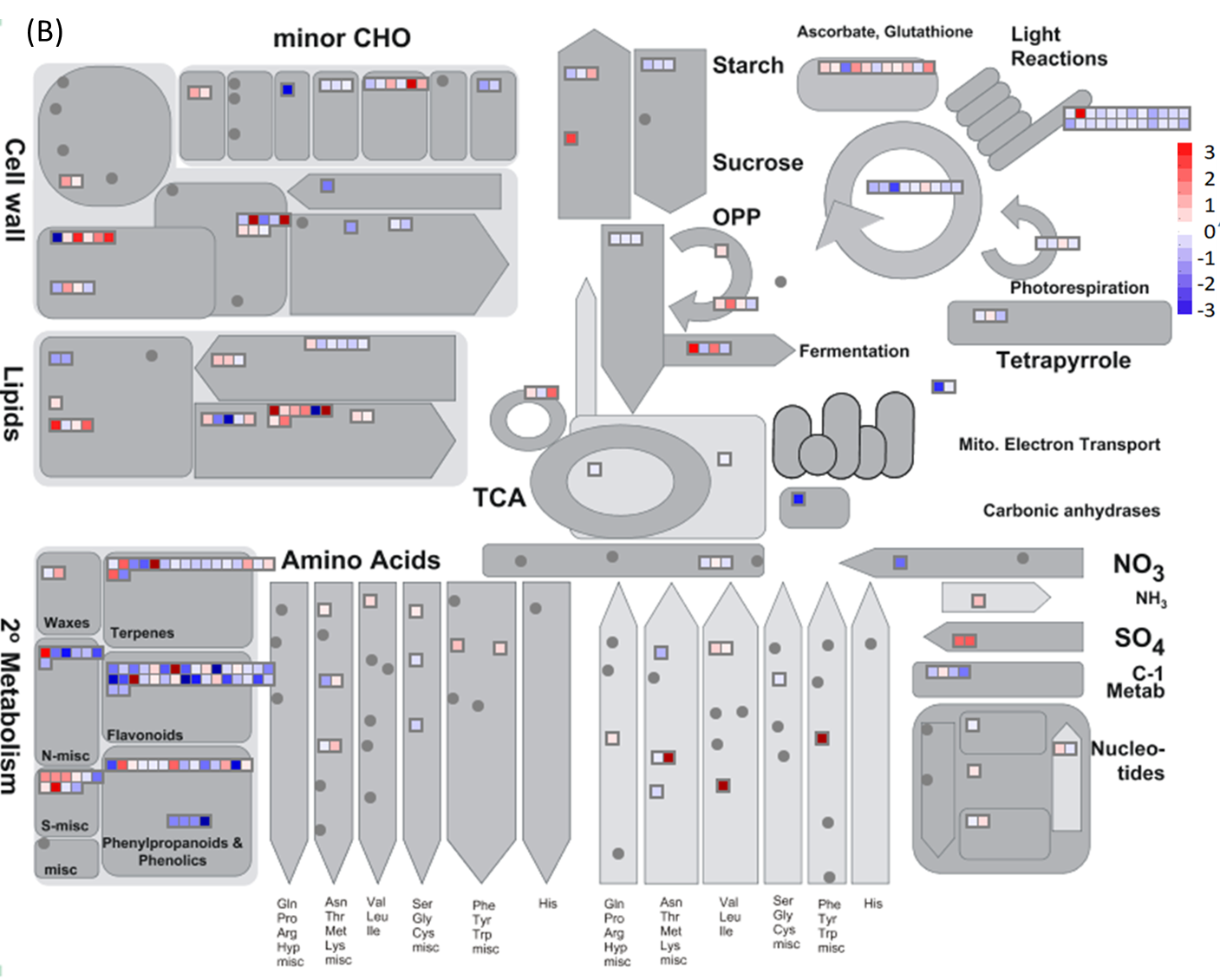


**Fig S7 DEG-based pathway analysis illustrate genotype specific flood stress regulation. (A)** Pathway analysis of DEG between flood-stressed AAC-Trueman and WT**,** (**B**) Pathway analysis of DEG between flood-stressed AC-Caribou and WT. Pathway overview is constructed using differentially expressed genes between AAC-Trueman and WT or AC-Caribou and WT under flooding conditions on MapMan online tool. Red colour indicates increased in transcript levels while blue are reduced levels. Values are transformed by log 2 values.
