# Supplementary material for: Involvement of the miR156/SPL module in flooding response in Medicago sativa
Source: Sci Rep. 2021 Feb 5;11:3243. doi: 10.1038/s41598-021-82450-7 (PMC7864954; doi:10.1038/s41598-021-82450-7)
Supplement: Supplementary file 2 — Supplementary Tables. [file 41598_2021_82450_MOESM2_ESM.pdf]

Involvement of the miR156/SPL module in flooding response in *Medicago sativa*

Biruk A. Feyissa[1,2], Lisa Amyot[1], Vida Nasrollahi[1,2], Yousef Papadopoulos[3], Susanne E. Kohalmi[2], Abdelali Hannoufa[1,2,*]

[1] Agriculture and Agri-Food Canada, 1391 Sandford Street, London, Ontario, N5V 4T3, Canada

[2] Department of Biology, University of Western Ontario, 1151 Richmond Street, London, Ontario, N6A5B7, Canada

[3] Agriculture and Agri-Food Canada, 58 River Road, Truro, Nova Scotia, B2N 5E3, Canada

[*] Corresponding author email: Abdelali.Hannoufa@canada.ca

Table S1. List of primers used in the study

No.	Gene name	Abbreviation	Forward primer sequence	Reverse primer sequence	Plant	Use	Source
1	miR156	miR156	cctatctctgcctgcttgacct	agcacccacttccacataacata	*Arabidopsis*	qRT-PCR	
2	KIN10 (At3g01090)	KIN10	agaatgatggcactgtgacg	acaggtgaagcaacgctt	*Arabidopsis*	qRT-PCR	Baena-González et al., 2007
3	KIN11(At3g29160)	KIN11	ccgtgttccaagtggctatc	acatgtgcaggaatccagtg	*Arabidopsis*	qRT-PCR	Baena-González et al., 2008
4	senescence-associated protein 1, DIN1 (At4g35770)	DIN1	cagagtcggatcaggaatgg	atttgaccgctctcacaacc	*Arabidopsis*	qRT-PCR	Baena-González et al., 2009
5	DIN3 (At3g06850)	DIN3	tcagctcgtcatctgtttgc	atcaaagtgggagggtgaac	*Arabidopsis*	qRT-PCR	Baena-González et al., 2010
6	glutamine-dependent asparagine synthetase dark inducible 6 (At3g47340)	DIN6	aacttgtcgccagatcaagg	ggaacacgtgcctctagtcc	*Arabidopsis*	qRT-PCR	Baena-González et al., 2011
7	raffinose synthase familydarkinducible 10 (At5g20250)	DIN10	gattcttcgtgctcgactcc	ttagcaagctgacaccatcac	*Arabidopsis*	qRT-PCR	Baena-González et al., 2012
8	SNF1-related kinase (Medtr1g034030)	SnRK1	ggagaccctgcacagagaat	ctgaaactgctcgcttgttg	*M. truncatula*	qRT-PCR	
9	SNF1 alpha kin 11 (Medtr6g048250)	SnRK1-KIN11	ttctggtttgagccgtatcc	gttgcctcattcctactccttg	*M. truncatula*	qRT-PCR	
10	SNF1-related kinase beta (Medtr5g098510)	SnRK1-b1	ggccttgctaagagtaggtcaa	actcaggagcatgtgtccatt	*M. truncatula*	qRT-PCR	
11	SNF1-related kinase beta 2 (Medtr2g095290)	SnRK1-b2	tcggctatcctgctaatgtc	gcgatgagtgtatcccaaag	*M. truncatula*	qRT-PCR	
12	SNF1 gamma subunit (Medtr4g103550)	SnRK1-g	tttcgatgggctccgttt	aacccaagtctaccacaggaac	*M. truncatula*	qRT-PCR	
13	SNF2, helicase and zinc finger protein (Medtr2g012830)	SnRK2	aagaccaaaggcacacga	aaagaggaacagccagca	*M. truncatula*	qRT-PCR	
14	Snorkel	SnorkelERF	ttccagaggagggtagttaaga	ttggtgcacagagtgattcc	*M. sativa*	qRT-PCR	
15	MsSPL1 (Medtr2g046550)	MsSPL1	agggttggagatgaacgatg	cacaggcgcaatctttgtct	*M. truncatula*	qRT-PCR	
16	MsSPL1a (Medtr7g110320)	MsSPL1a	gtgaggagaaatagcaggcagt	cccacagcatcaggtctaaa	*M. truncatula*	qRT-PCR	
17	MsSPL2	MsSPL2	gtagaagaggaggagggtgatg	gcaacagggtggagaaacac	*M. sativa*	qRT-PCR	Gao et al., 2016
18	MsSPL2a (Medtr8g080680)	MsSPL2a	gctgcagacgacaactttca	catctgttgcttcccatcac	*M. truncatula*	qRT-PCR	
19	MsSPL4	MsSPL4	atgccaaagcacctaccgt	cctctcattatgcccagcca	*Msativa*	qRT-PCR	Gao et al., 2016
20	MsSPl7 (Medtr2g020620)	MsSPL7	ccctgaactcgatgctttgt	gccgtcgatgatagcctttta	*M. truncatula*	qRT-PCR	
21	MsSPL7a (Medtr7g444860)	MsSPL7a	cagggcacaatgagagaaga	gccatgaatcagtccgagat	*M. truncatula*	qRT-PCR	
22	MsSPL8 (Medtr8g005960)	MsSPL8	tggccgcacttacttctctt	aaccttcggcttgacaccta	*M. truncatula*	qRT-PCR	
23	MsSPL9	MsSPL9	ggaagaggtgggtcagttca	acagtaacagttgggcacttag	*Msativa*	qRT-PCR	Aung et al., 2015b
24	MsSPL13	MsSPL13	caccatggagtggaatttgaaagc	ctattcccattgatagggaaatagt	*Msativa*	qRT-PCR	Aung et al., 2015b
25	MsSPL13a (Medtr8g096780)	MsSPL13a	cttgggttggaggagatgtt	gctgctggttgaagatgttg	*M. truncatula*	qRT-PCR	
26	MsSPL14 (Medtr1g035010)	MsSPL14	gatctccaacatcggcttca	gagcttcgcacaccttatga	*M. truncatula*	qRT-PCR	
27	MsSPl16 (Medtr7g028740)	MsSPl16	tttgactcgggttgtgctct	cgttccagacgaagattgtg	*M. truncatula*	qRT-PCR	
28	MsSPL7a (Medtr7g444860)	MsSPL7aGSP	gagctcccatgattctgttg	-	*M. truncatula*	5' RLM-RACE	
29	MsSPL7a (Medtr7g444860)	MsSPL7aGIP	-	cctagccattatcgatgctc	*M. truncatula*	5' RLM-RACE	
30	MsSPL7a (Medtr7g444860)	MsSPL7aGOP	-	ctgcaaatgccagtctctgtct	*M. truncatula*	5' RLM-RACE	
31	MsSPL8 (Medtr8g005960)	MsSPL8GSP	atggagggtcatcaactgct	-	*M. truncatula*	5' RLM-RACE	
32	MsSPL8 (Medtr8g005960)	MsSPL8GIP	-	cacgaagtcaatgggtctgt	*M. truncatula*	5' RLM-RACE	
33	MsSPL8 (Medtr8g005960)	MsSPL8GOP	-	ttcgaagaacctgtggaacc	*M. truncatula*	5' RLM-RACE	
34	MsSPL13a (Medtr8g096780)	MsSPL13aGSP	tccatgagtcgcacgtttag	-	*M. truncatula*	5' RLM-RACE	
35	MsSPL13a (Medtr8g096780)	MsSPL13aGIP	-	caggatgatcatgtgtttgtg	*M. truncatula*	5' RLM-RACE	
36	MsSPL13a (Medtr8g096780)	MsSPL13aGOP	-	ccactctgatcatttgaaccca	*M. truncatula*	5' RLM-RACE	
37	Zinc finger protein SERRATE (SE) (Medtr8g043980)	SE	caccatggcagaagttctcacccc	tatcacggtgacctcttcgtct	M. truncatula	Yeast-two-hybrid	
38	Zinc finger protein SERRATE (SE) (Medtr3g006760.1)	SE	caccatggccgaagtcgaaaatct	gtcttgataactccgcatcc	M. truncatula	Yeast-two-hybrid	
39	DCL protein (Medtr3g102270.2)	DCL1	caccatggccgcaccacttcttct	ccaaacagggccttagtatctg	M. truncatula	Yeast-two-hybrid	
40	SNF1-related kinase (Medtr1g034030.1)	SnRK1	caccatggatcctactatgaacca	ctcgtccatttcgtcatcga	M. truncatula	Yeast-two-hybrid	
41	Acetyl CoA Carboxylase1	*Acc1*	gatcagtgaacttcgcaaagtac	caacgacgtgaacactacaac	*M. sativa*	qRT-PCR	
42	Actin	*Actin*	tcaatgtgcctgccatgtatgt	actcacaccgtcaccagaatcc	*M. sativa*	qRT-PCR	

Table S2. The matching gene IDs of the newly identified MsSPLs in the tetraploid alfalfa genome

MsSPL	Chromosome	Gene ID	MsSPL	Chromosome	Gene ID
1	2.1	MS.gene98509	7a	7.4	MS.gene93246
1	2.2	MS.gene033954	8	8.2	MS.gene61571
1	2.3	MS.gene59294	8	8.3	MS.gene000259
1	2.4	MS.gene76798	8	8.4	MS.gene022540
1a	7.1	MS.gene050425	8	8.4	MS.gene62778
1a	7.2	MS.gene28650	13a	4.1	MS.gene40685
1a	7.3	MS.gene050856	13a	4.2	MS.gene030560
1a	7.4	MS.gene043764	13a	4.3	MS.gene79945
2a	4.2	MS.gene023425	13a	4.4	MS.gene030453
7	2.1	MS.gene002799	14	1.1	MS.gene026366
7	2.2	MS.gene96180	14	1.2	MS.gene040800
7	2.3	MS.gene00967	14	1.3	MS.gene061710
7	2.4	MS.gene004483	14	1.4	MS.gene061104
7a	7.1	MS.gene36181	16	7.1	MS.gene000446
7a	7.2	MS.gene010796	16	7.2	MS.gene99828
7a	7.3	MS.gene38329	16	7.3	MS.gene001948

Table S3 coding sequences used for yeast-two-hybrid assay between SnRK1 and miR156 biogensis genes

>Medtr8g043980.1 (zinc finger protein SERRATE (SE) (Medtr8g043980)

atggcagaagttctcaccccccattccctccgattccctcgaccaatcctccccaaccaccacctcccctccaccaccgcctccacaaccacaatcacctcctcccacctccgcccccgattccgatctccctctaccacctccacaatcctccttccgccgcaga
gactaccgccgcgatgatagagatagagatttcgatcgtcctcctcctcgccgcgagtattatgaccgtaacaacatatcgcctccgagggatagagatagagatttcaaacgcaggcgtagtcccagtcctcctccatatcgtgaccgccgtcattcaccacca
atgcgaaggtcgcccccgcattataataattataaacgctccaggagaggtggtagtccccgtggcggttatggacccgatgatagatctgaatatgataattacggtggttatgaaaggggtggaagaggtggttatgataggggtggaagaggtggctatgat
aggggtggacgaggtggtaatgataggggtggaagaggtggctatgataggggtggaagaggtggttatggtgatgacaggtcttatggtaggtttggaaatcgttctggaggaggatatcaaaatggggtctctggtatggaatccaatcgtggttatgcagac
ttgccaagtggcggtgggcaaagagaagggttgatgtcctataaacaattcattcaggagctggaggatgatatactaccaactgaagcagaacgtaggtatcaagaatacaaatcagagtatatttctacacaaaaaagagcttatttcagtgctcataaggat
gaggagtggttgaaagataaatatcatccaacaaatttgcttacagtcattgaaaggaggaatgaaagtgctcgacagctggcaaaggatttttgcttgatttgcaaagtggaacattagatccaaatccgggtttaagtgcctctttatccagcaaatcaggg
caagctagcgagccaaattcagaggaggaagcggaagaccaaagaagaagagatgggtggggttctaataaaaaaagtgatttctcagctgctccaaaggctcatcctgtcagttctgaaccaagacgaatacaagcagatgttcaacaggctcaggcccttgtt
cgcaagcttgacacagaaaagaggattggagataatatttatgcagcatcattataagaggcctgacatctgttaagggtttagaaggtgttgagcttttggacacactgattacatatctgtggcggattcatggtgtagattattatggcatgattgagact
aatgaggctaagggttttaggcatgttaggccagaaagaaaagggcatgaagaaacaggtaaatcagggtctgaatgggagaagaaacttgactcattttggcagggaaggttgaatggtcaggatccattggaagtactggctgctaaggagaaaatggatgct
gcagcaattgaagtgttggatccatatgttagaaaaatcagggatgaaaagtatggatggaagtatggttgtggagctaagggctgcacaaagctttttcatgctgctgaatttgtgtacaaacatctgaagctaaagcaccctgagcttgctcttgaacaaact
tcgaaattgcgtgaggatctctatactcaaaattacatgaatgatccagatgctcctggtggaaagcctgtcatgcagcaacctcagaaggacaacaggcctttaaatcaaaggttaggttttgagggccgattgagagatgatcgtggtaatcgtcgagaccat
gaacggtatgacagaatgaatggagatagagctaattccccatcccgtgaaaggcagtctaaagcacttgagatgggaaaccatgatgaaacaatgcaagacactttgcagggcctggtgttcctcctttagctcagatattcctccaccagtattgatgcct
gtacctggtgctgggccattaggaccttttgttcctgctccaccagaagttgcaatgcagatgtttagagatcaaggaggaccatcttcatttgatggctctggaataaatatgctgtctggcccgcatcatatgggtggaccggcaccaataattactcttcct
ccaggttttagaccagatcctcggaagatgcgaagttatcaagatttggatgctccagacgaagaggtcaccgtgatagactatcggagtttgtaa

>Medtr3g006760.1 C2H2 zinc finger protein SERRATE (SE) (Medtr3g006760.1)

atggccgaagtcgaaaatctccctcccgaatcacacgaccgatctcccaccatcaccaccgctccacctcctcccgacgacgatctcccacctccacctaatcccaaccctcgtcgtcgcgatttcgaccgtcgtgacgacagagatttcgaccgccgtcctcac
cgtggcggccgtggtggccgcggtggcggtggttactacaacaaccgtgatagagacatcaaacgccgtcgtagtcctagccctaactatcgtgaccgccgctattctcctcctccgcaacagtctcgtcgttctcctccgccgtataagagatctagaaggggt
agtcctcatagacctaacgacaggtttggccatgatcactttggtggctatgaacggggaggtggaaggggtggctatgcagatgatagacctggtggcagatttccacatcgatctggagggggatatcaaaacggaatttctgatttggatgccaatcgcggt
tatgcagatttgccaagtgggggtgctcgaagagagggtttgatgtcctataagcaattcatccaggagcttgaagatgatatactgccatctgaagctgagcgtagataccaagagtacaagtcagagtatattactacccaaaaacgagcttatttcaatacc
cacaaggatgctgagtggttgaaagataagtatcatccaacaaatttacttacagtaattgaaaggaggaatgaaaatgctcgacgtttggcaaaggagtttttgcttgatttgcaaagtggaacactagacataaatccaggtttaaactctgcatccactaaa
tcagagcaagccactgagcccaattcagaggaggaaacagatgccggtggtaaaagaagaagacatggcaggggatctaataaggacaacgatttctctgctgctccgaaggctcaccctgtcagttctgaacctagaaggatacaagaagatattcatcaggtt
caggctgttgttcgtaagcttgacaaggaaaaagggattgaagataatattttatgcaccaatgatcatagtaaaaatagtgacaaggctcgcagtggatctgtgggccctgtagtaattatccgtggcttaacatctattaagggcttagagggtgttgagcta
ctggacacgcttataacatatctttggcggattcatggtgtagattattatggaatgcttgagactagtgatgccaaaggttttaggcatgtgagaccagaaggagcaaggcatgaagaaacagctaagtcggggtcagaatgggagaaaaaacttgattcattt
tggcagagaaggttggacggcctggatcccttggaagtaaggactgcaaaggagaagatagatgctgcagcagctgacatgttggatccatatgttagaaaaattagggatgaaaagtatggttggaagtatggctgtggagccaagagttgcacaaagcttttt
catgctgttgagtttgtgtacaaacatctcaagctaaaacacccagagcttgtgttggaactaacatcaaaattgcgggaagatctttattttcaaaattacatgaatgatcctgatgctcctggtggaacgcctgtcatgcagcaatctcaggacaaaccttta
aagcgaaggttactggaaggccgattgaaagatgatcgtgggaatcggcgagaccaggataggagcgacagaacaaatggagatagacctgacagttcgccatcccatgagaggcagttgggaaaccgtgatgaaacgatgttcgatgcatatggtggacctgct
gtatcattcaactcagatatgccccctcctccagttttgatgcctgtacctggtgccgggcctctgggaccctttgttcctgctccaccagaagttgcgatgcagatgtttagggatcaaggaccatcttcgtatgatccatcaggaatgacgatgcgttctggt
cctcacatagggggacaagcctctatgattgctgttcctcccacctttagacctgatccccgacggatgcggagttatcaagacttggatgccccagaagaagaagttactgtcatagactatcggagcttgtag

>Medtr3g102270.2 DCL protein (Medtr3g102270.2)

atggccgcaccacttcttctaagacaagctctgttacttcgcgcttgtttccataactacaaacacttcaccgtcagaaccgttacggcgcgccaccggctatggtgcagcgccgccgccatcaatccaccgtcggaaaaaccaaacgacaaacctaactccgtt
agcaacacgttaaaccggagagatgttttcccgtcgagtaacttccgtttggaggatgaggaatctgattatcgaaaatggaaggataaagaagatgagattttcaatgatattgaacctattattgtgcttactaaggaaattcttcattcatcaaagtatatg
gatggagggacattaactttggaggatgagaaaactgtagtggaaaagcttcttgcttatcatccacactctgaagataagattggatgtggtcttgaatccattatgatagactcaaactatgaagctcagacacggtgtcagacaccatacatatccgacaca
ggtgcacgcctaatccgaggagtatttgtgcttcatactcagataagtcaattcaaacagatactaaggccctgtttggataaacaacttattttcaccttatag

>Medtr1g034030.1 SNF1-related kinase (Medtr1g034030.1)

atggatcctactatgaaccatgttggacatggaggaatggatatacagattatgcatgagagtgatagatatgagcttgtacgtgatattggttctgggaattttggagtggctaggcttatgagagataaacacactaatcaacttgttgctgtcaagtatatt
gagagaggtgataagatagatgaaaatgtacaaagggaaattataaatcatagatcactgaggcatcccaatattgtcaggttcaaggaggtaatacttacccctacacatttagctattgtgatggaatatgcttccggaggagagctatttgagcgaatatgc
aatgcaggacggttcagcgaggatgaggcacggtttttcttccaacaacttatatcaggggttagctactgtcatgcaatgcaagtatgccatcgtgatttgaagttggagaatacattgcttgatggtagtccggctccccgtttgaagatttgtgattttggg
tattcaaagtcatcagtgctacattcacaaccaaaatctacggtcggtacccctgcttatatcgctcctgaagttttgctcaaaaaggaatacgatggcaagattgcagatgtgtggtcttgcggggtgaccttatatgtcatgttggtgggtgcgtatcctttc
gaggatccggaggagcctaaaaatttccggaagactattcataggattttgaaagtccaatactcaatacctgattatgttcaaatatctcccgagtgccgtcatctgatctcaaggatcttcgttggagaccctgcacagagaataagcatgccggagattcga
aaccatgattggttttgaagaaccttccagctgatcttatcgtggggaatacaacaagcgagcagtttcaggagcctgaacaaccaatgcaaacaattgaagaaataatgcagataattagcgaggctactattccagcagccggaactcatcagtctctcact
cagtatcttactggtagcttggacatcgatgacgaaatggacgaggatatagatactgatcccgatcttgatatcgatagcagtggtgaaatagtatatgcaatgtaa

The whole protein coding sequences of SnRK1 (Medtr1g034030.1), DICER-LIKE (DCL) (Medtr3g102270.2), and zinc finger protein SERRATE (SE) (Medtr8g043980) were amplified using gene specific primers (**Table S1**). The amplified coding sequences were cloned into the pENTR™ /D-TOPO® vector (Invitrogen) and transformed into *E. coli* by heat shock (42 ^{0}C for 90s). For subsequent Y2H assay, we used the ProQuest™ Two-Hybrid System (Invitrogen).

Table S4. Analysis of variance from physiological data obtained from the field experiment laid at Agriculture and Agri-Food Canada (AAFC) Research Centre in Kentville, Nova Scotia, Canada

Source	d.f.	Plant Ht, cm, 6wk post flood	Vigor, 0-4, 6wk post flood	DM, gm, 6wk post flood	Plant Ht, cm, 12wk post flood	Vigor, 0-4, 12wk post flood	DM, gm, 12wk post flood
Block.fPlot stratum	*	.	.	.	.	.	.
Flood	1	ns	0.089	ns	ns	0.059	ns
Residual	6	.	.	.	.	.	.
Block.fPlot.Tray.cPlot stratum	*	.	.	.	.	.	.
Cultivar	15	0.003	0.096	<0.001	0.066	<0.001	0.005
Flood.Cultivar	15	ns	ns	ns	ns	ns	ns
Residual	90	.	.	.	.	.	.
Block.fPlot.Tray.cPlot.Pouch.Plant stratum	*	.	.	.	.	.	.
PPlant	7	ns	0.060	<0.001	ns	0.003	0.006
Flood.PPlant	7	0.091	ns	ns	ns	ns	0.082
Cultivar.PPlant	105	0.076	ns	<0.001	<0.001	0.034	<0.001
Flood.Cultivar.PPlant	105	0.091	ns	<0.001	ns	ns	0.061
Residual	667	.	.	.	.	.	.
Total	1018	.	.	.	.	.	.

Table S5. Analysis of variance of hormone profiles in flood stressed and control alfalfa plants

Hormone	Genotype	trt	Genotype*trt
**ABA**	0.0123	NS	NS
**ABAGE**	7.23E-07	2.57E-07	0.0305
**PA**	0.00488	7.74E-06	0.02528
**Total ABA**	0.00655	NS	0.04869
**IAA**	NS	NS	NS
**Totalauxin**	NS	0.0151	NS
**GA19**	NS	NS	0.0076
**TotalGA**	0.041	NS	NS
**TotalCy**	0.0253	8.40E-09	0.00988
**DPA**	0.014654	0.000275	0.042277
**7OHABA**	0.014654	0.000275	0.042277
**tABA**	NS	NS	0.0229
**cZOG**	8.38E-05	NS	NS
**cZR**	0.0277	1.99E-09	0.0105
**iPR**	0.03413	2.39E-08	0.00908
**IAAAsp**	7.54E-05	5.49E-10	0.000512

NS: Not significant at $p<0.05$

Table S6. UHPLC-MS profiled hormones in ng/g dry weight up on flood stress in alfalfa leaf tissues

Phytohormones	Abbreviations	Wild-type		AAC-Trueman		A8 (miR156OE)		*SPL13*RNAi	
		Control	Flood	Control	Flood	Control	Flood	Control	Flood
ABA metabolites	ABA	6129.42	2879.37	3436.01	2494.99	1118.73	1871.10	2795.64	3319.30
	DPA	308.11	334.79	223.85	730.86	105.05	324.19	133.04	368.09
	ABAGE	50.40	373.88	401.74	1073.29	110.15	423.27	47.52	329.98
	PA	2724.02	2883.30	1173.19	3470.77	607.34	2252.90	758.16	2835.76
	7'OH-ABA	183.18	158.16	64.38	116.98	66.46	91.75	76.90	80.92
	neo-PA	8.74	ND	4.45	ND	ND	ND	3.90	4.00
	t-ABA	82.56	43.31	73.83	67.06	35.73	62.06	40.39	79.38
Cytokinins	t-ZOG	ND	ND	1.76	ND	2.64	2.23	2.89	1.62
	c-ZOG	18.75	17.43	6.69	9.59	13.49	14.02	16.47	14.97
	t-ZR	1.00	ND	0.95	0.90	2.40	1.50	1.00	ND
	c-ZR	109.14	13.91	74.15	18.77	60.00	14.70	69.54	21.36
	dhZR	2.45	1.23	3.13	ND	2.14	1.40	3.99	1.30
	iP	1.13	1.10	1.35	1.00	ND	1.35	1.70	ND
	iPR	220.17	22.62	149.02	32.71	105.67	26.38	133.81	50.74
Auxins	IAA	87.56	174.55	130.75	181.39	134.61	102.40	145.66	191.95
	IAA-Asp	171.57	46.59	84.02	13.33	95.34	47.98	181.67	29.20
Gibberellins	GA1	ND	ND	ND	ND	ND	ND	9.65	ND
	GA3	3.90	4.00	8.45	ND	ND	ND	ND	ND
	GA7	ND	ND	3.90	ND	7.40	5.90	3.95	ND
	GA8	29.04	6.05	40.81	4.76	13.29	5.95	55.02	4.90
	GA19	66.54	131.06	83.96	129.10	94.89	45.97	94.30	108.96
	GA29	29.10	23.64	24.96	16.15	26.20	18.37	22.18	13.67
	GA34	ND	ND	ND	3.70	ND	ND	ND	4.00
	GA53	3.90	8.28	4.28	9.45	7.40	5.60	4.27	6.33

Table S7. List of significantly affected ABA-related genes fold-changes from well-drained control plants

		Genotypes				
**Gene name**	**Gene ID**	**Wild-type**	**AC-Caribou**	**AAC-Trueman**	**miROE (A8)**	**SPL13RNAi-6**
PYL9	Medtr3g090980		1.468333339	1.940415079	1.313445512	1.610158038
	Medtr8g027805		1.480644153			1.361084937
	Medtr1g028380	4.963729546	16.94250381	4.410731563	8.915831456	10.64340389
PYR1	Medtr5g030500		0.551276568			0.631988256
ABA-receptor	Medtr7g070050		0.288305364			0.668773116
	Medtr1g016480	1.726137604	2.34850122	2.170072974	1.451561869	2.76464643
	Medtr5g083270		0.284645612	2.802468962	2.302822132	
	Medtr4g014460		0.386710299	2.074601331		
ABA induced bHLH	Medtr8g027495		0.44730769		0.627963112	0.681350829
	Medtr7g117670	0.578658563	0.504039594		0.609460569	0.662601162
	Medtr7g096350				1.353689034	
ABA/WDS Induced protein	Medtr1g098680	0.509854126	0.45983657	0.456389774	0.558366393	0.664104756
XDH1	Medtr2g098030	1.708143437	1.753770481	2.182169255	1.587031141	1.952980022
zeta-Carotene isomerase	Medtr3g084950					0.621969659
	Medtr8g097190			2.35743112		
Beta-Carotene Isomerase	Medtr7g095920					0.375806719
ABA-responsive protein	Medtr2g035190	4.476207756	8.025441393			
ABA-responsive (TB2/DP1, HVA22) family protein	Medtr1g024930	0.28821008	0.200650313	0.319513604	0.643970772	0.208526515
ABA response element-binding factor	Medtr4g085910	2.105747729	3.727460438	3.134374429	1.783055573	3.572943355
ABA-responsive-like protein	Medtr8g100065			1.788690853		1.77213165
	Medtr1g114640				2.661306098	
	Medtr7g083570			2.125450176	1.697723951	
	Medtr6g077990		4.409943923			
	Medtr8g100065		1.574540283			
ABA induced plasma membrane protein	Medtr4g117600	0.450860822				0.379373122
PSY	Medtr5g076620					0.652151143
	Medtr3g450510	0.411986337	0.227360821		0.705058323	0.299838299
	Medtr5g090780	0.455543133	0.274667972		0.575679144	0.194748945
PDS	Medtr3g084830		1.482103143	1.526032664		1.377339249
LCY-e	Medtr2g040060	0.27241664	0.332762581	0.574271942	0.563130472	0.278468961
LCY-b	Medtr7g090150		0.688293753			
	Medtr8g465870			1.697438637		
CHY	Medtr1g100070	0.368081354	0.241712735	0.608144239	0.667732718	0.248853736
	Medtr6g048440		1.489852257			
CCD1	Medtr3g109610		4.074932125	25.13738121		20.97763582
	Medtr8g037315			1.915333133	1.366102418	
CCD4	Medtr5g025270	0.246985367	0.283622967	0.172140204	0.405135074	0.428300805
	Medtr5g025230	0.289981861	0.338459135	0.184447256	0.433233598	0.40545407
ZEP	Medtr4g022850	4.03297979	7.705559819	2.67311853	2.210951765	6.096039107
	Medtr5g017350	3.03308126	3.232068708	2.438581712	2.083075313	5.613112534
VDE	Medtr8g092050		0.618185962			0.62706794
	Medtr7g116630	0.568931294	0.561403722		0.708757417	0.532251204
NSY	Medtr7g007280	0.459825004	0.340076773	0.348227281	0.651375774	0.346068728
	Medtr6g025680					3.126790208
	Medtr6g018550	4.045066625				
NCED3	Medtr2g070460	1.852418586	6.703907087	4.751628254	1.628771003	6.492220275
Cytochrome P450 family ABA- 8 hydroxylase	Medtr5g025610	0.518542824			0.618119458	1.890076358
	Medtr1g019410	3.375806092	11.55971202	5.120935372	2.245986502	6.127579653
	Medtr4g086040		0.551399468	0.584319511	0.59505537	0.516336322
	Medtr8g072260	0.278184042	0.209947451		1.809095309	0.615951388
	Medtr1g037580			0.49768187	1.835384513	
sucrose nonfermenting 1(SNF1)-related kinase	Medtr1g034030	2.780324005	2.711466533	2.343780998	1.778232373	3.525850452
	Medtr8g079560		1.916094332	1.743409131		1.705262408
	Medtr4g099240		1.790565027	1.619925849	1.454628246	
	Medtr5g018050				1.424120656	
SNF1-related kinase regulatory subunit beta-2	Medtr5g098510	6.884094008	9.97537076	7.161091135	6.328153516	10.14232325
	Medtr2g095290	1.921102765	2.2018862	1.848043183	1.541646047	1.703699503
	Medtr2g026695	0.600141087	0.535881509	0.510054311		0.578046214
SNF1-related kinase catalytic subunit alpha KIN11	Medtr6g048250		2.103369823			1.648765123
	Medtr6g012990		1.844837154			1.357621649
SNF1-related kinase regulatory subunit gamma 1	Medtr4g103550		3.003721173			1.564202386
Snf1-related kinase interactor 1, putative	Medtr4g007700		2.444608079	1.97123124		2.363397456
SNF2 family amino-terminal protein	Medtr4g118720		1.585782026			1.591472075
	Medtr7g050445	0.27928081	0.186708251			0.14658971
SNF2, helicase and zinc finger protein	Medtr2g012830	1.541181957	2.142240877	1.697455189	1.340032077	1.643170672

Table S8. DEG associated with phenylpropanoid pathway fold change in SPL13RNAi plants

Control	Flooded	Fold change	Gene name	Gene ID	Gene annotation
0.157259	1.76181	11.20	CHS	Medtr7g016700	chalcone and stilbene synthase family protein
0.863899	68.4215	79.20	CHS	Medtr3g083910	chalcone and stilbene synthase family protein
1.13824	19.3975	17.04	CHS	Medtr5g007713	chalcone and stilbene synthase family protein
0.853317	3.76625	4.41	CHS	Medtr1g097935	chalcone and stilbene synthase family protein
2.46825	9.57545	3.88	CHS	Medtr3g083920	chalcone and stilbene synthase family protein
0.184781	0.580756	3.14	CHS	Medtr1g097910	chalcone and stilbene synthase family protein
0.43232	1.1666	2.70	CHS	Medtr1g098140	chalcone and stilbene synthase family protein
281.474	496.147	1.76	CHS	Medtr7g084300	chalcone and stilbene synthase family protein
132.399	225.551	1.70	CHI	Medtr3g093980	chalcone-flavanone isomerase family protein
24.5176	15.9105	0.65	CHI	Medtr2g072510	chalcone-flavanone isomerase family protein
39.1902	20.4897	0.52	CHI	Medtr7g094980	chalcone-flavanone isomerase family protein
12.245	4.41102	0.36	CHI	Medtr1g115840	chalcone-flavanone isomerase family protein
28.1494	2.50074	0.09	CHI	Medtr8g075950	chalcone-flavanone isomerase family protein
8.46837	1.74168	0.21	CHI	Medtr5g022010	chalcone-flavanone isomerase family protein, putative
4.43879	170.01	38.30	LDOX	Medtr5g011250	leucoanthocyanidin dioxygenase-like protein
26.495	17.8889	0.68	LDOX	Medtr4g015790	leucoanthocyanidin dioxygenase-like protein
15.7654	5.08904	0.32	LDOX	Medtr3g070860	leucoanthocyanidin dioxygenase-like protein
20.2361	3.21226	0.16	LDOX	Medtr7g068650	leucoanthocyanidin dioxygenase-like protein
0.818886	29.0492	35.47	F3H	Medtr8g075830	naringenin 3-dioxygenase (flavanone-3-hydroxylase)
10.4318	6.88297	0.66	F3H	Medtr3g058610	naringenin 3-dioxygenase (flavanone-3-hydroxylase)
1.39805	30.1875	21.59	FLS	Medtr3g091350	flavonol synthase/flavanone 3-hydroxylase
0.353812	6.74808	19.07	FLS	Medtr5g055680	flavonol synthase/flavanone 3-hydroxylase
2.22346	10.4189	4.69	FLS	Medtr5g055690	flavonol synthase/flavanone 3-hydroxylase
7.03461	15.7578	2.24	FLS	Medtr5g059130	flavonol synthase/flavanone 3-hydroxylase
8.76692	12.9497	1.48	FLS	Medtr5g048850	flavonol synthase/flavanone 3-hydroxylase
23.4646	12.9946	0.55	FLS	Medtr5g065010	flavonol synthase/flavanone 3-hydroxylase
0.132358	14.2242	107.47	HID	Medtr3g450680	2-hydroxyisoflavanone dehydratase
0.474388	1.42907	3.01	HID	Medtr7g051020	2-hydroxyisoflavanone dehydratase
10.8869	22.6469	2.08	HID	Medtr1g104930	2-hydroxyisoflavanone dehydratase
0.672481	7.71948	11.48	IFR	Medtr4g070340	allergenic isoflavone reductase-like protein Bet protein
0.117758	0.857928	7.29	IFR	Medtr5g020760	isoflavone reductase-like protein
11.8543	71.9415	6.07	DFR	Medtr1g022440	dihydroflavonol 4-reductase-like protein
6.69756	3.9136	0.58	DFR	Medtr2g013250	dihydroflavonol-4-reductase-like protein
0.757691	41.0798	54.22	F3,5H	Medtr4g109470	flavonoid hydroxylase
0.046738	0.740585	15.85	F3,5H	Medtr3g024520	flavonoid hydroxylase
4.49419	23.6299	5.26	FGT	Medtr3g084520	flavonoid glucosyltransferase
6.48035	16.63	2.57	FGT	Medtr3g084530	flavonoid glucosyltransferase
15.9971	61.729	3.86	FGT	Medtr4g128690	flavonoid glucosyltransferase
80.5089	10.2809	0.13	FGT	Medtr4g485630	flavonoid glucosyltransferase
72.6701	5.51536	0.08	FGT	Medtr4g031800	flavonoid glucosyltransferase
4.47777	0.841314	0.19	FOMT	Medtr3g083620	flavonoid O-methyltransferase-like protein
0.168525	0.587252	3.48	AAT	Medtr2g089650	anthocyanin 5-aromatic acyltransferase
15.963	36.3294	2.28		Medtr7g014160	anthocyanin 5-aromatic acyltransferase
24.5972	53.9455	2.19		Medtr4g015180	anthocyanin 5-aromatic acyltransferase
26.359	17.3893	0.66		Medtr7g014205	anthocyanin 5-aromatic acyltransferase
6.63988	2.36659	0.36		Medtr7g015860	anthocyanin 5-aromatic acyltransferase
14.5124	1.5601	0.11		Medtr2g089670	anthocyanin 5-aromatic acyltransferase
84.2219	60.3209	0.72		Medtr2g018590	1-O-acylglucose:anthocyanin acyltransferase
0.606445	1.25578	2.07	PAL	Medtr5g098720	phenylalanine ammonia-lyase-like protein
17.7626	10.6909	0.60	PAL	Medtr7g101395	phenylalanine ammonia-lyase-like protein
21.4047	12.6183	0.59	PAL	Medtr7g101425	phenylalanine ammonia-lyase-like protein
0.514266	0.0833833	0.16		Medtr2g064495	shikimate/quinate hydroxycinnamoyltransferase
11.9181	21.1793	1.78	CCR	Medtr4g009040	cinnamoyl-CoA reductase
37.5393	75.3853	2.01	CCRL	Medtr5g072620	cinnamoyl-CoA reductase-like protein
522.729	888.859	1.70	CCRL	Medtr3g031650	cinnamoyl-CoA reductase-like protein
23.5755	11.8414	0.50	CCRL	Medtr4g077100	cinnamoyl-CoA reductase-like protein
14.717	6.24377	0.42	CCRL	Medtr4g006940	cinnamoyl-CoA reductase-like protein
71.2782	142.814	2.00	CADL	Medtr1g107425	cinnamyl alcohol dehydrogenase-like protein
49.2539	77.2787	1.57	CADL	Medtr3g005170	cinnamyl-alcohol dehydrogenase-like protein
12.3828	18.3453	1.48	CADL	Medtr5g031180	cinnamyl alcohol dehydrogenase-like protein
11.4937	4.75367	0.41	CADL	Medtr4g115300	cinnamyl alcohol dehydrogenase-like protein
101.33	37.7547	0.37	CADL	Medtr1g025950	cinnamyl alcohol dehydrogenase-like protein
1.01223	0.364236	0.36	CADL	Medtr5g031430	cinnamyl alcohol dehydrogenase-like protein
240.397	59.0809	0.25	CADL	Medtr5g031190	cinnamyl alcohol dehydrogenase-like protein
3.15267	8.28627	2.63	4CL	Medtr1g073180	4-coumarate:CoA ligase-like protein
1.36185	9.28901	6.82	4CL	Medtr4g057730	4-coumarate:CoA ligase-like protein
11.3113	17.619	1.56	4CL	Medtr5g007640	4-coumarate:CoA ligase-like protein
42.3478	57.6463	1.36	4CL	Medtr4g128337	4-coumarate:CoA ligase-like protein
31.8462	19.3249	0.61	4CL	Medtr4g005750	4-coumarate:CoA ligase-like protein
110.624	66.279	0.60	4CL	Medtr2g105570	4-coumarate:CoA ligase-like protein
0.365712	0.0424932	0.12	4CL	Medtr1g063110	4-coumarate:CoA ligase-like protein
13.5551	22.704	1.67		Medtr2g024310	malonyl-CoA decarboxylase
1.81981	27.6218	15.18	MCD	Medtr6g015830	malonyl-CoA:isoflavone 7-O-glucoside malonyltransferase
0.116006	0.454496	3.92		Medtr6g015315	malonyl-CoA:isoflavone 7-O-glucoside malonyltransferase
0.937934	2.29464	2.45		Medtr6g015320	malonyl-CoA:isoflavone 7-O-glucoside malonyltransferase
6.86031	2.93338	0.43		Medtr3g031380	malonyl CoA-acyl carrier transacylase
1.70143	5.00687	2.94	FNS II	Medtr4g088160	cytochrome P450 family flavone synthase
0.137557	0.919824	6.69	UGT	Medtr8g059425	UDP-glucosyltransferase family protein
0.350648	1.66457	4.75		Medtr2g040530	UDP-glucosyltransferase family protein
196.293	928.746	4.73		Medtr3g479470	UDP-glucosyltransferase family protein
5.33138	22.235	4.17		Medtr7g102560	UDP-glucosyltransferase family protein
1.2037	4.83554	4.02		Medtr2g083430	UDP-glucosyltransferase family protein
0.158481	0.615249	3.88		Medtr7g071050	UDP-glucosyltransferase family protein
2.68234	8.55554	3.19		Medtr4g064987	UDP-glucosyltransferase family protein
2.94393	9.27543	3.15		Medtr7g013200	UDP-glucosyltransferase family protein
4.52185	13.4526	2.98		Medtr3g009490	UDP-glucosyltransferase family protein
13.5481	32.2392	2.38		Medtr7g046490	UDP-glucosyltransferase family protein
2.79832	6.62039	2.37		Medtr6g014270	UDP-glucosyltransferase family protein
2.21141	4.91576	2.22		Medtr2g059240	UDP-glucosyltransferase family protein
0.697333	1.46894	2.11		Medtr0184s0030	UDP-glucosyltransferase family protein
15.7726	32.9981	2.09		Medtr5g070040	UDP-glucosyltransferase family protein
31.7106	63.4933	2.00		Medtr8g006730	UDP-glucosyltransferase family protein
0.52214	1.03202	1.98		Medtr8g466240	UDP-glucosyltransferase family protein
8.72568	17.0294	1.95		Medtr8g006710	UDP-glucosyltransferase family protein
140.744	263.264	1.87		Medtr7g117170	UDP-glucosyltransferase family protein
2.13248	3.63389	1.70		Medtr8g071070	UDP-glucosyltransferase family protein
4.84927	7.40883	1.53		Medtr7g013180	UDP-glucosyltransferase family protein
30.7221	43.9453	1.43		Medtr4g123553	UDP-glucosyltransferase family protein
69.8163	46.0608	0.66		Medtr5g067170	UDP-glucosyltransferase family protein
7.21762	4.68116	0.65		Medtr5g072860	UDP-glucosyltransferase family protein
37.1886	23.8406	0.64		Medtr2g046620	UDP-glucosyltransferase family protein
16.2584	9.52955	0.59		Medtr5g029800	UDP-glucosyltransferase family protein
6.43999	3.7057	0.58		Medtr8g009055	UDP-glucosyltransferase family protein
29.8741	16.7492	0.56		Medtr8g027870	UDP-glucosyltransferase family protein
67.8708	36.7269	0.54		Medtr0078s0010	UDP-glucosyltransferase family protein
7.91155	4.20799	0.53		Medtr3g008010	UDP-glucosyltransferase family protein
1.78859	0.948812	0.53		Medtr7g047030	UDP-glucosyltransferase family protein
6.07381	2.92499	0.48		Medtr7g102490	UDP-glucosyltransferase family protein
14.6454	6.9002	0.47		Medtr6g042310	UDP-glucosyltransferase family protein
3.31818	1.54762	0.47		Medtr2g083490	UDP-glucosyltransferase family protein
2.77768	1.23336	0.44		Medtr4g024020	UDP-glucosyltransferase family protein
11.0104	4.84754	0.44		Medtr0536s0010	UDP-glucosyltransferase family protein
9.06752	3.95663	0.44		Medtr6g014250	UDP-glucosyltransferase family protein
1.31569	0.542235	0.41		Medtr4g077960	UDP-glucosyltransferase family protein
2.46584	0.97921	0.40		Medtr1g107325	UDP-glucosyltransferase family protein
18.3859	7.18924	0.39		Medtr6g035295	UDP-glucosyltransferase family protein
0.946443	0.358983	0.38		Medtr6g005600	UDP-glucosyltransferase family protein
8.7515	3.25315	0.37		Medtr8g008970	UDP-glucosyltransferase family protein
7.1097	2.47922	0.35		Medtr8g068330	UDP-glucosyltransferase family protein
7.86048	2.56268	0.33		Medtr2g008210	UDP-glucosyltransferase family protein

3.6433	1.16332	0.32		Medtr2g083380	UDP-glucosyltransferase family protein
1.48236	0.468547	0.32		Medtr6g038300	UDP-glucosyltransferase family protein
3.39875	1.00155	0.29		Medtr8g006260	UDP-glucosyltransferase family protein
3.9818	1.13242	0.28		Medtr7g012120	UDP-glucosyltransferase family protein
3.986	0.829804	0.21		Medtr3g069690	UDP-glucosyltransferase family protein
7.38548	1.46819	0.20		Medtr8g068340	UDP-glucosyltransferase family protein
59.3483	11.625	0.20		Medtr5g035560	UDP-glucosyltransferase family protein
32.2985	6.01397	0.19		Medtr5g016660	UDP-glucosyltransferase family protein
37.4396	6.72904	0.18		Medtr2g034990	UDP-glucosyltransferase family protein
19.8098	3.47176	0.18		Medtr4g117890	UDP-glucosyltransferase family protein
0.999749	0.167898	0.17		Medtr0891s0010	UDP-glucosyltransferase family protein
5.80386	0.9703	0.17		Medtr7g055710	UDP-glucosyltransferase family protein
6.41592	0.879345	0.14		Medtr6g078320	UDP-glucosyltransferase family protein
10.7019	1.42278	0.13		Medtr2g008226	UDP-glucosyltransferase family protein
1.52347	0.202059	0.13		Medtr4g059370	UDP-glucosyltransferase family protein
25.4363	3.31422	0.13		Medtr8g044140	UDP-glucosyltransferase family protein
98.5232	6.06681	0.06		Medtr2g008220	UDP-glucosyltransferase family protein
2.63237	0.0364904	0.01		Medtr5g075440	UDP-glucosyltransferase family protein
12.0424	45.3894	3.77		Medtr6g033675	UDP-glucosyl transferase 88A1
0.367654	0.987389	2.69		Medtr6g033725	UDP-glucosyl transferase 88A1
33.3904	61.4334	1.84		Medtr4g485640	UDP-glucosyltransferase 73B2
115.392	191.329	1.66		Medtr1g088480	UDP-glucuronic acid decarboxylase-like protein
10.4702	6.57739	0.63		Medtr3g095910	UDP-glucuronate:xylan alpha-glucuronosyltransferase
3.85051	2.30533	0.60		Medtr1g019530	UDP-glucuronate:xylan alpha-glucuronosyltransferase
33.2139	54.163	1.63	UF3GT	Medtr5g090580	UDP-glucose flavonoid 3-O-glucosyltransferase
9.65266	5.39069	0.56		Medtr5g098930	UDP-glucose flavonoid 3-O-glucosyltransferase
19.8753	31.1709	1.57		Medtr7g076310	UDP-galactose transporter 1
11.4065	16.2493	1.42		Medtr1g098640	UDP-glucose:sterol glucosyltransferase
16.6317	11.0046	0.66		Medtr8g446850	UDP-glcnac-adolichol phosphate glcnac-1-P-transferase
121.813	59.2811	0.49		Medtr5g043970	UDP-glucose 6-dehydrogenase family protein
132.319	63.7785	0.48		Medtr7g012950	UDP-glucose 6-dehydrogenase family protein
71.2601	38.6445	0.54		Medtr2g096660	UDP-glucuronic acid decarboxylase
0.120398	8.52013	70.77	MYB	Medtr5g079670	myb transcription factor
0.432767	24.86	57.44	MYB	Medtr1g086510	myb transcription factor
0.230764	4.84289	20.99	MYB	Medtr2g033170	myb transcription factor
0.079506	1.51186	19.02	MYB	Medtr1g110460	myb transcription factor
0.550735	6.25846	11.36	MYB	Medtr4g123040	myb transcription factor
35.8314	237.333	6.62	MYB	Medtr2g096380	myb transcription factor
0.530137	3.12412	5.89	MYB	Medtr3g083540	myb transcription factor
0.124816	0.669176	5.36	MYB	Medtr4g057635	myb transcription factor
49.1599	204.177	4.15	MYB	Medtr5g016510	myb transcription factor
0.17398	0.682151	3.92	MYB	Medtr5g009460	myb transcription factor
14.7859	65.1316	4.40	MYB	Medtr4g094982	myb transcription factor
0.576214	1.48385	2.58	MYB	Medtr4g100720	myb transcription factor
1.11895	3.08061	2.75	MYB	Medtr2g011660	myb transcription factor
84.5599	229.053	2.71	MYB	Medtr4g063100	myb transcription factor
12.2639	27.9597	2.28	MYB	Medtr7g087130	myb transcription factor
7.45783	14.8842	2.00	MYB	Medtr8g028655	myb transcription factor
28.7948	54.8752	1.91	MYB	Medtr0123s0070	myb transcription factor
2.53256	4.33654	1.71	MYB	Medtr8g095390	myb transcription factor
10.7008	16.371	1.53	MYB	Medtr3g064500	myb transcription factor
16.1631	23.335	1.44	MYB	Medtr8g020330	myb transcription factor
170.172	127.593	0.75	MYB	Medtr1g067000	myb transcription factor
44.9463	28.9646	0.64	MYB	Medtr6g477860	myb transcription factor
12.8452	7.70599	0.60	MYB	Medtr4g102380	myb transcription factor
3.35322	1.9876	0.59	MYB	Medtr4g019370	myb transcription factor
1.30247	0.703915	0.54	MYB	Medtr3g028740	myb transcription factor
23.5059	12.6929	0.54	MYB	Medtr4g073420	myb transcription factor
41.6862	20.7641	0.50	MYB	Medtr1g063940	myb transcription factor
4.51852	2.17317	0.48	MYB	Medtr8g098860	myb transcription factor
5.75459	2.70077	0.47	MYB	Medtr4g065017	myb transcription factor
2.00004	0.869425	0.43	MYB	Medtr8g468380	myb transcription factor
1.60222	0.661824	0.41	MYB	Medtr2g097910	myb transcription factor
17.9481	6.43035	0.36	MYB	Medtr7g061550	myb transcription factor
1.89876	0.67792	0.36	MYB	Medtr5g078140	myb transcription factor
1.14311	0.406191	0.36	MYB	Medtr7g096930	myb transcription factor
2.11574	0.639308	0.30	MYB	Medtr1g085040	myb transcription factor
0.640971	0.148424	0.23	MYB	Medtr7g117730	myb transcription factor
1.96408	0.408359	0.21	MYB	Medtr7g109320	myb transcription factor
0.699955	0.144624	0.21	MYB	Medtr5g082910	myb transcription factor
0.844207	0.0969873	0.11	MYB	Medtr4g082040	myb transcription factor
15.173	0.817636	0.05	MYB	Medtr4g121460	myb transcription factor
5.80651	0.277499	0.05	MYB	Medtr2g034790	myb transcription factor
108.368	3.02779	0.03	MYB	Medtr2g023100	myb transcription factor
4.71002	2.80708	0.60	MYB	Medtr1g086180	MYB family transcription factor
27.6694	16.3007	0.59	MYB	Medtr1g022290	MYB family transcription factor
314.802	52.2153	0.17	MYB	Medtr8g077420	MYB family transcription factor
1.7983	0.197476	0.11	MYB	Medtr3g111920	MYB family transcription factor
12.7126	0.980649	0.08	MYB	Medtr3g111880	MYB family transcription factor
56.0058	3.2274	0.06	MYB	Medtr7g089210	MYB family transcription factor
35.2036	0.969802	0.03	MYB	Medtr8g077390	MYB family transcription factor
10.8536	3.60458	0.33	MYB5	Medtr1g026870	transcription repressor MYB5
11.7042	1.78489	0.15	MYB5	Medtr3g110028	transcription repressor MYB5
17.2453	26.0005	1.51	MYB51	Medtr5g081860	MYB transcription factor MYB51
17.6979	25.733	1.45	MYB51	Medtr7g067080	MYB transcription factor MYB51
11.7448	16.7709	1.43	MYB51	Medtr3g462790	MYB transcription factor MYB51
7.93977	2.27366	0.29	MYB64	Medtr7g110830	myb transcription factor MYB64
44.9035	203.915	4.54	MYB	Medtr5g037080	MYB-like transcription factor family protein
15.7197	59.8805	3.81	MYB	Medtr4g081710	myb-like transcription factor family protein
11.0827	29.8953	2.70	MYB	Medtr2g086450	myb-like transcription factor family protein
7.66715	17.6767	2.31	MYB	Medtr1g111830	MYB-like transcription factor family protein
23.2199	53.217	2.29	MYB	Medtr7g115530	myb-like transcription factor family protein
31.2609	67.6386	2.16	MYB	Medtr6g444980	myb-like transcription factor family protein
1.65447	3.50801	2.12	MYB	Medtr8g086410	MYB-like transcription factor family protein
10.4112	21.8071	2.09	MYB	Medtr6g032990	myb-like transcription factor family protein
0.566713	1.15656	2.04	MYB	Medtr1g053835	myb-like transcription factor family protein
0.952566	1.92501	2.02	MYB	Medtr7g069660	MYB-like transcription factor family protein
4.61434	9.18372	1.99	MYB	Medtr4g113140	myb-like transcription factor family protein
37.7677	65.5914	1.74	MYB	Medtr7g088070	myb-like transcription factor family protein
21.7583	30.0845	1.38	MYB	Medtr2g027860	myb-like transcription factor family protein
33.209	45.1914	1.36	MYB	Medtr7g098250	myb-like transcription factor family protein
13.4695	8.48777	0.63	MYB	Medtr4g086835	myb-like transcription factor family protein
25.1176	15.6944	0.62	MYB	Medtr4g111975	MYB-like transcription factor family protein
20.6778	11.8119	0.57	MYB	Medtr1g090670	myb-like transcription factor family protein
2.04156	0.999157	0.49	MYB	Medtr1g093080	myb-like transcription factor family protein
24.6511	17.4084	0.71	MYB	Medtr8g077990	Myb transcription factor-like protein
0.196743	2.14551	10.91	Myb/SANT	Medtr7g105890	Myb/SANT-like DNA-binding domain protein
1.21298	8.49433	7.00	Myb/SANT	Medtr4g128540	Myb/SANT-like DNA-binding domain protein
1.79056	11.6691	6.52	Myb/SANT	Medtr0693s0050	Myb/SANT-like DNA-binding domain protein
0.084772	0.470761	5.55	Myb/SANT	Medtr1g016370	Myb/SANT-like DNA-binding domain protein
1.64831	5.59186	3.39	Myb/SANT	Medtr7g020870	myb/SANT-like DNA-binding domain protein
4.24087	2.5629	0.60	Myb/SANT	Medtr2g026725	Myb/SANT-like DNA-binding domain protein
21.9766	7.86686	0.36	Myb/SANT	Medtr6g035370	myb/SANT-like DNA-binding domain protein
1.27208	0.355906	0.28	Myb/SANT	Medtr1g061640	Myb/SANT-like DNA-binding domain protein
6.90884	1.26822	0.18	Myb/SANT	Medtr7g114860	Myb/SANT-like DNA-binding domain protein
0.64763	0.094147	0.15	Myb/SANT	Medtr8g085310	Myb/SANT-like DNA-binding domain protein
1.5387	0.156707	0.10	Myb/SANT	Medtr1g094045	Myb/SANT-like DNA-binding domain protein
0.179537	3.0709	17.10	R2R3-myb	Medtr5g078860	R2R3-myb transcription factor
0.704362	11.7703	16.71	R2R3-myb	Medtr5g078910	R2R3-myb transcription factor
8.07993	4.9244	0.61	R2R3-myb	Medtr6g012180	R2R3-myb transcription factor
1.58705	0.736946	0.46	R2R3-myb	Medtr3g039990	R2R3-myb transcription factor
4.21645	1.91166	0.45	R2R3-myb	Medtr0140s0030	R2R3-myb transcription factor

8.00167	13.9304	1.74		Medtr3g100180	myb-like DNA-binding domain protein
0.16105	0.659837	4.10		Medtr2g084230	myb-like DNA-binding domain, shaqkyf class protein
0.837565	2.54891	3.04		Medtr3g450310	myb-like DNA-binding domain, shaqkyf class protein
3.52187	5.91887	1.68		Medtr6g071625	myb-like DNA-binding domain, shaqkyf class protein
1.65367	0.882482	0.53		Medtr1g112370	myb-like DNA-binding domain, shaqkyf class protein
0.49824	0.159056	0.32		Medtr2g016220	myb-like DNA-binding domain, shaqkyf class protein
4.28008	1.24876	0.29		Medtr5g017980	myb-like DNA-binding domain, shaqkyf class protein
3.0424	0.617461	0.20		Medtr0223s0040	myb-like DNA-binding domain, shaqkyf class protein
0.987074	2.32545	2.36		Medtr1g100653	myb transcription factor MIXTA-like protein
8.20835	3.35286	0.41		Medtr7g011170	myb transcription factor mixta-like protein
18.5249	5.89435	0.32		Medtr6g012690	myb transcription factor mixta-like protein
1.45991	0.333044	0.23		Medtr8g031360	myb transcription factor MIXTA-like protein
0.11246	1.37871	12.26	LBM1	Medtr1g043080	myb-related transcription factor LBM1
0.158576	9.95577	62.78	WD40	Medtr1g021590	transducin/WD40 repeat protein
48.1375	120.34	2.50	WD40	Medtr3g074070	transducin/WD40 repeat protein
8.21249	17.562	2.14	WD40	Medtr8g073335	transducin/WD40 repeat protein
4.25151	7.86745	1.85	WD40	Medtr3g491920	transducin/WD40 repeat protein
64.3551	102.99	1.60	WD40	Medtr3g010010	transducin/WD40 repeat protein
17.4037	24.994	1.44	WD40	Medtr7g114370	transducin/WD40 repeat protein
16.468	23.409	1.42	WD40	Medtr7g022190	transducin/WD40 repeat protein
19.8835	27.1718	1.37	WD40	Medtr7g074450	transducin/WD40 repeat protein
38.735	52.7972	1.36	WD40	Medtr2g028050	transducin/WD40 repeat protein
32.6727	44.437	1.36	WD40	Medtr5g090420	transducin/WD40 repeat protein
9.49086	6.52318	0.69	WD40	Medtr6g093060	transducin/WD40 repeat protein
7.25918	4.88408	0.67	WD40	Medtr7g016970	transducin/WD40 repeat protein
8.55168	5.67702	0.66	WD40	Medtr4g130280	transducin/WD40 repeat protein
10.9794	6.83287	0.62	WD40	Medtr4g105700	transducin/WD40 repeat protein
4.01492	2.42562	0.60	WD40	Medtr1g059090	transducin/WD40 repeat protein
9.03513	5.39885	0.60	WD40	Medtr1g081830	transducin/WD40 repeat protein
9.52219	4.53135	0.48	WD40	Medtr1g007740	transducin/WD40 repeat protein
1.61882	0.448949	0.28	WD40	Medtr5g077160	transducin/WD40 repeat protein
3.22979	0.535583	0.17	WD40	Medtr4g066000	transducin/WD40 domain protein
12.6325	8.58941	0.68		Medtr6g004040	katanin p80 WD40 repeat subunit B1-like protein
13.0724	17.4139	1.33		Medtr1g087120	WD40/YVTN repeat containing domain-containing protein
0.482652	9.73325	20.17	bHLH	Medtr1g072320	bHLH transcription factor
9.29101	16.4404	1.77	bHLH	Medtr5g048860	BHLH transcription factor
15.1871	26.152	1.72	bHLH	Medtr4g108360	BHLH transcription factor
27.9724	40.992	1.47	bHLH	Medtr4g131160	BHLH transcription factor
7.58623	2.33027	0.31	bHLH	Medtr3g498695	BHLH transcriptional factor
13.6047	1.83974	0.14	bHLH	Medtr3g116770	BHLH transcription factor
23.8017	90.6247	3.81	bHLH147	Medtr1g084160	transcription factor bHLH147
72.6744	52.8237	0.73	bHLH137	Medtr3g498825	transcription factor bHLH137
10.6712	4.15754	0.39	bHLH93	Medtr5g030770	transcription factor bHLH93
23.5912	49.2664	2.09	bHLH	Medtr7g072470	transcription factor bHLH93-like protein
0.481536	0.0864422	0.18	bHLH	Medtr4g079760	transcription factor bHLH107-like protein
40.0829	83.2713	2.08	bHLH	Medtr6g488100	BHLH transcription factor-like protein
19.4702	38.817	1.99	bHLH	Medtr8g103065	BHLH transcription factor-like protein
32.0128	61.6959	1.93	bHLH	Medtr2g101520	BHLH transcription factor-like protein
3.31696	6.02474	1.82	bHLH	Medtr7g063470	bHLH transcription factor-like protein
12.6943	18.2201	1.44	bHLH	Medtr7g096650	bHLH transcription factor-like protein
23.6729	32.3027	1.36	bHLH	Medtr4g051565	bHLH transcription factor-like protein
41.6825	27.7753	0.67	bHLH	Medtr4g087300	BHLH transcription factor-like protein
2.72595	0.94114	0.35	bHLH	Medtr3g112170	BHLH transcription factor-like protein
3.71394	6.28639	1.69	bHLH	Medtr1g052470	transcription factor bHLH122-like protein
24.3379	39.2204	1.61	bHLH	Medtr4g065870	transcription factor bHLH122-like protein
37.5145	65.5628	1.75	bHLH	Medtr1g070870	basic helix loop helix protein BHLH4
3.04939	17.4671	5.73	bHLH	Medtr8g099880	basic helix loop helix protein BHLH8
28.1758	15.0457	0.53	bHLH	Medtr7g083900	basic helix loop helix protein BHLH23
16.4588	23.4003	1.42	bHLH	Medtr1g043430	BHLH domain class transcription factor
22.6827	33.024	1.46	bHLH	Medtr5g030420	basic helix loop helix (BHLH) family transcription factor
16.5506	10.7003	0.65	bHLH	Medtr8g067280	basic helix loop helix (bHLH) family transcription factor
8.81645	4.70374	0.53	bHLH	Medtr8g024790	basic helix loop helix (bHLH) family transcription factor
0.217682	1.11105	5.10		Medtr4g094762	basic helix loop helix (BHLH) DNA-binding family protein
33.564	16.5108	0.49		Medtr5g014520	basic helix loop helix (bHLH) DNA-binding family protein
3.26047	1.4972	0.46		Medtr0246s0020	basic helix loop helix (bHLH) DNA-binding family protein
12.2783	5.43255	0.44		Medtr5g014600	basic helix loop helix (bHLH) DNA-binding family protein
13.8229	4.75374	0.34		Medtr1g093750	basic helix loop helix (BHLH) DNA-binding family protein
0.748242	0.230926	0.31		Medtr3g099620	basic helix loop helix (BHLH) DNA-binding family protein
1.96629	0.320185	0.16		Medtr2g022280	basic helix loop helix (BHLH) DNA-binding family protein
1.14533	2.07771	1.81	bZIP	Medtr4g012430	BZIP protein
10.7869	15.1678	1.41	bZIP	Medtr2g102050	BZIP protein
8.35676	1.01161	0.12	bZIP	Medtr3g109450	BZIP protein
0.075122	0.862346	11.48	bZIP	Medtr2g086340	bZIP transcription factor
0.724958	7.2286	9.97	bZIP	Medtr3g109310	BZIP transcription factor
16.4438	64.7385	3.94	bZIP	Medtr1g008990	BZIP transcription factor
7.33424	15.2449	2.08	bZIP	Medtr1g098590	BZIP transcription factor
7.47561	15.3233	2.05	bZIP	Medtr3g078830	BZIP transcription factor
11.0567	22.0465	1.99	bZIP	Medtr6g034945	bZIP transcription factor
47.7718	83.2262	1.74	bZIP	Medtr5g038550	BZIP transcription factor
18.1531	30.1794	1.66	bZIP	Medtr1g023690	BZIP transcription factor
18.6275	28.3531	1.52	bZIP	Medtr2g099050	bZIP transcription factor
14.4279	21.0393	1.46	bZIP	Medtr3g436010	BZIP transcription factor
3.15742	2.04794	0.65	bZIP	Medtr8g091250	BZIP transcription factor
16.3044	10.3419	0.63	bZIP	Medtr4g072090	BZIP transcription factor
18.4019	9.12756	0.50	bZIP	Medtr3g094020	bZIP transcription factor
18.2584	6.23088	0.34	bZIP	Medtr5g075390	bZIP transcription factor
5.32053	1.69353	0.32	bZIP	Medtr5g087740	BZIP transcription factor
14.6869	4.54229	0.31	bZIP	Medtr3g467120	bZIP transcription factor
1.18751	0.35949	0.30	bZIP	Medtr8g075130	BZIP transcription factor
2.10162	0.368046	0.18	bZIP	Medtr6g016375	BZIP transcription factor
13.3568	22.6691	1.70	bZIP	Medtr8g091650	BZIP family transcription factor
39.3776	61.8403	1.57	bZIP	Medtr8g040120	BZIP family transcription factor
0.171638	0.62132	3.62	bZIP	Medtr7g092750	BZIP family transcription factor
7.1997	24.1693	3.36	bZIP	Medtr5g060950	BZIP family transcription factor
0.06184	0.346624	5.61	bZIP	Medtr0126s0080	BZIP family transcription factor
0.267177	1.28501	4.81	bZIP	Medtr2g086420	BZIP family transcription factor
20.1294	1.62464	0.08	bZIP	Medtr4g079500	bZIP family transcription factor
8.44693	0.614546	0.07	bZIP	Medtr2g090900	bZIP family transcription factor
67.0252	375.604	5.60	bZIP124	Medtr4g070860	BZIP transcription factor bZIP124
30.9631	116.475	3.76	bZIP124	Medtr3g117120	BZIP transcription factor bZIP124
26.0435	17.6029	0.68	bZIP80	Medtr7g081705	BZIP transcription factor bZIP80
14.249	2.38044	0.17	bZIP88	Medtr7g115120	transcription factor bZIP88
3.84622	11.8769	3.09	bZIP	Medtr8g028150	bZIP transcription factor family protein
41.7014	84.3009	2.02	bZIP	Medtr7g116890	bZIP transcription factor family protein
33.7726	24.8979	0.74	bZIP	Medtr8g077950	bZIP transcription factor family protein
0.463323	4.68381	10.11	GST	Medtr6g080440	glutathione S-transferase
0.973524	13.4098	13.77	GST	Medtr1g115195	glutathione S-transferase
50.4936	100.29	1.99	GST	Medtr1g067170	glutathione S-transferase
26.1468	48.6235	1.86	GST	Medtr5g090910	glutathione S-transferase
1.63306	3.01346	1.85	GST	Medtr7g100320	glutathione S-transferase
40.9017	57.9545	1.42	GST	Medtr3g450790	glutathione S-transferase
369.912	105.272	0.28	GST	Medtr1g115500	glutathione S-transferase
25.7497	14.2299	0.55		Medtr2g072120	glutathione S-transferase tau
15.8005	53.8127	3.41	GSTU5	Medtr4g134380	glutathione S-transferase tau 5
13.6054	30.2248	2.22		Medtr4g134370	glutathione S-transferase tau 5
0.989109	12.1507	12.28		Medtr1g090150	glutathione S-transferase, amino-terminal domain protein
8.03876	458.188	57.00		Medtr3g064700	glutathione S-transferase, amino-terminal domain protein
0.903146	21.6331	23.95		Medtr1g090060	glutathione S-transferase, amino-terminal domain protein
3.50749	31.068	8.86		Medtr7g065265	glutathione S-transferase, amino-terminal domain protein
3.79018	24.5397	6.47		Medtr7g065230	glutathione S-transferase, amino-terminal domain protein
7.73919	44.9179	5.80		Medtr2g070070	glutathione S-transferase, amino-terminal domain protein
6.26214	28.0683	4.48		Medtr3g099757	glutathione S-transferase, amino-terminal domain protein
0.207648	0.775324	3.73		Medtr2g070120	glutathione S-transferase, amino-terminal domain protein
0.419894	1.09915	2.62		Medtr3g467420	glutathione S-transferase, amino-terminal domain protein
3.59599	8.75853	2.44		Medtr8g061950	glutathione S-transferase, amino-terminal domain protein
13.3277	25.4855	1.91		Medtr3g088635	glutathione S-transferase, amino-terminal domain protein
4.09534	6.59916	1.61		Medtr4g019780	glutathione S-transferase, amino-terminal domain protein
23.5805	37.5887	1.59		Medtr1g059970	glutathione S-transferase, amino-terminal domain protein
80.0303	42.5945	0.53		Medtr3g066060	glutathione S-transferase, amino-terminal domain protein
1.80519	0.947428	0.52		Medtr3g005720	glutathione S-transferase, amino-terminal domain protein
22.2606	10.5267	0.47		Medtr2g070060	glutathione S-transferase, amino-terminal domain protein
3.49344	1.50116	0.43		Medtr8g098430	glutathione S-transferase, amino-terminal domain protein
19.2616	7.21202	0.37		Medtr1g026140	glutathione S-transferase, amino-terminal domain protein
9.42449	3.23428	0.34		Medtr2g070210	glutathione S-transferase, amino-terminal domain protein
64.2695	15.2161	0.24		Medtr5g037380	glutathione S-transferase, amino-terminal domain protein

Table S9. Coding sequencs of SPLs with conserved squamosa binding domain from different plants used for phylogenetic analysis

>AtSPL1

tgtcaggtcgaaaactgtgaagctgatcttagtaaagttaaggattatcatagacgccataaggtctgtgagatgcattccaaggctactagtgccactgtcggaggtatcttgcagcgcttttgtcagcaatgtagtaggttccatcttcttcaggagtttgatgaaggaaagagaagt
tgtcgtagacgtttggctggccataataaacgtcggaggaaaacaaat

>AtSPL2

tgtcaagttgaaggctgtaatcttgatctttcatcagctaaagactatcatcggaaacataggatttgtgaaaatcattcaaagtttcctaaagtcgttgtgagtggcgtagagcgtcggttctgccaacaatgtagcaggttccactgtctctctgagtttgatgagaagaaacgtagc
tgtcgccgacgtctctcagatcacaatgcaagacgtcgcaagccaaat

>AtSPL3

tgtcaggtcgagagttgtaccgcggatatgagcaaagccaaacagtaccacaaacgacacaaagtctgccagtttcatgccaaagctcctcatgttcggatctctggtcttcaccaacgtttctgccaacaatgcagcaggtttcacgcgctcagtgagtttgatgaagccaagcggagt
tgcaggagacgcttagctggacacaacgagagaaggcggaaaagcaca

>AtSPL4

tgccaagtagatagatgcacagctgatatgaaagaggcaaaactgtatcaccggagacacaaagtgtgtgaagttcatgcaaaggcatcttctgtctttctctcaggacttaaccaacgcttttgtcaacaatgcagtaggtttcatgacctccaagagtttgatgaagctaagagaagt
tgcaggaggcgcttagctggacacaatgagcgaagaaggaagagctct

>AtSPL5

tgccaggtcgataggtgcactgttaatttgactgaggccaagcagtattaccgcagacacagagtatgtgaagtacatgcaaaggcatctgctgcgactgttgcaggggtcaggcaacgcttttgtcaacaatgcagcaggtttcatgagctaccagagtttgatgaagctaaaagaagc
tgcaggaggcgcttagctggacacaatgagaggaggaggaagatctct

>AtSPL6

tgtcaagtttatgggtgtagtaaggatctgagctcttcgaaagattaccacaaaaggcatagagtttgcgaggctcattcgaaaacttctgtggtcatagttaatggtcttgaacagaggttttgtcaacagtgcagcaggtttcatttcctctcagagtttgatgatggcaaaagaagt
tgcagaaggcgattagccggtcacaatgaacgaagaaggaaacctgca

>AtSPL7

tgtcaggttccggattgtgaagcggatattagcgagctcaaagggtaccataagaggcatagggtttgtctccgttgcgctaccgccagctttgttgtgcttgatggagagaataagagatactgtcaacagtgtggaaagtttcatttgctcccggactttgatgaaggaaaacgcagc
tgtcggagaaagctagagcgtcacaacaacagacggaaaaggaaacct

>AtSPL8

tgccaagcagagggatgcaacgcggatctgagccacgcgaaacactaccacagaaggcacaaagtgtgcgaattccactcaaaagcatcgacggttgtagccgccggactaagccaaaggttttgccagcaatgcagcaggttccatttgctgtcggaattcgacaacgggaaacggagc
tgccgtaagcgacttgctgaccataaccgccgccgccgtaaatgtcac

>AtSPL9

tgccaagtggaaggttgtgggatggatctaaccaatgcaaaaggttattactcgagacaccgagtttgtggagtgcactctaaaacacctaaagtcactgtggctggtatcgaacagaggttttgtcaacagtgcagcaggtttcatcagcttccggaatttgacctagagaaaaggagt
tgccgcaggagactcgctggtcataatgagcgacgaaggaagccacag

>AtSPL10

tgccaaattgatggctgtgagctggatctctcatcttctaaggattatcatcgcaagcatagagtctgcgaaacgcattcaaagtgcccaaaagttgttgtgagtggcctggaacgtcgtttctgccaacagtgtagcaggttccatgctgtctcagaatttgatgaaaagaaacgaagc
tgccgcaaacgtctttctcatcataatgcaaggcgtcgcaagccacaa

>AtSPL11

tgccaaattgatggctgtgaactggatctctcatctgctaagggttatcatcgtaagcacaaagtctgcgaaaagcattcaaagtgcccaaaagttagcgtgagtggcctggaacgtcggttctgccaacagtgtagcaggttccatgctgtctctgaatttgatgagaagaaacgaagc
tgccgaaaacgtctttctcatcataatgcgaggcgtcgtaagccacaa

>AtSPL12

tgtcaggtagacaactgtggagctgatttaagcaaagttaaggattatcatagacgtcataaggtctgtgagattcattctaaagctactactgcacttgttggaggaattatgcagcggttttgtcagcaatgtagtaggtttcatgtgcttgaagagtttgatgagggaaagagaagt
tgccgtagacgtttggctgggcataataagcgtagaagaaaagcaaat

>AtSPL13

tgtcttgttgatggatgtgattctgattttagtaattgtagagagtatcataagagacataaagtttgtgatgttcattcaaaaactcctgtggttactattaatggtcataaacagaggttttgtcaacaatgcagcaggtttcatgctttggaggagtttgatgaagggaagagaagt
tgtaggaaacgtcttgatggacataatcgaagacgacggaagccgcag

>AtSPL14

tgtcaggttgataattgtactgaagatttgtctcatgctaaggattatcatagaaggcataaagtttgtgaagttcatagtaaagctactaaagctcttgttggtaaacagatgcagaggttttgccaacagtgtagcaggtttcatctgctttctgagtttgatgaggggaagagaagt
tgtaggcgtagattggctggccataatcgacggaggaggaaaactacg

>AtSPL15

tgccaagtggaaggttgtagaatggatctaagcaatgttaaagcttattactcgagacacaaagtttgttgcattcactctaaatcatctaaagtcattgtctctggtcttcatcaaaggttttgtcaacaatgtagcaggtttcaccagctttctgagtttgacttggagaaaagaagt
tgtcgcagaagactcgcttgtcataacgaacgacgaagaaaaccacaa

>AtSPL16

tgtcaggttgataattgtaaggaagatttatcaattgctaaggattatcatagaagacataaagtttgtgaggttcatagcaaagctactaaagctcttgttgggaaacagatgcagaggttttgccaacagtgtagcaggtttcatctgctttctgagtttgatgaggggaagagaagt
tgtaggcgtagattggatggtcataacaggcggaggagaaaaacacag

>GmSPL1a

tgtcaagttgatggttgtagcgctgatctaagtgaagctaagccctaccataggcgtcacaaggtttgtgagtaccatgccaaggctcctgccgtactcattggagaccagcaccaacggttttgccaacaatgtagtaggtttcatgagctatcagaattcgatgactcaaaaaggagt
tgcagaagacgtttggctggacataatgagaggcgtcgcaaaaatgca

>GmSPL1b

tgccaagtggacaattgtgatgctgatctgagtgaagctaagcagtaccacagaagacacaaggtttgtgagtaccatgccaaggctccttccgtacacatggcagggctgcaacaaaggttttgccaacaatgtagcagattccatgtgctatcagaatttgatgactcaaagaggagt
tgtagaacgcggttggctgggcataatgagaggcgtcgcaaatatgca

>GmSPL2

tgtcaggcagagatatgtggtgctgatttgactgttgcaaagaggtaccatcgccgtcataaggtgtgtgagcttcattccaaggctccttctgtgatggttgcaggactgaggcagaggttttgccagcaatgtagcaggttccatgagctggcagagtttgatgaagctaaaagaagc
tgccggagacgcttggccagacacaacgagcggcgccggaaaagcaac

>GmSPL6

tgtcaagtttatggttgtaacatggatcttagctcctcaaaagattaccacaaaaggcataaagtttgtgatgctcactccaaaacagctaaagttattgtaaatggaattgaacagaggttttgtcagcagtgcagcaggtttcatttgctagctgagttcgatgatggtaagcgcagt
tgtcgcaggcgtctagcaggacacaatgaacgccgaaggaaaccccaa

>GmSPL8

tgccaggccgagggttgcaatgctgatctgtcgcaagccaagcactatcaccgccgccacaaggtgtgcgagtttcactccaaagccgccaccgtcatcgccgccggcttgactcagcgattctgccagcaatgcagcaggttccatcttctttctgagtttgataacggaaaacgtagc
tgcaggaagagattggcagatcataatcgccgcagaagaaaaactcaa

>GmSPL9

tgtcaggttgagggctgcaaagtagatctgagtggtgctaaggcttactattctaggcacaaagtttgtaccatgcactccaagttccctactgttattgttgctggtttggagcaaaggttttgccaacagtgtagcaggtttcatctgctttctgaatttgatgaaggaaaacgaagc
tgccgcaggcgacttgctggtcataatgagcgccgacgaaagcccccg

>GmSPL9b

tgtcaagttgaagggtgcaaagtagatctgagtgatgcaaaagcttactattctagacacaaggtctgtggcatgcactctaaatccccttcagtcattgttgctggtcttcaacaaaggttttgtcaacagtgtagcaggtttcatcagcttcctgagtttgatcaaggaaaaagaagt
tgccgtaggcgactagctggccataatgaacgtcggagaaagcccca

>GmSPL12b

tgtcaggcagagatgtgtggtgctgatttgactgttgcaaagaggtaccatcgccgccataaggtgtgtgagcttcattccaaggctccttctgtgatggttgcaggactgaggcagaggttttgccagcaatgtagcaggttccaagagctggcagagtttgatgaagctaaaagaagc
tgccggagacgcttggccagacataacgagcggcgccggaaaagcaat

>GmSPL13

tgccttgtggatgggtgcaattcagatcttagcaattgtagagattatcataggcgccataaggtgtgtgagctccattccaagaccccagaggtcacaattggtggcttcaagcaaaggttctgccaacaatgtagcaggttccattcgctggagcaatttgatgaaagaaaaagaagc
tgcagaaaacgtttagatggacacaatagaaggagaagaaagccccag

>GmSPL14

tgccaggtggacaactgcagagaggatctgtcgaaggcgaaggactatcaccggagacacaaggtgtgtgaggctcatagcaaggcctccaaagcgcttctcgcgaatcaaatgcaaagattctgccagcagtgtagcaggtttcatcctctctcggagtttgatgaggggaagcggagc
tgccgccggagactcgccggacacaaccggcgccggcggaagacgcag

>GmSPL16

tgttctgttgatggatgcaattctgacctcagtgattgcagagattatcacaggcgccatagggtctgcgaaaagcactccaaaaccccggttgtgttggtgggggggaaacaacagaggttctgccaacaatgcagcaggtttcattcacttggggagtttgatgaggttaagaggagt
tgtaggaaacggcttgacgggcataacaggcgccggaggaaacctcag

>GmSPL17

tgtcaggttgcgggctgcaaagtagatctgagtggtgctaaggcttactattctaggcacaaagtttgtgccatgcactccaagtcccctactgtcactgttgctggtttggagcaaaggttttgccaacagtgtagcaggtttcatctgctttctgaatttgatcaaggaaaacgtagc
tgccgcaagcgacttgctggccataatgagcgccgacgaaagcccca

>MsSPL2

tgccaagcagagaggtgtggagctgatttgacggatgcaaagagataccatcgccgccataaagtgtgtgagtttcattccaaggcacctgttgtggtggttgcagggatgaggcagaggttttgtcaacaatgtagcaggttccatgacttggtagagtttgatgagtctaaaagaagc
tgccgcaggcggttggctggacacaatgaacggcgccggaaaaccaac

>MsSPL3

TGTCAAGTTGATAACTGTAATGCTGATCTAAGTGTTGCTAAGCAGTACCATAAGCGCCATAAGGTTTGTGAATACCATTCTAAGGCTCATTCCGTACTCATTTCAGAGCTTCAACAAAGGTTTTGCCAGCAATGTAGCAGGTTTCATGAGGTATCAGAATTTGATGACTTGAAAAGGAGT
TGTAGGAGGCGTTTGGCTGGACATAACGAGAGGCGCCGCAAAAGCACG

>MsSPL4

TGTCAAGTGGAGAATTGTGATGCTGATCTTAGTGAAGCTAAGCAATATCACCGGAGACATAAGGTCTGTGAGTATCATGCCAAAGCACCTACCGTACACATTGCAGGATTGCAGCAAAGGTTTTGTCAGCAATGTAGCAGATTCCATGGACTATCTGAATTTGATGACTCAAAAAGAAGT
TGCAGAAGGCGTTTGGCTGGGCATAATGAGAGGCGCCGCAAAAGTGCA

>MsSPL6

TGCCAAGTTTATGGTTGTAACATGGATCTTAGCTCCTCGAAAGATTACCACAAAAGGCATAAAGTTTGTGACGTTCACTCCAAGACAGCTAAAGTTATCGTCAATGGTGTTGAACAGAGGTTTTGTCAGCAGTGCAGCAGGTTCCATCTGGTAACTGAGTTCGACGATGGTAAGCGCAGT
TGTCGCAGGCGTCTAGCTGGACACAATGAGCGCCGAAGGAAACCTCAG

>MsSPL9

TGTCAAGTTGAAGGATGTAAACTAGATCTGACTGATGCTAAAGCTTACTATTCTAGACACAAAGTTTGTAGCATGCACTCTAAGTGCCCAACTGTTACTGTTTCTGGTCTACAACAAAGGTTTTGTCAACAATGTAGCAGATTTCATCAGCTTGCTGAGTTTGATCAAGGAAAAAGAAGT
TGCCGGAGACGACTAGCTGGTCATAACGAGCGTCGCAGAAAGCCCCCA

>MsSPL12

TGCCAGGTGGAAGGTTGTGGCCTCGACCTCTCTTTGGCTAAAGATTACCATCGGAAACATAGAATTTGTGACAGTCATTCCAAATCGCCTGTGGTGGTAGTAGCTGGTTTGGAGCGTCGATTTTGCCAGCAGTGTAGCAGGTTCCATGATCTCTCAGAGTTTGATGATAAAAAAAGAAGC
TGCAGACGCCGTCTTTCAGATCACAATGCAAGGCGTCGCAAACCTCAG

>MsSPL13

TGTCTTGTGGATGGGTGCAATTCTGATCTTAGTAATTGTAGAGATTATCATAGGCGTCATAAGGTTTGTGAACTTCATTCTAAGACTCCAGAGGTTACAATTTGTGGCCTTAAACAAAGGTTCTGCCAACAGTGTAGCAGGTTTCATTCGCTGGAGCAATTTGATGAAAGAAAAAGAAGC
TGTAGGAAACGTTTAGATGGACACAACCGAAGAAGAAGAAAACCACAG

>MsSPL1 (Medtr2g046550.1)

atggatgctgattttgaggggaaaaatcagcatttgtatgaagcagtagtgcctgagatgaagggtgctgttggtagggggagtaaggattgggatttgaatgattggagatgggatggtgatctttttactgctaagcaactgaattcggtgccgacggattg
taggaatcgtcaatttttacccgaaatacgtgaaaatgttgatgtttcgaataatttgatttctggggaaggtagtagagagttggagaagaggaggaggggttttggtggtgaagggttggagatgaacgatgattttggttctcttaatttgaatcttgggg
gtcaagtttaccctataatggatggggaggaaaagagtggtaaaaagacaaagattgcgcctgtgcctacttcaaatcgtgctgtttgtcaggtggaggattgtagggcggatcttagtaacgcaaaggactatcatcgtcgacacaaagtttgtgatgtgcat
tctaaggctagtaaggcacttgtgggaagtgttatgcaacgattctgtcaacagtgtagcaggtttcatgttcttcaagagtttgatgaagggaagagaagttgtcgtaggcgtcttgcaggccataataagaggaggaggaagacacatcctgatactgctgt
agttaatggaggttctccgaatgaagaaaggggcagtagctatctgttgatgagtctactaaggatattgtccaatatgcattcgaacggcccagatcatacgagaaacttggatggtctatcccatctaattgggaacctgacaagtttagctggtacattta
acggaagaaatatagcatccttattggagggacctcaagaattagtaaaagccggtacatctggagctgcacagaatgttccaaactcaaatccgaatggcgctgaaccatcaagaccagattcttctattgaaatgacaaacggtctgatccatcaggaccct
ccagagtctaggttacagtgcgcgacagttccggccaatcatttgacacaaaaatgtataccttcaagcagtgttggagtaggatgtttaaaacctcctctgataccgcagtcctcaaatttagttccttcaaggggtagccttccacctcgaccagttgccac
agagactacagttggaagaaacaggttatgtaatattgacctgaataatgtatatgatgatggacaggactatgttgagaaccctgaaaattctaatccacctttggccttaggggttgaatcccgtgatcattcttcatttgtacaatatgaatctctcaagt
caagtccaccacagacgagtagaaactcagattcgacttctactcaatcaccatccagttctagtggagaaggccagagtcgaacagatcgaattgttttcaaactctttggcaaggatcctaatgatatcccccatgttcttcgatcacaggtccttagctgg
ttatccaacagtcccaccgagatagagagctatataagaccaggctgtattattttaactatatgtctccgcctagaaaattctgcatgggatgagctatgctataatctggggcccagcttgagaaagcttcttgctgcatcaaatgattctctttggagaac
aggatggatatataccagagtgcagcactctgtagctatttgtataatggtcagctggtcttagatgtgccatcgcgtcttggaagcccacagaattgtcagattttgtgcattaaaccccttgctgtctctgcaaatgaagacgtaaaatttactgtgaaag
ggttaagccttttcttgtctagtgcaaggttgctctgtgcacttgaagggaagtacctggtagaggacatgtgttatgatttgattgatggagctgatgcagccattggacatcatgagcttcaaactctcagcttctcttgtcatataccaaatatgactgga
agagggtttattgaggttgaagataatagtctcagcagctgttcctttccattcatagttgcagagcaagaaatttgttccgagatatgcagtctggaaactatcattgaggcggcagagactgctgatgacatccagatcaaagccaaattaatggaagaaaa
gactcgagcaatgaatttcgtacaagaaatgggttggctccttcatagaattcgtataaaattccggctagggccaatgacacctgttcaagatcgtttccatttaaatcgatatacatggcttgtcggcttctctatggaccatgattggtgcgctgtgatga
aaaaactcttggacactatctttgaaggtgaagttgatactggagaacatatctctgcagagctagctttgttaaacatgggtcttctacacaaagctgtaaagagaaattgtaggcctatggtggaactattgttaaactttgtgccagttaaagcatcagat
ggtggagatagtaaagagatgcaagtcaataaggtccccgacggattcttgtttagacctgatactgttgggcctgctgggttgactccccttcatgtcgcagcaagtatgaatggctatgagactgtattggatgcgttaactgatgatccgggaatggttgg
aattgaggcatggaaaagtgccaaggacaatacaggtttgacacccaatgactatgcatccctaagaggccactactcctacattcaacttgtgcagaggaaaacaagcaagagcagccaaactcaacatgtgcttgacatcccaggcacccttgtagacggaa
acacgatgaagcaatcagatggacataagtcatcaaaggtattgagtttgcatacagagaagattgcaacaacagcaattccaaatcattgtgggatctgccagcagaagttggcatatggcagtgtcggcggcatgaggagggcacttgtttataggccggcg
atgctgtcaatggtcgccattgcagctgtatgtgtctgtgtagctttgctcttcaaaagttcaccaagagtttcctatgtattccagcctttcagctgggaatcattggattatggttcaatctaa

>MsSPL1a (Medtr7g110320.1)

atggaagcttttcaattgtacggtaatggtggtggttcatctgatttaagggctatggggaaaaattcaaaagagtgggatttgaataattggaaatgggatagtcatcttttcatagctactagtaagttgactccggtgccggaacaccgccaatttttgcc
tattccggttggtggtggtggtggtggtggttctaactcaaatagttcatcatcttgttcagaacagttagatcttgggatttgtcaagtgaaggaaggtgagaggaaaaggagggttattgttgtcgaagatgaactaggtttaggtttgaataaggaag
gtggtaatcttagtttaaaccttggtggtggtgttgctacatgggaaggaaataatgggaagaaaagtagagtagctggtggaggtagttctagtagagcattttgtcaggtggaggattgtcgcgcagatcttaacaacgcgaaagattatcaccgacgtcat
aaagtttgtgagatacattcaaaggctagtaaagcccttgtcggaaatgcgatgcagcggttctgccaacaatgtagtaggtttcacttgcttcaagagtttgatgaaggaaagagaagctgtcgaagacgtttggcaggccataataaaggaggagaaaaac
aaatcaagatgctgttcctaatggaagttcaccgaacgatgatcagactagtagttatctgttgataagtttattgaagatactttcaaatatgcagcctgacaggtcaaatcagactgccgaccaggatctgcttactcatcttttaaggagtcttgctaatc
agaatggcgaacaaggggggaggaacttgtctaacctttacgggaacctgagaatctgttaaaagaagggtctttatcggggaagtctgagatggtttctactttagttaccaatggttctcaaggttctccaactgttacagtacagaatcaaacagtttct
attagtgaaattcagcatcaagtgatgcattcacatgatgctagggttgctgatcaacaaaccacattttctgcaaagcccggtgtttcaaatagccctccagcttactcagaagcaagagatagtactgctggacagaccaagatgaatgattttgatctgaa
tgacatctatattgactcagacgatggcatagaggacatagaaaggttgcctgtcactacaaatcttggtgctagctctcttgattacccgtggatgcaacaggattcacatcagtcgagtccacctcaaacaagtggaaattcagattctgcatctgcccagt
ccccttctagttccactggcgaaactcagaatcgcacggatcggattgtttttaaactcttcggtaaaggaccgggcgatttcctcttgtacttaaagcacagattcttgattggttatcccacagtccaactgatattgaaggatatatacggcctggttgt
gttgttttgactatttatctgcgtcaggctgaggttgtgtgggaggagctttgctttgatctgacttccagtttgaataggctcctgggtgtctcagatgatgatttttggagaactggatgggttcacatcagggtgcagcatcagatggccttcattttcaa
tggccagattgtcatcgatacacccctcctttcaggagtaataattatggtaagattttgagtgttagtccaattgctataccatcatcaaagacagctcaattttctgtgaagggtatcaacctgactcgccctgccacaaggttattgtgtgctttagaag
ggaattatttggactgtgaagatactgatgagcctatggatcaatgctccaaggatcttgatgagcttcagtgcatccaattttcatgttctgtccctgcgatgaatggacgagggtttattgagattgaggaccagggtttgagcagcagtttttttccttc
atagttgtggaggaggatgtttgctctgaaatctgtgtgcttgagcctttactggagtcaagtgatacttatccagataatgaaggggctggaaaaattcaagcaaaaaatcaagccatggatttcattcatgaaatgggttggcttctccacagaaggcagat

[illegible]
aaaatctagcgttcgtttgaattctagtatggatctgtttccattagatcgattcaagtggctcatggagttctctgtggaccatgattggtgtgcagtagttaaaaagctattgaacctcatgcttgatggaacagtgagtacaggagaccacacatccctgt
atcttgcactttcagagttgggtctccttcacagagctgtgaggagaaatagcaggcagttggtggagcttcttttgagatttgttcctcaaaatatttcagataaactaggacctgaagacaaggcgctggttaatggagagaatcaaaacttcttgtttaga
cctgatgctgtgggtcctgctggtttgacaccgcttcatatagcagccgggaaagatggttctgaggatgtactagatgctttgactaatgatccttgcatggtgggaattgaagcatggaatagtgctcgcgacagcacagggtcaacacctgaggattatgc
acgattacgtggtcattatacttacattcatttggtgcaaaagaaaatcaacaagagccaaggaggggctcacgtggtggttgatattcctagcattccgacaaagttcgacacaagccagaagaaagatgaatcgtgcactacctttcagattggaaatgctg
aagttaaaaaggttcgaaaggactgcaaactttgtgatcataagctgtcgtgtagaactgctgtacgaaagtctttcgtatacagacctgcaatgctgtcaatggtggctatagcagcggtctgtgtctgtgtggctcttctattcaaaagctcgccggaagtt
ctgtatatattccgacctttcaggtgggaatctttggattatggaacaagctga

>MsSPL2a (Medtr8g080680.1)

Atggacttgaatgcaatatctccctcttcacagtgggaatgggatcacctaccattactcgatacaaaagcaactgaaaaccaaaaattacaaccaccaaactggactatggaacttgacccagaaatcaatttcgggttgtatgatacgcccgttggaagtgg
ttcttctggctctgacctaattcacggttcagcatttaatgcaccaaagttttcttgtgtatctggtgaaccattgctcaatccaagacttggtaaaaggatgtattttgaggaagtttgtccagaaagtgattccaaaaacctttctttttcaagggatctca
tgtcatctttaattcttgaaaagaaatgcaagtccaatggtcagaacttacaatgtccaccacactgccaggttgaaggttgtggccttaacctttcatcggcaaaagattatcattgcaaacgaagagtttgtgaaagtcatgccaaatctcctatggtggtt
atagatggtttggaacgtcgattttgccagcaatgtagcaggttccatgatctgtttgagtttgatggaaagaagaaaagctgcagacgacaactttcaaatcacaatgcaaggcgccgtaaatatcatcgtcaagctgtccaatcaagccaatcggctttgtc
ttattcacgcagtgatgggaagcaacagatgagtccatttactaattccaagactgctacaaatttagcctggcaaaacatgcacaacagcaagctcccccaagcaaaagattttctcttgaagccttcaaaagacaacattgacacaccaagtactgtcacta
tgctttctcatgactccaatttccattttacatccaaaatccttgcaaccaagagtgttaatccaggtgtagaagatttcatgaacttttctgatacaaatgctacacaagactttacttgtgctctctctcttctgtcaaccaatcagtgggattcatatgcg
actaaatccatttctcaagaacattctaatcgcccgaccagtccttttcaagccacaactcatgcaatgagtcagtgcattcctcttgcttcatcagaaccgtgctgtggtcatcatgatcaatatgtcaattccaacatctggatctcaaattctgcagatag
caatcactttcagaaatttcaactgttcagagaaccttatgagttatga

>MsSPL7(Medtr2g020620.1)

atggaatccaaatctcaaccaattctccctccaatggatcctcccgaagatctctcctccgtttgggacctctcttacctcctcgacttcgatgacgatattccacaactcccgcctcttcctaaccctaatcctactccagaagaaaacgagaggatccgaaa
gcgtgacccgaggttaacttgctccaactttctcgcgggtcaagtaccatgtgcatgccctgaactcgatgctttgttagaggataacggtttaccaggaaagaaacgagctcgaactgcacgcttgatattctctgagcttaaaggctatcatcgacggcata
gggtttgtttgcggtgtgctaatgctgctactgttgttcttgatggtgatgtgaagagatactgccaacagtgtgggaagtttcatgttttatcggatttcgatgaagggaaacggagttgtagaaggaaattggagcggcataatactcggaggaggagaaag
gccgtagattctgcagtaggagttgataatgaagttcaaactgttactcaaaatgatgattccaattgtgatggagaactaggaatagattattcgaatttgagtagggagaatatcgaaaaaagagcgttgcaggatcatgaagaagaaccggttgttaatgg
ttcgtcaacaccagagacacagaatattaatggtgacagtgtcgtttcctttgttgcttctgctgaaacacaagcgaatattggaaaagatgtttccgatccttctaagtcgccttcatattgtgacaataagagtgattattcgtctatgtgccaaacaggtc
gggtttcttttaagctttacgactggaatcctgcggaatttccgagaaggctgcggctccaaatatttcaatggttggcaagtatgcctgtagagctggagggatatatccgccctgggtgtactatcctgacaatttttattgcaatgccaaatattatgtgg
ataaatttactcaaagaccctatgtattatgtgcgtgatcttgctgctcccagaaatatgctatctggaagaggaaccgctctaattcatctgaatgatatgatcttccgtgtaatgaaagatggaatttctgtgacaaaagtcgaggtaaacatgcaggcacc
taggcttcactatattcatcccacatgctttgaagcaggaaaacccatggaatttttttgcctgtggaagtaacttgctgcagcctaaatttcggcttcttgtatcattttatggaaagtatctgaagtgtgagtattgcgctccgtctccacataactcggctg
aagacaatatttcttgtgcttttgataatcagttatataagatatgtgtccctcatattgaggaaaatctcctggggcctgcatttattgaggttgagaatgagtctggtttatcaaactttattcctgtacttattggggataaagaaatttgcactgaattg
aagatattgcaacaaaaactagacgcttctctccctttctaaacaatttcggtctgcatctggtagttctatttgtagctcatgtgaagcttttgtacatattcacacatcatcatcagacttgcttgtagatatagcatggttacttaaagaccctacttcaga
aaattttgacagaatggtatctgcttcacagattcaaagatattgctatttgttggattttctaatatgcaatgattcaactatcatcttgggaaaaatattgccaaatttgataagcataacgaaaagcatgaaatccaatataagtgatgttgacatggacc
aactgctgaagggcatgtgtaatgctagagatgctatttgtcggaaaggtggaggtatagttttgaactccaaaatggaaggcttcaaacctgctcagtgcagttcccaaaatgccaagctatcagttgttgaagttaacagtcagggtatccagtttagagca
gatgcagagttgggagttctgagcagtctcgcttctgatgaaaaaaatcagaagataccgcttttgaaaagggatattataatgaatatggaagagttgcctaaaagatgtgaccatcaaaatcttacccggggattcttgagatctcgaccaactacttttgt
gcttgtatcacttgttgtttgtcttgcagtatgtgtatccgtctttcatcatgggagggttaacgagttggcagtatccattagaaggtgtttgttcaaccattga

>MsSPL7a (Medtr7g444860.1)

Atggactttggagggaacatgttctgtctaaacaacagagaccaaagtaatactaacaataacggtaacagtaacggttttacttggtgtagtagtggtagtactactactggtactgcatggaacatgaatactttcaatgttaatgaggtaaacaattcagt
ctccgccgccaaccgtactgaggcaggtctagctaacgcgcttatgtacctgcctcaaaatgaaggcggcggacgacagtatcaaagtgcatacggtggaggtgtgtctcggggtcatatgatgcctgatccacacctcacgtgtttgaagttggggaagaggc
attattttgaggatgtgagtggtggtggtggggtgatgggggagaagaaagggaaaggagggtattgtggtggtgggaagacggcggcggtgggaggttatacggcggtgacaaggtgtcaggtggatgggtgtaacgtggcattgatgaatgctaaggagtat
cataggagacataaggtttgtgagatgcattcaaaggcaccaaaagttgtggtgttgggtttggagcaaagattctgtcaacagtgtagcaggttccatgttgtatcagaatttgatgattcaaaaagaagttgtagaaggagactggcagggcacaatgagag
aagaagaaagagctcccatgattctgttgggaggaattcctcacaaggtgggtgtgctctctctcttctgtcatctcggactgattcatggctttcaccggctgacctctccgtaagatgcagtgctgcattaagcgaattgatagcagaaaaccgagcatcga
taatggctaggcaatatgtctcagacagagactggcatttgcagcaccatgccgtggaagactacaaagagatccaatccgagtccaattatttcccacaacatatgtttccgcagacacattag

>MsSPL8 (Medtr8g005960.1)

Atgttggactatgaatggggtaacccatcaaacatcatgcttaccggaaatgaagacaactccggtgcagcagccaccgatcaagcccaccgtcaaatcttcgaccactatgcttctcaagctatgttatccgacaactaccttaacggacctggtggtggacc
caccatcgacataaacagtggtgatttactcatcaccacaaccagtttagcccacacaaccagcagcaccataatatccatcagtccttcttcgacccacgggcttttcatggagggtcatcaactgcttcttcttaccctccacctcaaccacaacctcctc
cttccatgctctcccttgaccctcttcctggtcatggacatagccctggattcctcctcgtaccaaaatccgaggatgttaacagacccattgacttcgtgggctctagacttggactcaaccttggtggccgcacttacttctcttccgaagatgacttcgtg
acccgcctttaccgtcggtctagaccacctgaaccgggttccacaggttcttcgaactcacctaggtgtcaagccgaaggttgcaatgctgatctatctcaggctaagcattaccaccgccgccacaaagtttgtgagtttcactcgaaggcagccaccgtcgt
tgcagctgggttgactcagcggttctgccagcaatgcagcaggttccatcttctatctgagtttgataatggaaaacgtagctgcaggaagagattggctgatcataatcgtcgtaggagaaaaactcagcaccctaatactcaagatattcacaaatctcaca
atactttggatagttctgccacaagatcccctccggagtctggaactcaatccacatcttctgtgactgtggccgtatctccaccggattattttcgacaaaggtcgtaccaaaccccaagcccttcaacaacttcaagctcaatgtttttctccaccgggtag

>MsSPL13a (Medtr8g096780.1)

Atggagtggagtaatttgaaagcaccttcatgggatttaactgaaatggatcaaggaaccttaaacaacatagaaacaatggatggttcaattaggaggtttggagattacagaacaaaaggagattttctgttgatttgaagcttggccatgttggaaattc
tagcactgaatcagggctaacaaagtccaaagatgttggtggatttaccaaaatgacatcttcatcatcatcaacttcagggtcatctaagagagctagagctattaacaatgggactcagatagtttcatgtcttgtggatggttgtcaagctgatcttagta
actgtagagactatcataggcgtcataaggtctgtgaacttcattccaagactgcacaggtcacaattggtggacataagcaaaggttctgccagcaatgtagcaggttccatgctctagaggaatttgatgaaggaaagaggagctgtaggaaacgcttagat
ggtcacaaccgacggagaagaaaaccacaaccagaacctattactcgaagtagtagctttttgtccaattaccaaggcacccaattgctgccattctcaagttcacatgtatacccttctactgctatgatgaatcctacttgggttggaggagatgttaggct
ccatagccacaatcaacaccaacaaatgcaccatctggtcgataaacaagacctttttcttggaacaacatcttcaaccagcagctacaaagaagggaaacaagtctcattcatacaaaataaccagccaacagcagcagccactatgaacaatcaacctcttc
cagcttcatccatgagtcgcacgtttagcaggaccaatccttttcagaaagatacaaaatgttttgtgacaacaacacttcttgtgctctctctcttctgtcatcaccagtaccacaaacacatgatcatcctgaaaatggattgaatcagatggtgaatact
cactcatcttttatgcaacccttaggcttgagtttgcatgaccatagcttggggtctgtggactcagttttgggttcaaatgatcagagtggtcattgttcctcaatgtataacatgggttctagtgaatcccattctcaggggaataataatgaagcttctcc
attatttccttttcaatgggattag

>MsSPL14 (Medtr1g035010.1)

Atggaaaaagtagctcctcctcttcttcctcttcatcctcctatgttatcatcacatcaattttacgattcatcaaacaccaaaaaacgtgatctattatcatcatacgacgtcgttcacatcccaaacgataattggaaccctaaagaatggaattgggacag
tatccgtttcatgacagcaaaatcaacaacagtagaaccacaacaggtcgaagaatctctaaacttaaatctcggaagcacgggactcgtacgaccaaacaaacgaatccgttcaggatctccaacatcggcttcatatccgatgtgtcaagttgataactgca
aagaagatctatcaaaagcgaaggattatcatcgtcgtcataaggtgtgcgaagctcatagtaaagcttctaaggctttgttagggaatcaaatgcaacggttttgtcaacagtgtagtaggtttcatccgttggtggagtttgatgaagggaaacggagttgt
agacggagacttgctggtcataatcggcgtaggaggaaaacgcagccggatgaagtggcggttggaggttcgccgccgttgaatcaggttgctgctaatttggagattttttaacttgttgactgctattgctgatggttctcaaggaaaatttgaggaaagaag
gtcacaagttccagataaagagcagcttgtacaaattctgaataggatccctttacctgctgatcttactgcaaagttgctggatgttggtaataatttgaatgcgaaaaatgataatgtccaaatggaaacttcaccctcctatcatcatcgcgatgatcaac
tgaacaatgcccccctgccccgttaactaaggatttcttgctgttctttccacgactccatccacacctgctcgaaatggcggcaacgggtccacaagtagtgccgatcatatgagagaaagaagcagcggcagctctcagtctccaaatgatgattcagat
tgccaggaggatgttcgagttaaattgccgctgcaacttttggctcctctccagaaaacgacagcccgtctaaattgccatcttcgcgaaagtatttttcatcggaaagtagtaatcctgtggacgaaaggacgccgtcgtcatctcctcctgttgtggagat
gaattttggtttgcaaggtgggattagaggtttcaattctaattgtatttccactggatttggaggcaatgcaaataaggaaactagccaaagtcatagttgtactactatccctcttgatctctttaaaggttcaaaatctaataacatgattcaacaatcta
gttcagttcaaagtgttccatttaaagctggttatgcatcttcaggctctgattattcacctcccagtttgaattcagatactcaggatcgtactgggagaataatgtttaaattgtttgacaagcatcctagccatttcccaggaacgctgcgaacacagatt
tacaattggctttctacacggccatcagacttggagagttacataaggcccggctgtgttgtcctatcaatttatgcttcaatgtcttctgctgcttgggtgcaattagaagaaaacttcctccaacgtgttgattctttaattcataactcagattctgattt
ctggagaaatggaagatttcttgtttattctggcagtcagttagcatcacacaaagatggaaggatacgcatgtgcaaaccatggggaacatggaggtctccagagttgatatcagtgtctcctttggcaattgttggtggacaggagacttctatttcattga
agggaagaaacctgtctgctcctggcacaaagattcattgcactggtgctgactgttatacatcgtcagaagttataggatcaggagatcctggtatggtctatgatgagataaaattgagtggttttgaagttcagaatacatctcctagtgttctaggtcgc
tgtttcattgaggttgaaaatggtttcaagggaaacagttttccagtgataatagccaatgcgtccatttgcaaggagttgagaccgcttgagtctgaatttgacgaggaggaaaaaatgtgtgatgccatttcagaggagcatgaacatcattttgggaggcc
caagtcaagggatgaggctttgcacttttttgaatgagcttggttggctgtttcaaagggaaaggttctcgaatgtgcacgaggttccagattattcgctcgacaggttcaaatttgtactgacgttttctgtggaaagaaactgttgcatgttggtcaaaaccc
ttctggatatgctggttgataaacacttcgaaggagaagggttgtcaacagggtcagtggagatgctgaaagcaatccaacttctaaatagagcagtgaaaagaaagtgcacaagcatggttgatttgctcattaactattctataactagcaaaaatgacaca
tccaagaaatatgtatttccaccgaatcttgagggccctggtggcattactcccttgcatttggcagcatctacaacggattctgagggtgtaattgactctttaacaaatgatccacaggagattgggttgaagtgctgggaaacccttgcggatgaaaacgg
gcagactccacatgcctatgccatgatgagaaataatcactcttataatatgttggttgctcgtaaatgttccgatagacaaagaagtgaagtttcagttagaattgataatgagatagagcacccatcactgggaattgagctaatgcaaaagcgaattaacc
aagtcaaacgagttggggactcctgttccaagtgtgcaattgcagaagttcgcgccaaaagaagattttccggttcacggagctggcttcacggtccctttattcattcgatgctagctgttgctgctgtttgtgtatgtgtttgtgtgttattccgtggaacc
ccttatgtaggctcagtttctcccttcaggtgggaaaacttgaattatggcacaatgtaa

>MsSPL16 (Medtr7g028740.1)

Atggattgggattggaaagagtttgcttgggatccatctggatttgatgaggagttgaagaatgaaggagattcaatggatttgaggcttggtgatcaagcttctgattcattggaaaaatcagttcatgattcaggaaaggaagattcaaaagctgtgtcatc
gtcgttgtcgcctagtggatcgttgaaaagatcgcgtttacaaaatggatcgcagagtatgatttgctcggttgatggatgtaactctgatcttagtgattgtagagagtatcataagcgtcatagggtgtgcgaaaagcattccaaaactcctgttgtgttgg
ttggaggtaagcagcagaggttctgccaacaatgcagcaggtttcattcacttgcggagtttgatgatgttaagaggagttgcaggaaacgtcttgatggacataataagcgcagaaggaaacctcagccaccttctcttttcatggctgctgagaagttcatg
tacaattacaaaggacctagaatcctgcactttggtagccctcagacatatgtgaatcctattatgagaaacatttggcctgcgacttccataactgaagctgaatctggttacgatcatcaaagacttttgtatcgaattgacaaacataaacaggataaggg
acatcctcattggcaagaaattctccctaaatctggcgatgttaacaaagcagcgcctggaactcctatcagccatcctatccgcggtgccgttggttcatccgcaggtgaaaaaggcggtaggaagctctcttctgatggcaagcccggatcttttgactcgg
gttgtgctctctatcttctgtcaacacttcaaacacaaagctcagagttgagtttgatgcagtcaagcatcaacagtccaacacaatcttcgtctggaacgttgcattttgatgctacaaaggagtactcttactccggaaaggtgaaggataagccgaatggt
ccggtatttgttctagatggaaacacaacaaaccttcattgcaatgggatgctgcaaatgggtcctaatggattgatcgaaaacgataactcaatgactcttccattcttctgggagtaa

Table S10. DEG associated with glycolysis and TCA fold change in SPL13RNAi plants

Control	Flooded	Gene name	Gene ID	Gene annotation
0.292283	4.07574	ACS	Medtr7g079080	1-aminocyclopropane-1-carboxylate synthase
1.23672	2.54512	ACS	Medtr8g028600	1-aminocyclopropane-1-carboxylate synthase
33.2025	22.6475	ACS	Medtr8g098930	1-aminocyclopropane-1-carboxylate synthase
0.407777	0.046786	ACS	Medtr4g097540	1-aminocyclopropane-1-carboxylate synthase
52.2622	336.989	ACO	Medtr5g085330	1-aminocyclopropane-1-carboxylate oxidase
46.8868	165.751	ACO	Medtr3g083370	1-aminocyclopropane-1-carboxylate oxidase
14.4589	42.9741	ACO	Medtr2g025120	1-aminocyclopropane-1-carboxylate oxidase
7.47745	19.7655	ACO	Medtr3g088565	1-aminocyclopropane-1-carboxylate oxidase
1.17664	0.189009	ACO	Medtr1g043760	1-aminocyclopropane-1-carboxylate oxidase
5.80576	0.381962	ACO	Medtr8g024120	1-aminocyclopropane-1-carboxylate oxidase
6.46515	0.357816	ACO	Medtr1g070120	aminocyclopropanecarboxylate oxidase
0.05795	7.51682	ACO-L	Medtr8g028435	1-aminocyclopropane-1-carboxylate oxidase-like protein
1.59279	48.2452	ACO-L	Medtr1g032220	1-aminocyclopropane-1-carboxylate oxidase-like protein
1.16709	23.3807	ACO-L	Medtr1g032250	1-aminocyclopropane-1-carboxylate oxidase-like protein
0.057876	0.44284	ACO-L	Medtr1g032140	1-aminocyclopropane-1-carboxylate oxidase-like protein
4.21246	8.38377	ACO-L	Medtr4g099390	1-aminocyclopropane-1-carboxylate oxidase-like protein
46.4123	15.3528	ACO-L	Medtr4g117880	1-aminocyclopropane-1-carboxylate oxidase-like protein
60.8768	43.9499	GPI	Medtr5g065880	glucose-6-phosphate isomerase
46.6782	29.2285	GPI	Medtr6g009330	glucose-6-phosphate isomerase
21.9428	78.6897	GPI-L	Medtr7g116910	glucose-6-phosphate 1-epimerase-like protein
4.77392	0.722638	GPI-L	Medtr4g092780	glucose-6-phosphate 1-epimerase-like protein
62.6073	41.0816	G6PDH	Medtr1034s0010	glucose-6-phosphate 1-dehydrogenase
24.1502	14.5054	G6PDH	Medtr0590s0010	glucose-6-phosphate 1-dehydrogenase
21.5236	11.1966	G6PDH	Medtr7g111760	glucose-6-phosphate 1-dehydrogenase
20.9832	11.2963	G6PDH	Medtr0275s0010	glucose-6-phosphate 1-dehydrogenase
7.75127	378.812	GlgC	Medtr4g070430	glucose-1-phosphate adenylyltransferase family protein
15.2253	99.1674	GlgC	Medtr3g116860	glucose-1-phosphate adenylyltransferase family protein
118.531	208.528	GlgC	Medtr5g097010	glucose-1-phosphate adenylyltransferase family protein
64.9239	48.9034	GlgC	Medtr3g082150	glucose-1-phosphate adenylyltransferase family protein
145.958	34.3167	GlgC	Medtr4g131760	glucose-1-phosphate adenylyltransferase family protein
0.806535	0.182397	GlgC	Medtr7g111020	glucose-1-phosphate adenylyltransferase family protein
9.28271	560.857	APE2	Medtr2g022700	glucose-6-phosphate/phosphate translocator-like protein
433.367	174.379	FBPase	Medtr7g066120	fructose-1,6-bisphosphatase
25.979	13.2709	FBPase	Medtr4g093620	fructose-1,6-bisphosphatase
250.3	72.776	FBPase	Medtr6g035305	fructose-1,6-bisphosphatase, cytosolic-like protein
0.501292	0.087603	FBPase	Medtr2g008030	fructose-1,6-bisphosphatase, cytosolic-like protein
28.2472	47.7547	FBA	Medtr1g061050	fructose-bisphosphate aldolase
193.732	129.19	FBA	Medtr5g069050	fructose-bisphosphate aldolase
108.064	8.29535	FBA	Medtr5g096670	fructose-bisphosphate aldolase
255.707	147.846	cFBA	Medtr4g057670	fructose-bisphosphate aldolase class I
2805.73	1255.73	cFBA	Medtr4g071860	fructose-bisphosphate aldolase class I
39.8398	55.0008	pFBA	Medtr6g069660	fructose-bisphosphate aldolase class-II protein
49.3714	22.8812	FKFBP	Medtr3g464770	fructose-6-phosphate-2-kinase/fructose-2, 6-bisphosphatase
4.64322	2.48202	FKFBP	Medtr8g066450	fructose-6-phosphate-2-kinase/fructose-2, 6-bisphosphatase
43.0044	18.6331	FKFBP	Medtr5g071900	fructose-6-phosphate-2-kinase/fructose-2, 6-bisphosphatase
32.9084	64.1851	PFP	Medtr4g132700	pyrophosphate-fructose-6-phosphate 1-phosphotransferase
86.4387	43.6765	PFP	Medtr1g035230	pyrophosphate-fructose-6-phosphate 1-phosphotransferase
75.3817	32.4042	PFP	Medtr2g025020	pyrophosphate-fructose-6-phosphate 1-phosphotransferase
11.5014	7.90297	GFAT	Medtr7g080270	glucosamine-fructose-6-phosphate aminotransferase
1.50605	19.7827	LKR/SDH	Medtr4g124470	lysine-ketoglutarate reductase/saccharopine dehydrogenase
21.416	60.8072	DUF707-L	Medtr2g012570	lysine ketoglutarate reductase trans-splicing-like protein
0.188594	0.683537	DUF707-L	Medtr4g058800	lysine ketoglutarate reductase trans-splicing-like protein
4.72316	9.38377	DUF707-L	Medtr2g005330	lysine ketoglutarate reductase trans-splicing-like protein
3.76927	8.40902	DUF707	Medtr8g089820	lysine ketoglutarate reductase trans-splicing protein
22.9177	40.6687	DUF707	Medtr3g086610	lysine ketoglutarate reductase trans-splicing protein
0.036409	0.391954	GLR	Medtr2g015310	glutamate receptor 27
1.61681	2.70688	GLR	Medtr3g105595	glutamate receptor 32
16.0473	25.5125	GLR	Medtr2g088450	glutamate receptor 32
28.4751	43.982	GLR	Medtr5g024350	glutamate receptor 32
1.72447	0.8265	FPGS	Medtr1g028510	tetrahydrofolylpolyglutamate synthase
30.2332	21.5555	GLT	Medtr1g027020	NADH glutamate synthase
375.259	225.587	GLSF	Medtr7g089970	ferredoxin-dependent glutamate synthase
10.0575	33.0235	GAD	Medtr3g064740	glutamate decarboxylase
10.6646	1.87683	GDH	Medtr6g029460	NADP-specific glutamate dehydrogenase
40.4652	11.2278	GDH	Medtr7g085630	NADP-specific glutamate dehydrogenase
1.18988	4.42316	NodGS	Medtr1g062710	nodulin/glutamate-ammonia ligase-like protein
1354.52	613.394	GGAT	Medtr5g033230	glutamate-glyoxylate aminotransferase
303.199	87.6004	GASA1	Medtr3g118070	glutamate-1-semialdehyde 2,1-aminomutase
13.6836	40.2263	GABARAP	Medtr2g023430	gaba-receptor-associated protein ubiquitin domain protein
59.1437	92.2045	GABARAP	Medtr2g088230	gaba-receptor-associated protein ubiquitin domain protein
78.0739	116.506	GABARAP	Medtr4g101090	gaba-receptor-associated protein ubiquitin domain protein
64.168	88.8288	GABARAP	Medtr4g048510	gaba-receptor-associated protein ubiquitin domain protein
30.1675	158.107	TPS	Medtr1g109620	trehalose-6-phosphate synthase domain protein
29.0621	129.856	TPS	Medtr1g032730	trehalose-6-phosphate synthase domain protein
20.2449	49.7794	TPS	Medtr0034s0170	trehalose-6-phosphate synthase
13.8054	21.4043	TPS	Medtr2g073260	trehalose-6-phosphate synthase
22.4012	16.7394	TPS	Medtr4g080160	trehalose-6-phosphate synthase domain protein
4.28908	2.14877	TPS	Medtr4g129270	trehalose-6-phosphate synthase domain protein
8.04799	3.02819	TPS	Medtr8g105740	trehalose-6-phosphate synthase domain protein
50.7712	20.4159	TPP	Medtr4g036090	trehalose-phosphate phosphatase
2.23129	4.37979	TPP	Medtr4g101600	trehalose-phosphate phosphatase
9.57638	5.49931	HK	Medtr8g014530	hexokinase
10.9137	5.73095	HK	Medtr6g088795	hexokinase
3.38202	13.023	PFK	Medtr2g100710	6-phosphofructokinase
0.525803	1.31515	PFK	Medtr5g032570	6-phosphofructokinase
11.2548	26.9884	PFK	Medtr4g134290	6-phosphofructokinase
24.4676	54.5847	PFK	Medtr8g102190	6-phosphofructokinase
23.1086	47.2095	PFK	Medtr6g090130	6-phosphofructokinase

11.2702	50.4042	PGM	Medtr8g006290	phosphoglycerate mutase family protein
18.34	13.0719	PGM	Medtr4g092840	phosphoglycerate mutase family protein
75.32	38.3234	PGM	Medtr2g013570	phosphoglycerate mutase
44.0105	22.3197	PGM	Medtr3g111310	phosphoglycerate mutase family protein
1.90495	0.857637	PGK	Medtr6g018680	phosphoglycerate kinase
7.66652	3.45295	PGK	Medtr2g095260	2-phosphoglycerate kinase
0.469678	1.00654	PGK	Medtr1g056720	2-phosphoglycerate kinase
1536.74	327.218	PGK	Medtr2g066110	phosphoglycerate kinase-like protein
1.86224	0.826396	PGK	Medtr6g011290	phosphoglycerate mutase-like protein
13.4134	7.83134	PGK	Medtr7g009060	phosphoglycerate mutase-like protein
682.462	299.553	PGLP	Medtr7g080530	phosphoglycolate phosphatase-like protein
56.0631	30.1008	PGDH	Medtr1g107480	D-3-phosphoglycerate dehydrogenase family protein
17.7314	52.2952	ALMT	Medtr5g014310	aluminum activated malate transporter family protein
2.89802	7.83636	ALMT	Medtr4g051575	aluminum activated malate transporter family protein
3.08243	7.52092	ALMT	Medtr4g098570	aluminum activated malate transporter family protein
119.282	44.3424	OMT	Medtr2g009220	2-oxoglutarate/malate translocator
164.826	35.2882	OMT	Medtr4g065007	2-oxoglutarate/malate translocator
45.4885	24.525	IPMS	Medtr1g116500	2-isopropylmalate synthase
51.0659	19.3914	IPMDH	Medtr2g104610	3-isopropylmalate dehydrogenase
464.035	112.462	gMDH	Medtr4g092690	glyoxysomal malate dehydrogenase
286.01	171.914	MDH	Medtr8g463760	malate dehydrogenase
190.785	108.574	MDH	Medtr1g076130	cytoplasmic-like malate dehydrogenase
265.539	150.151	MDH	Medtr1g043040	cytoplasmic-like malate dehydrogenase
11.191	4.49178	MDH	Medtr8g005980	cytoplasmic-like malate dehydrogenase
189.87	92.4863	ACLY	Medtr2g034090	ATP:citrate lyase
187.941	55.8366	ACLA-1	Medtr2g062920	ATP-citrate synthase alpha chain protein
30.176	48.9079	CSY3	Medtr3g048920	citrate synthase 3, peroxisomal protein
92.3021	123.627	IDH	Medtr5g077070	NADP-dependent isocitrate dehydrogenase
28.3778	9.77807	ASS	Medtr4g103680	argininosuccinate synthase
50.8709	23.5838	ADSL	Medtr7g092860	adenylosuccinate lyase
5.10698	2.51499	ADSL	Medtr5g081750	adenylosuccinate lyase
81.2238	48.7435	GLYR	Medtr8g028120	glyoxylate/succinic semialdehyde reductase
50.2928	13.4202	GLYR	Medtr2g099910	glyoxylate/succinic semialdehyde reductase
83.4239	177.931	SDH	Medtr5g020050	succinate dehydrogenase [ubiquinone] flavoprotein subunit
31.5611	45.7821	SDHB	Medtr1g492610	succinate dehydrogenase [ubiquinone] iron-sulfur subunit
59.6711	80.7958	SDHB	Medtr0018s0260	succinate dehydrogenase [ubiquinone] iron-sulfur subunit
53.2723	28.1818	ALPHA-SCS2	Medtr6g077820	succinyl-CoA ligase [ADP-forming] subunit alpha-2
12.9651	22.6348	BCCP	Medtr2g103280	biotin carboxyl carrier acetyl-CoA carboxylase
18.1025	9.43557	BCCP	Medtr6g015020	biotin carboxyl carrier acetyl-CoA carboxylase
28.0631	14.3313	BCCP	Medtr7g013100	biotin carboxyl carrier acetyl-CoA carboxylase
144.061	71.1014	BCCP	Medtr7g080290	biotin carboxyl carrier acetyl-CoA carboxylase
74.3424	36.076	ACC	Medtr8g101330	acetyl-CoA carboxylase biotin carboxylase subunit
5.35096	1.0041	accD	Medtr4g050863	acetyl-coA carboxylase carboxyltransferase beta subunit
64.9331	119.341	ACO	Medtr5g022940	cytoplasmic-like aconitate hydratase
13.7291	18.5585	ACO	Medtr4g048190	cytoplasmic-like aconitate hydratase
164.29	108.477	PGM	Medtr8g104540	cytoplasmic phosphoglucomutase
18.7936	36.5541	HPPD	Medtr5g091060	4-hydroxyphenylpyruvate dioxygenase
6.32472	9.93728	HPPD	Medtr5g090980	4-hydroxyphenylpyruvate dioxygenase
9.35988	18.592	PGLS	Medtr5g045000	glucosamine-6-phosphate isomerase/6-phosphogluconolactonase
57.3965	19.8764	PGLS	Medtr1g028320	glucosamine-6-phosphate isomerase/6-phosphogluconolactonase
51.7187	13.3864	PGLS	Medtr1g028330	glucosamine-6-phosphate isomerase/6-phosphogluconolactonase
159.017	116.395	PGD	Medtr7g017900	decarboxylating-like 6-phosphogluconate dehydrogenase
75.4566	53.3728	PGD	Medtr8g099185	decarboxylating-like 6-phosphogluconate dehydrogenase
6.74328	4.37057	PGDH	Medtr8g088900	6-phosphogluconate dehydrogenase-like protein
57.4216	24.6736	FH	Medtr1g087900	fumarate hydratase
2130.43	652.241	GAPDH	Medtr3g115920	glyceraldehyde-3-phosphate dehydrogenase B
4855.69	1115.1	GAPDH	Medtr7g084800	glyceraldehyde-3-phosphate dehydrogenase
0.687058	3.07768	NADP-GAPDH	Medtr1g014320	NADP-dependent glyceraldehyde-3-phosphate dehydrogenase
1028.75	443.604	RPE	Medtr4g108800	ribulose-phosphate 3-epimerase
58.0905	27.4291	Rubisco	Medtr4g021210	ribulose bisphosphate carboxylase/oxygenase activase
92.5537	29.1866	Rubisco	Medtr2g105480	ribulose bisphosphate carboxylase/oxygenase activase
86.8246	5.19883	RBCS	Medtr7g007200	ribulose bisphosphate carboxylase small chain
128.607	6.21938	RBCS	Medtr7g007120	ribulose bisphosphate carboxylase small chain
40.1342	0.88186	RBCS	Medtr7g007230	ribulose bisphosphate carboxylase small chain
25.8692	7.63572	RBCL	Medtr4g051270	ribulose bisphosphate carboxylase large chain domain protein
0.780575	0.198257	RBCL	Medtr6g055010	ribulose bisphosphate carboxylase large chain domain protein
19.7333	70.6749	PEPEC	Medtr2g092930	phosphoenolpyruvate carboxylase
45.8304	66.0523	PEPEC	Medtr1g094010	phosphoenolpyruvate carboxylase
38.4137	54.632	PEPEC	Medtr2g076670	phosphoenolpyruvate carboxylase
57.0682	78.2569	PEPEC	Medtr1g094000	phosphoenolpyruvate carboxylase
69.6138	40.679	PEPEC	Medtr8g463920	phosphoenolpyruvate carboxylase
3.28823	1.25575	PEPEC	Medtr4g079860	phosphoenolpyruvate carboxylase
0.529471	1.03165	PCKA	Medtr5g430730	phosphoenolpyruvate carboxykinase [ATP] protein
3.70197	5.69649	PDC	Medtr2g015560	pyruvate decarboxylase
0.339392	0.108399	PDC	Medtr7g069540	pyruvate decarboxylase
0.607883	0.043344	PDC	Medtr2g009330	pyruvate decarboxylase
0.385012	0.159089	PDC	Medtr5g046610	pyruvate decarboxylase
28.2199	20.0905	PK	Medtr6g004020	pyruvate kinase family protein
54.5871	25.9717	PK	Medtr1g076540	pyruvate kinase family protein
47.2312	18.8652	PK	Medtr6g034195	pyruvate kinase family protein
18.1967	5.07857	PK	Medtr1g105965	pyruvate kinase family protein
1.95628	0.242755	PK	Medtr4g083340	pyruvate kinase family protein
7.92729	25.968	PPDK	Medtr4g118350	pyruvate orthophosphate dikinase
20.3592	53.9382	Enolase	Medtr6g060570	phosphopyruvate hydratase
339.055	243.955	Enolase	Medtr7g103620	phosphopyruvate hydratase
47.4263	26.195	PDK	Medtr7g024460	pyruvate dehydrogenase (acetyl-transferring) kinase
14.3581	7.59767	PDH2	Medtr3g076630	pyruvate dehydrogenase E1 beta subunit
40.6262	60.0095	PDHA1	Medtr5g036600	pyruvate dehydrogenase complex E1 alpha subunit
312.049	89.3826	GRHPR	Medtr1g112510	D-glycerate dehydrogenase/hydroxypyruvate reductase
0.732055	0.155978	GRHPR	Medtr4g013135	D-glycerate dehydrogenase/hydroxypyruvate reductase
34.4808	68.0237	GRHPR	Medtr6g007170	D-glycerate dehydrogenase/hydroxypyruvate reductase
